# Evolutionary analysis of stakeholder behavior in green retrofitting of traditional residential buildings based on dissemination and game models

**DOI:** 10.1371/journal.pone.0282314

**Published:** 2023-03-16

**Authors:** Yaohong Yang, Ruicong Sun, Jing Dai, Mengjuan Zhu

**Affiliations:** 1 School of Water Conservancy, North China University of Water Resources and Electric Power, Zhengzhou, China; 2 The Henan Key Laboratory of Water Resources Conservation and Intensive Utilization in the Yellow River Basin, Zhengzhou, Henan, China; University of Georgia, UNITED STATES

## Abstract

To achieve carbon peaking and carbon neutrality goals in China, green retrofitting of traditional residential buildings is the one of the important ways. Therefore, the dynamics process of the change of the resident group intention to retrofit and its impact on the behavior of the tripartite game between the government, investment retrofitting enterprises and residents needs to be analyzed. Firstly, a dissemination model of green retrofitting intentions among resident groups is constructed, and it is coupled with the tripartite game model. Then, through numerical simulation, the dissemination laws of intention for green retrofitting among resident groups and its influence on the evolution process of the tripartite game are analyzed. The results show that: (1) The rate at which the triad of government, investment retrofitting enterprises and residents reaches steady state is influenced by the impact of changes in the level of social climate on the rate of conversion of potential and participating residents to immune residents. When the rate of enterprises investment and residents participation increases, the rate of government choice of incentive strategies decreases; (2) greater government regulation and subsidies will increase the intention of residents and retrofitting enterprises to participate. The ideal steady state without government incentives can be achieved when the group size of participating residents is increased by improving the level of government publicity and education and the technology level of the enterprises; (3) the intention of enterprises to invest is closely related to the cognitive benefits and the level of risk perception of residents. The conclusions of the study can be used as a reference for the government to make green retrofitting policies for traditional residential buildings.

## 1 Introduction

Climate anomalies and environmental problems caused by excessive greenhouse gas emissions have become a global challenge. China is the world largest emitter of CO_2_ in the global construction sector [1], and the whole life cycle carbon emissions of the Chinese construction sector account for 42% of the national carbon emissions [2]. Therefore, the construction sector has become an important area on carbon emission reduction in China. Focusing on the Chinese construction sector, carbon emissions from the Chinese construction sector are mainly attributed to the operational phase of existing buildings [3–7]. Therefore, green operation of existing buildings is crucial. However, the "China Building Energy Consumption Annual Research Report 2020" data show that China’s building stock was 67.4 billion m^2^ in 2018, of which urban residential building area was 30.7 billion m^2^ and rural residential building area was 23.8 billion m^2^, which together accounted for more than 80%, but green building area was only 2.5 billion m^2^, accounting for only 4% of the building stock. This results in carbon emissions of 891 million tons CO_2_ for urban residential buildings and 437 million tons CO_2_ for rural residential buildings, accounting for 42.2% and 20.7% of carbon emissions in the building operation phase, respectively [8, 9]. Therefore, the operation mode of existing residential buildings needs to be changed at present. Compared to demolition and reconstruction, green retrofitting can increase the efficiency of energy and resource use in existing buildings, and is essential to reduce carbon emissions and environmental load [10, 11]. Therefore, green retrofitting provides a fast and effective means of reducing energy consumption and greenhouse gas emissions [12]. In order to take a high quality development path with low energy consumption, low pollution and low emissions and to achieve the goal of carbon peaking and carbon neutrality in Chinese construction sector, the green retrofitting of traditional residential buildings has become one of the important ways to reduce emissions in the current construction sector.

The participation of residents is a prerequisite for the green retrofitting of traditional residential buildings [13]. Only the effective participation of residents on the consumer side can drive investment by enterprises on the supply side and promote the development of green retrofitting projects for traditional residential buildings. Consumers’ purchasing decisions are influenced by information and knowledge [14]. Positive information can guide and persuade residents on the consumer side to participate in green retrofitting. Therefore, the dissemination of information is an important way to promote the participation of residents in green retrofitting [15]. Existing studies have analyzed the factors that influence residents’ intention to green retrofit [16–18]. However, the action mechanisms of the different factors are not discussed in depth within the resident groups. Green retrofitting of traditional residential buildings involves multiple groups such as government, investment retrofitting enterprises, residents, material suppliers, third-party supervisors and auditors. The government mainly adjusts the development of the green retrofitting market of traditional residential buildings through incentive and regulation; the investment retrofitting enterprises are the supply side of the market by providing green retrofitting services to obtain economic benefits; the residents are the demand side of the market and their lack of motivation to participate largely leads to a sluggish market. Therefore, the government, investment retrofitting enterprises and residents are the core main bodies of green retrofitting of traditional residential buildings. The synergistic development of these three parties drives the stability of the green retrofitting market. Social climate level, the level of government regulation, subsidies and publicity and education, the technology level of enterprises, and the cognitive benefits and risk perception of residents influence the balance of benefits and costs among the three parties in the economic activities of green retrofitting, thus affecting the enthusiasm of government incentives, enterprises’ investment and residents’ participation. This indicates that the green retrofitting of traditional residential buildings is the result of mutual checks and balances among the conflicting interests of three parties at the same time. Therefore, the main bodies of the green retrofitting of traditional residential buildings contain a game relationship, and what kind of law the government, investment retrofitting enterprises and residents follow to evolve and develop directly affects the developing trend of the green retrofitting market of traditional residential buildings [19, 20]. However, it is assumed that the resident group is homogeneous in the existing game analysis. In fact, the resident group is a heterogeneous group. Individuals are affected by multiple factors and their intention to retrofit is often heterogeneous. The dissemination of residents’ intention to green retrofit is influenced by multiple internal and external factors, and has a complex impact on tripartite benefits, so the heterogeneity of the resident group needs to be taken into account. Therefore, this paper uses a dissemination model to describe the dynamic dissemination process of residents’ intention to green retrofit, and introduces the dissemination model into the evolutionary game model to study the influence of the dissemination process among resident groups on the evolutionary law of the strategic choice of the government, investment retrofitting enterprises and residents.

## 2 Literature review

### 2.1 Technology management and policy for green retrofitting of traditional buildings

In terms of technology management, the main focus has been on the optimal combination of technology methods, such as research on the optimal combination of energy efficiency measures for traditional buildings [21], research on the optimization of the thermal performance of the envelope of energy-efficient retrofit traditional residential buildings [22], and research on the combination of technologies for energy efficiency retrofitting and heritage conservation of historic buildings [23]. In addition, simulation methods have also been used to assess the performance risk of contract energy management projects [24] and to design contract decision-making models between building owners, Energy Service Companies (ESCOs) and renters [25]. Given the uncertainty of retrofit projects, fuzzy multi-attribute decision making and Monte Carlo analysis can be combined to predict large-scale building energy retrofit investments [26], and life-cycle costing methods and Monte Carlo simulations can be combined to analyze the impact of uncertain parameters such as discount rates and energy prices in assessing the benefits of building energy retrofits [27].

Government policy guidance plays an irreplaceable role in maximizing the benefits to society and is the most direct and effective method of addressing carbon emissions currently [28, 29]. Murphy focused on the effectiveness and lessons learned from the implementation of energy efficiency retrofit policy tools in European front-runners [29]. Sebi et al. presented a comparative summary of the implementation, successes and challenges of retrofitting policies in the US, Germany and France [30]. Li et al. summarized the current situation and barriers to green retrofitting of public buildings based on a survey of 10 typical public buildings in different climatic zones in China [31]. Tan et al. developed a basic framework for implementing suitable green retrofitting technologies and policies in Hong Kong based on the climate as well as building characteristics [32]. Ergin et al. identified energy and cost reduction and building energy performance improvement as the three main benefits of green retrofitting in healthcare buildings through frequency analysis under four dimensions: environmental, economic, social and functional [33].

### 2.2 Influencing factors and stakeholders’ strategies for the retrofitting of traditional buildings

The supply side, demand side and market environment for green retrofitting of traditional buildings are influenced by a combination of factors. The main factors affecting the effectiveness of policies are different financing and procurement models [34], unit costs and energy prices [35]. Laws and regulations, information sharing, publicity and education and awareness of responsibility can influence the operation of the green retrofit market in traditional residential areas [36], with policy regulations and technology improvements more likely address complex issues such as climate change than raising public awareness and individual behavior [37]. On the demand side, policy factors not only indirectly influence residents’ intention to retrofit through perceived behavioral control, but also are the most important factors directly influencing residents’ intention to retrofit [38, 39] in addition to geography [40], building lifespan [41], living habits [42], time [43], economic costs [44, 45], income [46], comfort and convenience, ethics, technological novelty [47], occupants’ psychological perceptions [48] and social norms [49, 50], which also influence residents’ intention to retrofit. The study of success factors in the implementation of green retrofit projects should also be addressed [51]. However, most of the above studies have empirically identified the internal and external factors which influence residents’ pro-environmental behavior, but do not consider the synergistic mechanisms of these factors and describe the dissemination laws among resident groups.

Evolutionary game theory was originally proposed in the field of biology to describe the dynamic evolutionary process of survival and competition among various biological populations. The theory assumes that the members of the group do not have rational thinking and can only acquire limited knowledge through learning to constantly adjust their strategies until all game groups reach a stable dynamic equilibrium. And the theory focuses on analyzing the dynamic evolution of behavioral strategies of game players. Since there are commonalities between the evolution of biological populations and the behavior of cooperation and contradiction of humans in economic activities, the mechanisms and laws of biological evolution in evolutionary game theory can be used to explain and describe the underlying mechanisms of human dynamic behavior. Therefore, the evolutionary game method has been used in most studies on the behavioral strategies of stakeholders in the retrofitting market of traditional buildings. The analysis is developed from the perspective of the type of retrofitting of traditional buildings. One analyzes the strategic choices of the participants from the perspective of green retrofit. The study from the game strategy of the government and green retrofitting investment groups found that a combination of positive and negative policy measures was most appropriate to promote green retrofitting for PPP-BR [52]. Optimal strategic choices of the participants were obtained for the study of optimal incentive strategies between government and developers in green retrofitting of large public buildings [53] and the study of optimal cooperation strategies between government, private sector and owners in retrofitting of old communities under the PPP model [54]. The main reasons affecting the stakeholders’ green retrofitting decisions were revealed based on the green retrofitting decision-making behavior of owners and renters under different occupancy types [55]. The green retrofitting behavioral strategies of the government, ESCOs and owners were analyzed by studying their tripartite green retrofitting game [56]. It can be found that the behavioral strategies of the tripartite players influence each other and thus change the final evolutionary path [57]. The government’s incentive intensity, within a reasonable range, was effective in increasing the motivation of ESCOs and owners to implement retrofits [58], and could solve the green retrofit financing dilemma [59]. The other analyzes the strategic choices of the participants from the perspective of energy efficiency. The game analysis between the government and ESCOs proposed effective measures to optimize the government’s regulatory function, such as improving incentive and constraint mechanisms and standardizing building energy efficiency regulatory standards [60], and energy-using units and ESCOs could optimally allocate energy efficiency benefits under an energy efficiency benefit sharing model [61]. Factors such as benefits, costs, risks and government regulation influenced the outcome of the game between green retrofit owners and ESCOs [62].

From the perspective of the game players of the retrofitting of traditional buildings, scholars mainly analyze the equilibrium of the game between two or three players. One is a two-party game model. Scholars discuss the process of strategy evolution between players such as government and investment groups [52], government and developers [53] owners and renters [55], government and ESCOs [60], energy-using units and ESCOs [61], and owners and ESCOs [62], respectively. The other is a three-party game model with more comprehensive research main bodies. Guo et al. constructed a tripartite evolutionary game model of the government, private sector and owners to improve the operational effectiveness of the PPP model in the retrofitting of old communities, and found that the participation of owners helped accelerate the formation of a tripartite cooperation situation [54]. Other scholars focus on studying the game between the government, ESCOs and owners. For example, Chen et al. introduced prospect theory into the tripartite evolutionary game model of government, ESCOs and owners to explore the optimal strategy of the tripartite game in green retrofitting of existing buildings under different values of parameters [56]. Wu et al. found that the government, ESCOs and owners do not converge on a stable set of strategies under information asymmetry in the energy efficiency retrofit market of existing buildings [57]. Yang et al. analyzed the externalities of energy efficiency retrofitting of existing civil buildings and constructed a tripartite evolutionary game model with government incentives for ESCOs and owners based on the principle of fairness to explore how to motivate players to participate in energy efficiency retrofitting [58]. Therefore, for the green retrofitting of traditional residential buildings, the process of strategy change among the government, investment retrofitting enterprises and residents can be analyzed more carefully with the use of the three-party game model.

From the above studies, it can be seen that residents, as the core main body of pulling green consumption, are covered in most of the literature, but the resident group is considered as an individual because they assume that the individuals in the resident group are non-differentiated. This has the advantages of simplifying the complexity of individuals in the resident group and simplifying the description of the group’s behavior. However, this method also has the disadvantage that its assumption of individual homogeneity in the resident group does not reflect individual differences and ignores the influence of complex interaction processes between individuals within the population on the evolution of the system. In fact, residents are non-homogeneous groups. Residents’ intention to retrofit often has subjective characteristics and individual diversity depending on their cognitive level and their objective conditions, and the interaction between individual residents influences the whole game system. Therefore, it is necessary to consider the differences of individuals in the resident group, but the traditional evolutionary game cannot be used to describe them [63]. In this regard, the epidemic model more realistically characterizes the complex interactions between individuals in the population to overcome this deficiency.

### 2.3 Application of dissemination models

Epidemic models were first used to study the mechanisms of the dissemination of diseases in the medical field. These models classify populations into nodes based on their infection status. Influenced by the dissemination of the virus, the status of individuals may change. Epidemic models mainly include the classic models such as the *SI* model [64], the *SIS* model [65] and the *SIR* model [66], as well as improved models such as the *SIRS* model [67], the *SIHR* model [68], the *SHIR* model [69] and the improved *SIR* model with infection age and saturated incidence [70]. Among them, the *SIR* model is the earliest and the most classic dissemination model. In this model, the population is divided into three states: susceptible, infected and immune, and each of these is considered as a node. The susceptible node represents a population that is not yet infected but has the potential to become infected. The infected node represents a population that is already infected and disseminating the disease. The immune node represents a population that has been cured and is no longer infected. In recent years, scholars have applied epidemic models to study different problems, and have produced a relatively rich amount of research results. Guo et al. used the *SIS* model and its stochastic differential equation model with multiplicative noise terms to study the effect of stochastic perturbations on disease outbreaks under media coverage [71]. Zhu et al. studied the influence of the interaction of multiple publicity and communication channels on the spread of awareness of COVID-19 vaccination among the public based on the modeling idea of the dissemination dynamics model [72]. Hu et al. improved the *SIR* model by combining the differences in scale and status of enterprises in industry clusters, that is, enterprises with the attribute of being disseminated were divided into big enterprises and small enterprises, and the mechanism of knowledge dissemination among heterogeneous enterprises was explored in depth [73]. Ma et al. introduced the complement of sub-infectors as dissemination main bodies in the *SIR* model to make suggestions for addressing the barriers to reading promotion in university libraries [74]. Le et al. constructed a dissemination dynamics model for the generation to decay of turnover information in labor organizations to explain the collectivization of labor turnover, and governance strategies to inhibit the infection of turnover were proposed [75]. Similarly, in the green retrofitting of traditional residential buildings, residents’ intention to green retrofit interacts within the group through dissemination. Therefore, the heterogeneity of residents can be described by classifying the status of individuals according to the difference of residents’ intention to retrofit, based on the idea of population classification by the epidemic model. Because the dissemination of information of green retrofitting among residents is relatively similar to the mechanism of dissemination and diffusion of diseases in the population, the epidemic model is applicable to study the process of change of residents’ intention to green retrofit. Firstly, the states of the nodes in the dissemination process are similar. The nodes are human. The infected patient carrying the virus is infectious in the dissemination of infectious diseases, and the resident disseminating information of green retrofitting is also infectious. Secondly, the characteristics of the disseminated content are similar. Infectious diseases disseminate viruses. Residents disseminate information regarding their intentions to green retrofit. Both viruses and information have the characteristic of replication and dissemination and thus influence the change of human status. Finally, the ways of dissemination are similar. Contact is the main way to disseminate infectious diseases, and the information of green retrofitting is also disseminated through oral contact between residents. The frequent interaction and close communication between residents accelerate the speed of information diffusion, which makes the conversion of residents’ intention more likely. In addition, the dissemination of viruses can be done through mediums, such as air and water. The information of green retrofitting of residents can also be disseminated through mediums, such as WeChat, YouTube and other network mediums. Information exchange is also necessarily present in the residents’ social network and has a decisive influence on the decision to participate in the green retrofit [76–78]. Therefore, the epidemic model can be used to describe the process of change of residents’ intention to green retrofit. Among the epidemic models, the *SIR* model is chosen as the base model because of its good fit with the dissemination mechanism of residents’ intention to green retrofit. However, compared with the *SIR* model, residents’ intention has diversified characteristics, and there are situations where individuals with different intentions co-exist and disseminate. At the same time, they may change the original intention by interacting with each other. Therefore, residents with different intentions may be converted to each other. Second, in the traditional *SIR* model, the susceptible population is infected with a certain probability after being exposed to the infected population. However, in the resident group, a part of the residents may not be interested in the green retrofit after receiving the information. Therefore, they are not influenced by residents who have emotions and choose not to disseminate information about green retrofitting. Moreover, in the traditional *SIR* model, the conversion process of the state is unidirectional, i.e., infected individuals can only infect susceptible individuals, and immune individuals cannot be infected again and keep the state because they have immunity. However, the dissemination process of residents’ intention to green retrofit is bidirectional. This is because the diffusion of information about green retrofitting may arouse the interest of residents who were not paying attention to green retrofitting, and thus green retrofitting will be concerned again. Therefore, their attitudes may change. Based on the above analysis, it is obvious that the simple use of the *SIR* model to describe the dissemination process of residents’ intention to green retrofit has certain limitations. The *SIR* model needs to be improved in terms of the node state attributes and the dissemination process, which is different from the existing studies [71–75].

To provide a theoretical basis for promoting the development of green retrofitting of traditional residential buildings, a dissemination model of residents’ intention to green retrofit is constructed by considering the factors influencing residents’ intention. And evolutionary game theory is used as the analysis method to construct the evolutionary game model based on the process of change of residents’ intention on the demand side in the green retrofitting market of traditional residential buildings and the interests of the three game players: government, investment retrofitting enterprises and residents. By coupling the dissemination model with the tripartite game model, the dynamics of the intention to retrofit within the resident group and its impact on the steady state of tripartite evolution are analyzed, and the strategic choices of the game players are discussed.

## 3 Model construction

**Fig 1:**
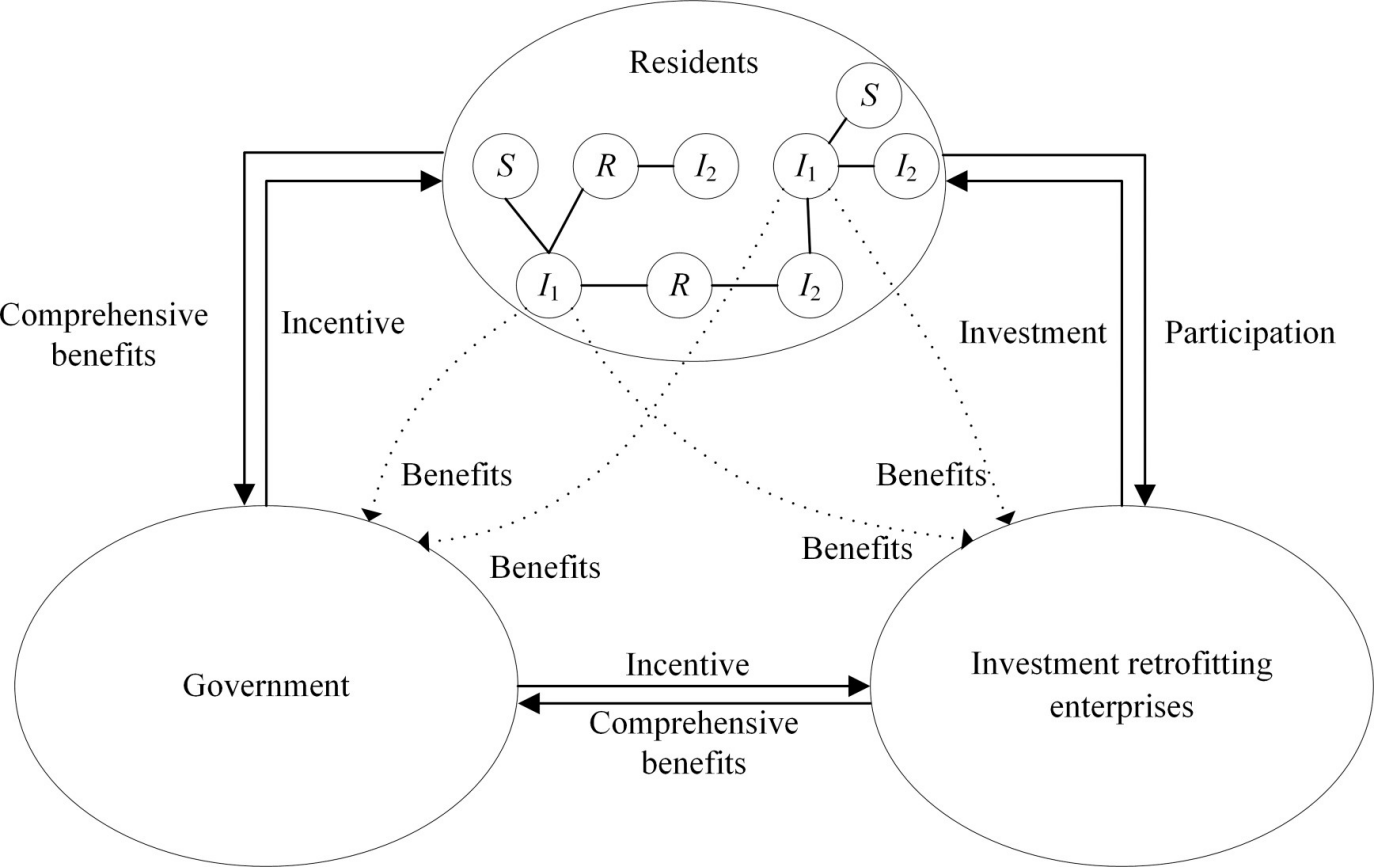
A framework for understanding players’ decision-making.

The green retrofitting of traditional residential buildings contains three game players: government, investment retrofitting enterprises and residents, and the green retrofitting of traditional residential buildings is smoothly promoted by the behavioral interaction of these three parties. At the same time, the resident group is a heterogeneous group. The dissemination of intention to green retrofit among residents has an impact on the players. Based on the above, the framework for understanding players’ decision-making is shown in Fig 1. And the various parameters of the dissemination and evolutionary game models are shown in Table 1.

**Table 1:** Parameters of the dissemination and evolutionary game models.

Category	Variable	Meaning	Unit
State variable	*S*	Potential residents: The number of residents who lack awareness of environmental responsibilities, and have insufficient cognitive ability or some level of understanding of green retrofitting, but do not participate in it.	pp
State variable	*I*_*p*_	Participating residents: The number of residents who have the knowledge to actively participate in green retrofitting and spread information and knowledge about green retrofitting.	pp
State variable	*I*_*a*_	Resistant residents: The number of residents who refuse to participate in green retrofitting because of cognitive bias and resistance to it, and who spread misinformation about green retrofitting.	pp
State variable	*R*	Immune residents: The number of residents who have knowledge of green retrofitting but are not interested in it and are blocking information on green retrofitting.	pp
Parameter	*N*	Total number of residents in traditional residential buildings.	pp
Parameter	*α*	Average number of residents reached by a single resident per unit of time.	pp/d
Parameter	*β*_1_	Success rate of conversion of potential residents into participating residents.	
Parameter	*β*_2_	Success rate of conversion of potential residents into resistant residents.	
Parameter	*o*	The level of awareness of environmental responsibility of the residents.	
Parameter	*k*	The level of risk perception of the residents.	
Parameter	*b*	The level of knowledge of residents about green retrofitting.	
Parameter	*l*	The technology level of the enterprises.	
Parameter	*h*	Social climate level. In this paper it mainly refers to a tendency of social environment that is generally supportive of green retrofitting formed among the public.	
Parameter	*q*	The level of government publicity and education.	
Parameter	*u*	The strength of government regulation on the whole process of green retrofitting.	
Parameter	*n*	The intensity of the subsidy given to residents participating in green retrofitting when government incentivizes.	
Parameter	*m*	The intensity of the subsidy given to investment retrofitting enterprises when government incentivizes.	
Parameter	*E*_1_	The comprehensive benefits to the government, such as improved air quality, protected environment and reduced resource waste, are obtained when the government incentivizes, enterprises invest and residents participate in green retrofitting.	
Parameter	*E*_2_	The comprehensive benefits to the government, such as improved air quality, protected environment and reduced resource waste, are obtained when the government does not incentivize, businesses invest and residents participate in green retrofitting.	
Parameter	*lE*_3_	The benefits obtained by enterprises when they invest and residents participate in green retrofitting.	
Parameter	*E*_4_	The cognitive benefits for residents to participate in green retrofitting.	
Parameter	*qC*_1_	The publicity cost for the government.	
Parameter	*uC*_2_	The regulation cost to the government when government incentives, enterprises invest, and residents participate in green retrofitting.	
Parameter	*C*_3_	The cost to enterprises when they invest and residents participate in green retrofits.	
Parameter	*C*_4_	Early information exchange costs paid by enterprises when they invest and residents do not participate in green retrofitting.	
Parameter	*kC*_5_	The perceived cost for residents to participate in green retrofitting.	
Parameter	*mB*_1_	Subsidies given to investment retrofitting enterprises when government incentivizes.	
Parameter	*nB*_2_	Subsidies given to residents participating in green retrofitting when government incentivizes.	
Parameter	*W*	Reputation obtained when government incentivizes.	
Parameter	*G*_1_	Extra benefits such as enhanced competitiveness and increased customers are obtained from enterprises’ investments when the government incentivizes and residents participate.	
Parameter	*G*_2_	Extra benefits such as enhanced competitiveness and increased customers are obtained from enterprises’ investments when the government does not incentivize and residents participate.	
Parameter	*F*_1_	Negative impacts such as loss of customers and damage to image suffered by enterprises not investing when government incentives and residents participate.	
Parameter	*F*_2_	The losses suffered by enterprises not investing when government does not incentivize and residents participate.	
Parameter	*P*_1_	The mental satisfaction benefits obtained by residents who participate in green retrofitting when government incentivizes and enterprises invest.	
Parameter	*P*_2_	The mental satisfaction benefits obtained by residents who participate in green retrofitting when government does not incentivize and enterprises invest.	
Parameter	*D*_1_	The group pressure on residents not to participate in green retrofitting when government incentivizes.	
Parameter	*D*_2_	The group pressure on residents who do not participate in green retrofitting when the government does not incentivize.	
Parameter	*J*	The spiritual rewards obtained by residents who participate in green retrofitting when the government incentivizes and enterprises do not invest.	

### 3.1 Construction of the dissemination model

The dissemination of residents’ intention to green retrofit has its special characteristics and laws. Therefore, the improved *SIR* dissemination model of residents’ intention to green retrofit is established on the basis of the traditional *SIR* model. First, the dissemination nodes are divided into two categories of participating residents (*I*_*p*_) and resistant residents (*I*_*a*_), and two new conversion paths are added, i.e., from *I*_*p*_ to *I*_*a*_ and from *I*_*a*_ to *I*_*p*_. Second, the path for the conversion of potential residents (*S*) to immune residents (*R*) is added, i.e., from *S* to *R*. Finally, the conversion paths from the immune node to the dissemination nodes are added to reflect the fact that a part of the responding population among the immune residents is still affected, i.e., from *R* to *I*_*p*_ and from *R* to *I*_*a*_. The basic assumptions are described in Section 3.1.1.

#### 3.1.1 Basic assumptions

Assumption 1: The residents of traditional residential buildings are divided into the following four states: *S*, *I*_*p*_, *I*_*a*_ and *R*. The total number of residents remains constant at a certain time, namely $S+I_{p}+I_{a}+R=N$.

Assumption 2: The dissemination process of residents’ intention to green retrofit is influenced by a combination of internal and external factors. Internal factors include residents’ level of awareness of environmental responsibility, knowledge of green retrofitting, cognitive benefits and perceived risks of green retrofitting; external factors include the government’s level of publicity and education, regulation of green retrofitting, subsidies to residents, the technical level of enterprises and the level of social climate [48, 79–86]. The influencing factors are applied to the dissemination model, and are used to represent the conversion process between residents of various states. The rate variable is used to quantify the probability of conversion.

Assumption 3: Assume that *β*_1_>*β*_2_, which means that residents have a higher success rate of being converted by positive messages compared to negative messages. Therefore, the rate of conversion of potential residents into participating residents per unit time at moment *t* is set to $\frac{\alpha \beta_{1}oS_{i}I_{pi}}{N}$, and the rate of conversion of potential residents into resistant residents is set to $\frac{\alpha \beta_{2}(1-o)S_{i}I_{ai}}{N}$ (*i* = 1,2).

Assumption 4: The number of potential, participating, resistant and immune residents in the population are *S*_*i*_, *I*_*pi*_, *I*_*ai*_ and *R*_*i*_ respectively, and the initial moment *S*_0_ = *S*_*i*_, *I*_*p*0_ = *I*_*pi*_, *I*_*a*0_ = *I*_*ai*_, *R*_0_ = *R*_*i*_ (*i* = 1,2), where *i* = 1 represents the number of residents in each state when the government incentivizes and *i* = 2 represents the number of residents in each state when the government does not incentivize.

Assumption 5: The rate of conversion of immune residents to participating residents is increased and the rate of conversion of participating residents to immune residents is reduced as a result of government subsidies and the regulation of green retrofitting. Therefore, it is assumed that the rate of conversion of immune residents to participating residents is greater at the initial moment when government incentivizes than with government does not, and the rate of conversion of participating residents to immune residents is less when government incentivizes than with government does not.

#### 3.1.2 Model construction. The conversion processes between residents in different states are shown in Fig 2

**Fig 2:**
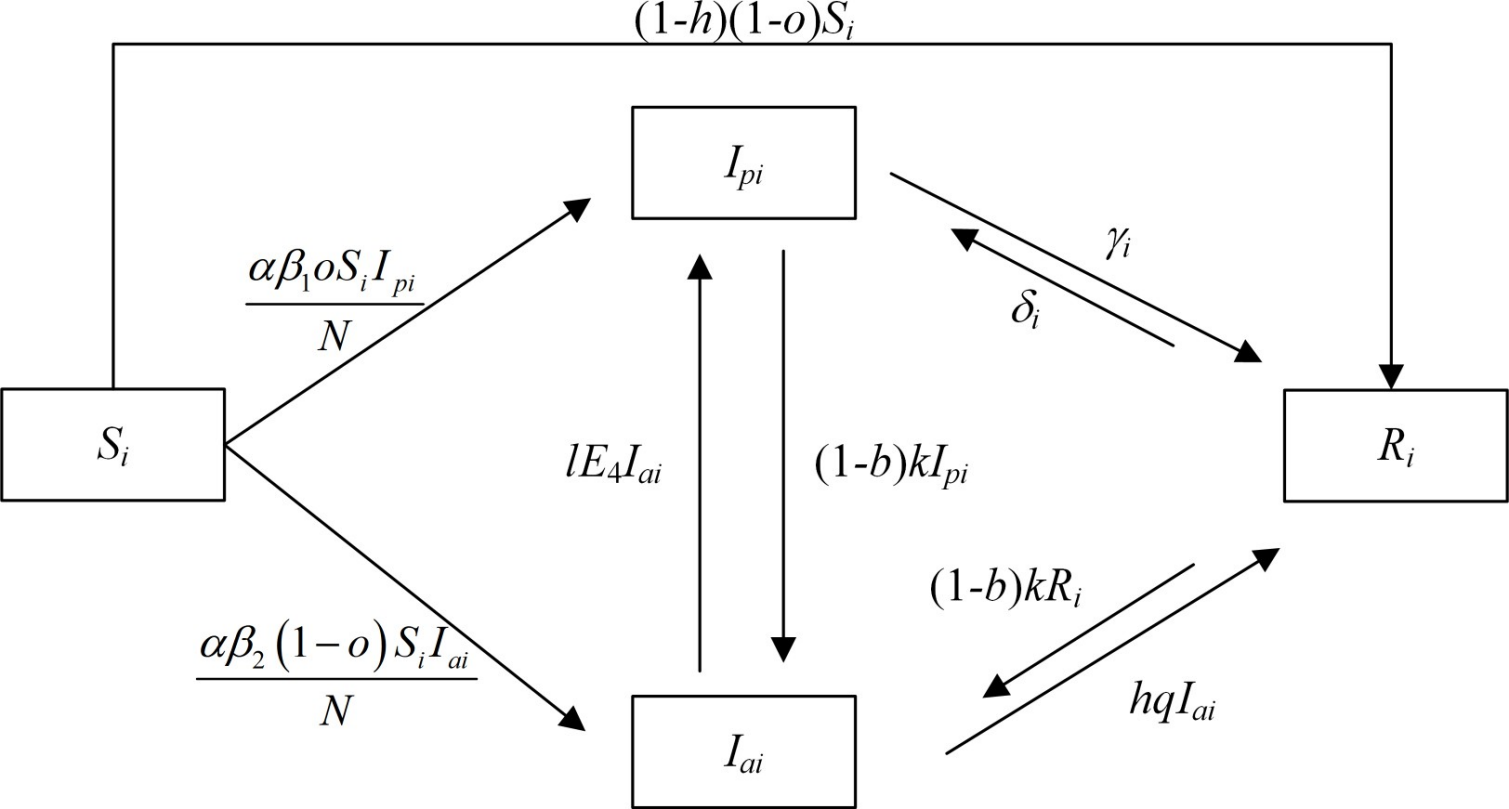
The *SIR* dissemination model of green retrofit intention among residents.

Accordingly, the corresponding sets of dynamic differential equations for the green retrofit intention of residents are established as shown below:

$$\left\{ \begin{array}{l} \frac{dS_{i}}{dt}=-\frac{\alpha \beta_{1}oS_{i}I_{pi}}{N}-\frac{\alpha \beta_{2}(1-o)S_{i}I_{ai}}{N}-(1-h)(1-o)S_{i}\\ \frac{dI_{pi}}{dt}=\frac{\alpha \beta_{1}oS_{i}I_{pi}}{N}+lE_{4}I_{ai}+\delta_{i}-(1-b)kI_{Pi}-\gamma_{i}\\ \frac{dI_{ai}}{dt}=\frac{\alpha \beta_{2}(1-o)S_{1}I_{a1}}{N}+(1-b)kI_{pi}+(1-b)kR_{1}-lE_{4}I_{ai}-hqI_{ai}\\ \frac{dR_{i}}{dt}=(1-h)(1-o)S_{i}+\gamma_{i}+hqI_{ai}-\delta_{i}-(1-b)kR_{i} \end{array}\right.$$ (1)

The Eq (1) represents the conversion of the residents in the four states to each other, that is, the process by which the number of residents in each state varies with time *t*. As $\frac{d(S_{i}+I_{pi}+I_{ai}+R_{i})}{dt}=\frac{dN}{dt}=0$, $N(t)=S_{i}(t)+I_{pi}(t)+I_{ai}(t)+R_{i}(t)=N$ is obtained, where: *i* = 1,2, $\gamma_{1}=(1-h)(1-u)I_{p1}$, $\gamma_{2}=(1-h)(1-q)I_{p2}$, $\delta_{1}=nE_{4}R_{1}$, $\delta_{2}=lE_{4}R_{2}$.

The conversion process between residents in various states in this model is described as follows: *S*_*i*_ are converted to *I*_*pi*_ at a rate of $\frac{\alpha \beta_{1}oS_{i}I_{pi}}{N}$ due to their level of environmental responsibility awareness or exposure to information and knowledge about green retrofitting of *I*_*pi*_, or they are converted to *I*_*ai*_ at a rate of $\frac{\alpha \beta_{2}(1-o)S_{i}I_{ai}}{N}$ due to biased information and negative emotions about green retrofitting, or they are converted to *R*_*i*_ at a rate of $(1-h)(1-o)S_{i}$ when they lose interest in green retrofitting at a low level of social climate; *I*_*pi*_ are converted to *R*_1_ at a rate of $(1-h)(1-u)I_{p1}$ due to low levels of regulation and social climate when government incentivizes, or to *R*_2_ at a rate of $(1-h)(1-q)I_{p2}$ due to low levels of public education and social climate when government does not incentivize; *R*_*i*_ who are exposed to the negative information of *I*_*ai*_ lead to an increase in the level of risk perception and refuse to participate and are converted to *I*_*ai*_ at the rate of $(1-b)kR_{i}$; *I*_*pi*_ are directly converted to *I*_*ai*_ at the rate of $(1-b)kI_{pi}$ when they are incited by the negative emotions of *I*_*ai*_, the forgetfulness of the knowledge of green retrofitting and the strong influence of risk perception; the intention of participating residents to green retrofit spreads in the process of dissemination to increase the residents’ knowledge of green retrofitting and the social climate, so that *I*_*ai*_ gradually correct their misconceptions and are converted to *R*_*i*_ at the rate of *hqI*_*ai*_ under the good social climate and government publicity and education; *R*_1_ are converted to *I*_*p*1_ at the rate of *nE*_4_*R*_1_ when the government strengthens subsidies and increases cognitive benefits after being exposed to information about the green retrofitting by *I*_*p*1_, or *R*_2_ are converted to *I*_*p*2_ at a rate of *lE*_4_*R*_2_ when the government does not incentivize but the technical level of green retrofitting by enterprises enhances; *I*_*ai*_ change their cognitive benefits after exposure to information and knowledge about green retrofitting by *I*_*pi*_, and are motivated to participate by external factors such as the level of enterprises technology, and are directly converted to *I*_*pi*_ at a rate of *lE*_4_*I*_*ai*_.

### 3.2 Evolutionary game model

#### 3.2.1 Basic assumptions

Assumption 1: The main participants in the green retrofitting of traditional residential buildings, the government, enterprises and residents, are all rational individuals.

Assumption 2: The main participants all have two strategic choices. The strategy of the government is set to incentivize or not to incentivize, the strategy of enterprises is set to invest or not to invest, and the strategy of residents is set to participate or not to participate. It is assumed that, at the initial stage of the game, the probability of the government choosing to incentivize is *x* and the probability of choosing not to incentivize is 1−*x*; The probability of the enterprises choosing to invest is *y* and the probability of choosing not to invest is 1−*y*; The probability of the residents choosing to participate is *z* and the probability of choosing not to participate is 1−*z*.

Assumption 3: The government’s incentives, when both enterprises and residents are active, refer to the special subsidies *mB*_1_ given to enterprises and the additional subsidies *nB*_2_ given to participating residents. The government takes the cost *uC*_2_ to actively regulate the whole process of green retrofitting. When enterprises do not invest, the government will give spiritual rewards *J* to participating residents.

Assumption 4: For each additional participating resident, the unit change of comprehensive benefits to the government is *e*, the unit change of extra benefits to the enterprises is *g* and the unit change of negative losses to the enterprises is *f*, the unit change of group stress to the residents is *d* and the unit change of mental satisfaction benefits to the residents is *p*. Thus $E_{i}=(\max I_{pi}-I_{0})e$, $G_{i}=(\max I_{pi}-I_{0})g$, $F_{i}=(\max I_{pi}-I_{0})f$, $D_{i}=(\max I_{pi}-I_{0})d$, $P_{i}=(\max I_{pi}-I_{0})p$ (*i* = 1,2). Where *i* = 1 represents the tripartite benefits when the government chooses to incentivize and *i* = 2 represents the tripartite benefits when the government chooses not to incentivize.

Based on the above assumptions, the game payoffs matrix for the government, enterprises and residents is obtained, as shown in Table 2.

**Table 2:** Payoffs matrix for green retrofitting of traditional residential buildings.

Investment retrofitting enterprises	Government incentive *x*		Government no incentive 1−*x*	
	Residents participate *z*	Residents do not participate 1−*z*	Residents participate *z*	Residents do not participate 1−*z*
Invest *y*	$E_{1}-qC_{1}-mB_{1}-nB_{2}-uC_{2}+W$	−*qC*_1_+*W*	*E*_2_−*qC*_1_	−*qC*_1_
	$lE_{3}-C_{3}+mB_{1}+G_{1}$	−*C*_4_	*lE*_3_−*C*_3_+*G*_2_	−*C*_4_
	$E_{4}-kC_{5}+nB_{2}+P_{1}$	−*D*_1_	*E*_4_−*kC*_5_+*P*_2_	−*D*_2_
Not invest 1−*y*	−*qC*_1_−*J*+*W*	−*qC*_1_+*W*	−*qC*_1_	−*qC*_1_
	−*F*_1_	0	−*F*_2_	0
	*J*	−*D*_1_	0	−*D*_2_

## 4 Stability analysis of the evolutionary game

### 4.1 Construction of the replicated dynamic equation

The expected benefit of the government choosing the incentive strategy is denoted as *U*_11_, the expected benefit of the government choosing the no incentive strategy is denoted as *U*_12_.

The expected benefit equation of choosing the incentive strategy by the government is:

$$\begin{array}{l} U_{11}=yz(E_{1}-qC_{1}-mB_{1}-nB_{2}-uC_{2}+W)+y(1-z)(-qC_{1}+W)+(1-y)z(-qC_{1}-J+W)\\ \;+(1-y)(1-z)(-qC_{1}+W) \end{array}$$ (2)

The expected benefit equation for choosing the no incentive strategy for government is:

$$U_{12}=yz(E_{2}-qC_{1})+y(1-z)(-qC_{1})+(1-y)z(-qC_{1})+(1-y)(1-z)(-qC_{1})$$ (3)

The replicated dynamic equation for choosing the incentive strategy by the government is:

$$F(x)=x(1-x)(U_{11}-U_{12})=x(1-x)[yz(E_{1}-mB_{1}-nB_{2}-uC_{2}-E_{2}+J)+z(-J)+W]$$ (4)

Similarly, the replicated dynamic equation for choosing the investment strategy by enterprises is:

$$\begin{array}{l} F(y)=y(1-y)(U_{21}-U_{22})\\ \;=y(1-y)[xz(mB_{1}+G_{1}+F_{1}-G_{2}-F_{2})+z(lE_{3}-C_{3}+G_{2}+F_{2}+C_{4})-C_{4}] \end{array}$$ (5)

The replicated dynamic equation for choosing the participation strategy by residents is:

$$\begin{array}{l} F(z)=z(1-z)(U_{31}-U_{32})\\ \;=z(1-z)[xy(nB_{2}+P_{1}-J-P_{2})+x(J+D_{1}-D_{2})+y(E_{4}-kC_{5}+P_{2})+D_{2}] \end{array}$$ (6)

### 4.2 The analysis on evolutionary stability point

Let *F*(*x*) = 0, *F*(*y*) = 0, *F*(*z*) = 0. It can be obtained the nine equilibrium points of the tripartite evolutionary game model, and the stability of the equilibrium points of the mixed strategy will not be considered in this paper [87]. The local equilibrium point is not certainly the evolutionary stability point of the system, and the system evolutionary stability strategy can be determined by the Jacobi matrix proposed by Friedman [88]. By finding the Jacobi matrix of the replicated dynamic equation, the eigenvalues corresponding to the eight local equilibrium points are obtained, as shown in Table 3.

**Table 3:** Eigenvalues of equilibrium points.

Equilibrium points	λ_1_	*λ*_2_	*λ*_3_
(0,0,0)	*W*	−*C*_4_	*D*_2_
(0,0,1)	−*J*+*W*	$lE_{3}-C_{3}+G_{2}+F_{2}$	−*D*_2_
(0,1,0)	*W*	*C*_4_	$E_{4}-kC_{5}+P_{2}+D_{2}$
(0,1,1)	$E_{1}-mB_{1}-nB_{2}-uC_{2}-E_{2}+W$	$-(lE_{3}-C_{3}+G_{2}+F_{2})$	$-(E_{4}-kC_{5}+P_{2}+D_{2})$
(1,0,0)	−*W*	−*C*_4_	*J*+*D*_1_
(1,0,1)	*J*−*W*	$mB_{1}+G_{1}+F_{1}+lE_{3}-C_{3}$	−(*J*+*D*_1_)
(1,1,0)	−*W*	*C*_4_	$nB_{2}+P_{1}+D_{1}+E_{4}-kC_{5}$
(1,1,1)	$-(E_{1}-mB_{1}-nB_{2}-uC_{2}-E_{2}+W)$	$-(mB_{1}+G_{1}+F_{1}+lE_{3}-C_{3})$	$-(nB_{2}+P_{1}+D_{1}+E_{4}-kC_{5})$

As *W*, *D*_2_, *C*_4_, *J* and *D*_1_ are definite positive numbers, (0,0,0), (0,1,0), (1,0,0) and (1,1,0) are all unstable points and are scenarios where residents do not participate in green retrofitting, therefore they are not discussed.

When −*J*+*W*<0 and *lE*_3_−*C*_3_+*G*_2_+*F*_2_<0, which means that the spiritual rewards given to residents when the government incentivizes are greater than the reputation enhancement, and when the government does not incentivize, the cost paid by the enterprises to invest is greater than the loss when they do not invest, and the cost for residents to participate is less than the cost of not participating. At this time only residents choose to participate in green retrofitting. Therefore, the evolutionary stability point is (0,0,1). When *J*−*W*<0 and $mB_{1}+G_{1}+F_{1}+lE_{3}-C_{3}<0$, that is, when the government incentivizes, the reputation enhancement of the government is greater than the spiritual rewards given to the residents by the government and the benefits of investment by the enterprises are less than the benefits of no investment, the evolutionary stability point is (1,0,1). Neither of these stability points is the target stability point for the design of the green retrofit system.

When $E_{1}-mB_{1}-nB_{2}-uC_{2}-E_{2}+W<0$, $lE_{3}-C_{3}+G_{2}+F_{2}>0$ and $E_{4}-kC_{5}+P_{2}+D_{2}>0$, it means that the benefits of government no incentives are greater than the benefits of incentives, and when the government does not incentivize, the benefits of enterprises investing are greater than those of no investment, the benefits of participation by residents are greater than those of no participation, the evolutionary stability point is (0,1,1). The enterprises and residents form a win-win cooperation situation, which achieves the ideal stable state without government incentives.

When $E_{1}-mB_{1}-nB_{2}-uC_{2}-E_{2}+W>0$, $mB_{1}+G_{1}+F_{1}+lE_{3}-C_{3}>0$ and $nB_{2}+P_{1}+D_{1}+E_{4}-kC_{5}>0$, namely, the benefits of government incentives are greater than the benefits of no incentives, and the benefits of enterprise investment are greater than the benefits of no investment and the benefits of resident participation are greater than the benefits of no participation when the government incentivizes, the evolutionary stability point is (1,1,1). The optimal equilibrium with government incentives is reached at this point.

Next, the numerical simulation of the coupled system of dissemination and evolutionary game models is used to analyze the effect of different parameters on the choice of tripartite strategy and the stability of the game.

## 5 Numerical simulation analysis

The total number of residents *N* = 1000 and the number of residents in each of the four states at the initial moment is *S*_0_ = 998, *I*_*p*0_ = 1, *I*_*a*0_ = 1 and *R*_0_ = 0. Refer to [54, 56, 73], the remaining parameter values are set as follows: *α* = 3.25, *β*_1_ = 0.3, *β* = 0.2, *C*_2_ = 0.34, *C*_3_ = 0.6, *C*_4_ = 0.05, *C*_5_ = 1, *B*_1_ = 0.1, *B*_2_ = 0.2, *E*_3_ = 1, *E*_4_ = 0.6, *W* = 0.3, *J* = 0.02, *m* = 0.5, *n* = 0.5, *k* = 0.4, *b* = 0.9, *q* = 0.4, *u* = 0.5, *o* = 0.5, *h* = 0.4, *l* = 0.4, *e* = 0.0004, *g* = 0.0002, *f* = 0.00035, *d* = 0.0001 and *p* = 0.0001.

### 5.1 The impact of social climate level on tripartite strategic choice

**Fig 3:**
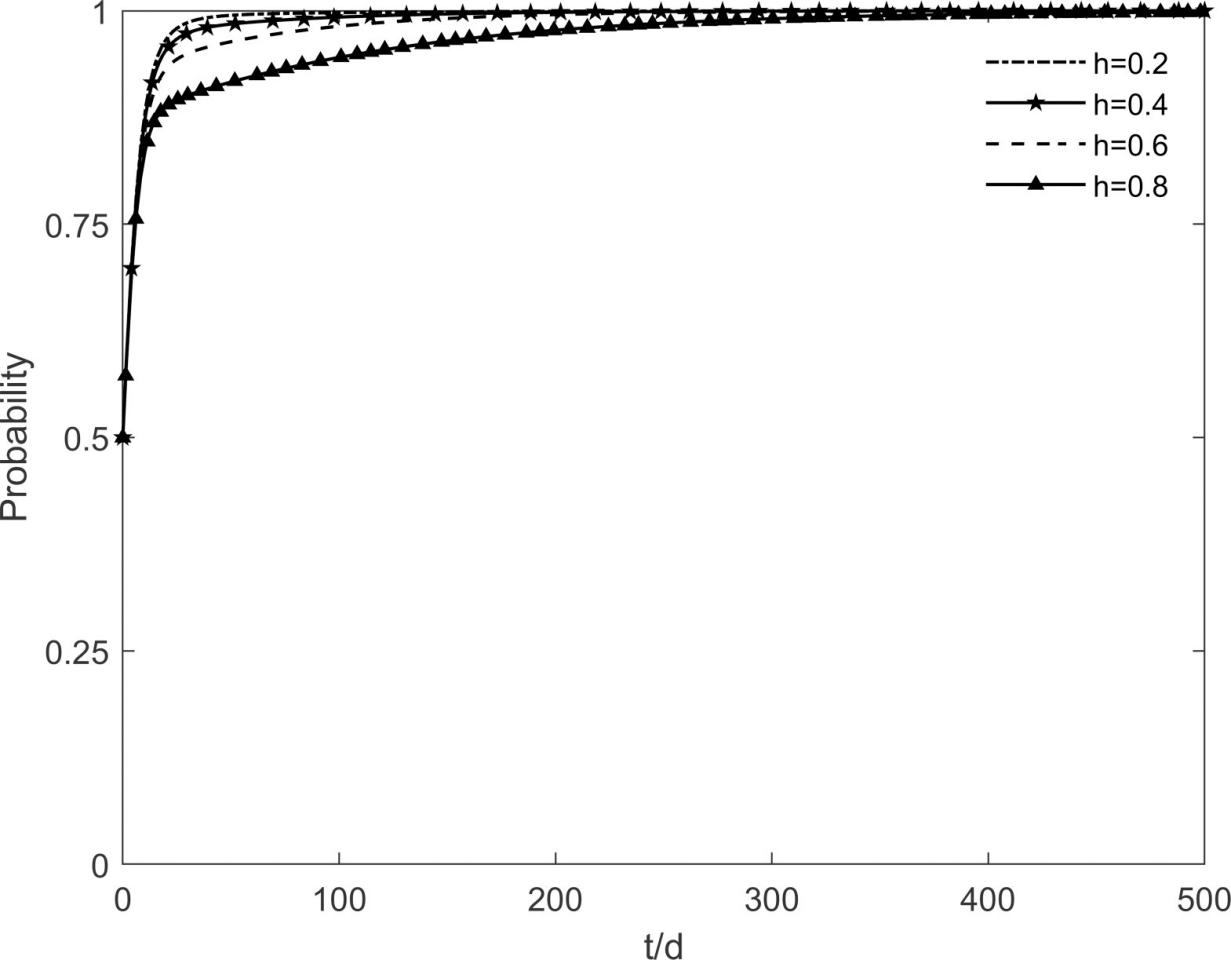
The impact of *h* on the government’s strategic choices.

**Fig 4:**
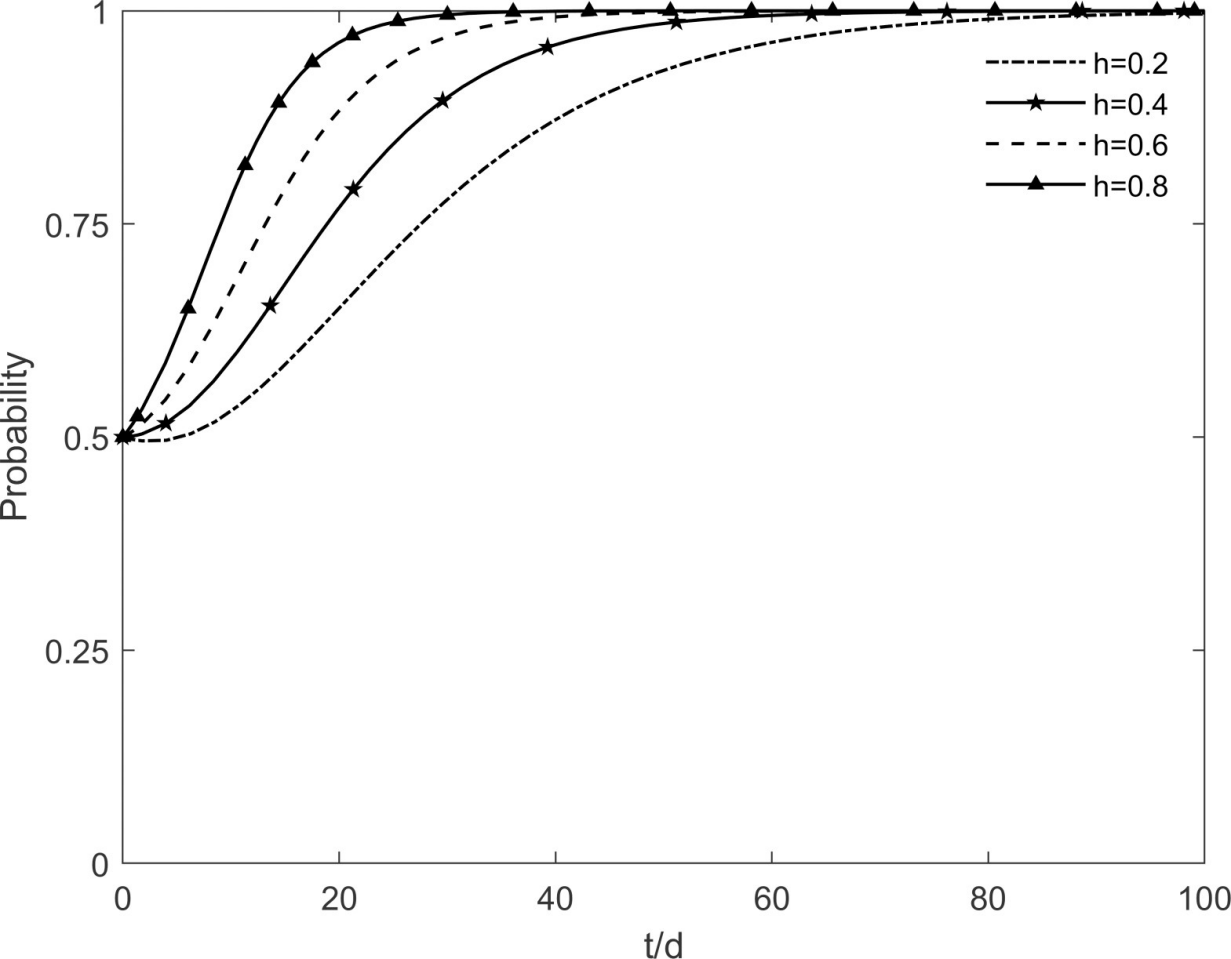
The impact of *h* on the investment retrofitting enterprises’ strategic choices.

**Fig 5:**
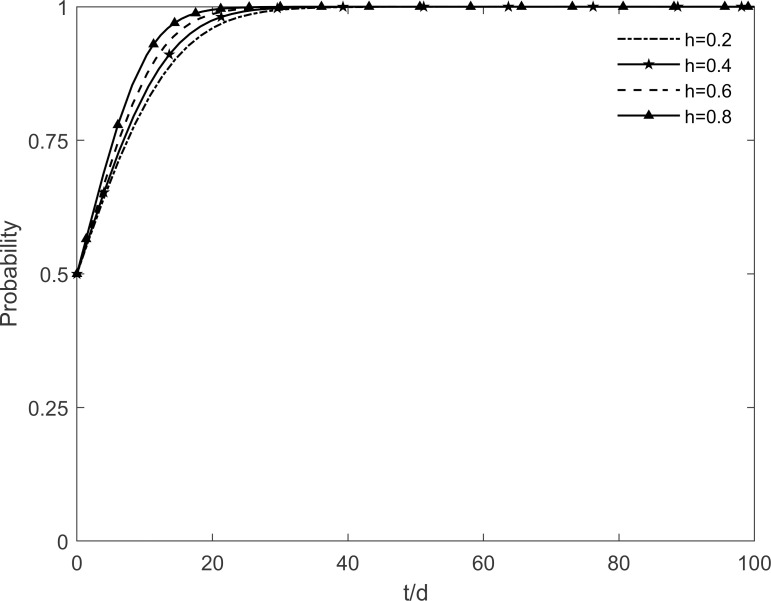
The impact of *h* on residents’ strategic choices.

The social climate level *h* is taken as 0.2, 0.4, 0.6 and 0.8, respectively, and the evolutionary trajectory of tripartite is simulated as shown in Figs 3–5. It can be seen that, when *h* = 0.2, enterprises and residents are slow to reach evolutionary steady state. In contrast, as the level of social climate improves, the rate of conversion of potential and participating residents to immune residents is prolonged and the rate of conversion of resistant residents to immune residents is shortened. It results in a faster rate of "invest and participate" for enterprises and residents, and a relatively longer time for the government to choose the incentive strategy. It indicates that the level of social climate has an important influence on residents’ green retrofitting decisions. Residents have a strong common cognition of the government’s green retrofitting initiatives and have positive enthusiasm for retrofitting in a strong social climate. It also indicates that the number of participating residents effectively influences the investment enthusiasm of enterprises. The higher the level of social climate, the greater the maximum value of participating residents, which motivates market demand-oriented enterprises to invest and retrofit.

### 5.2 The impact of the level of government regulation on tripartite strategic choice

**Fig 6:**
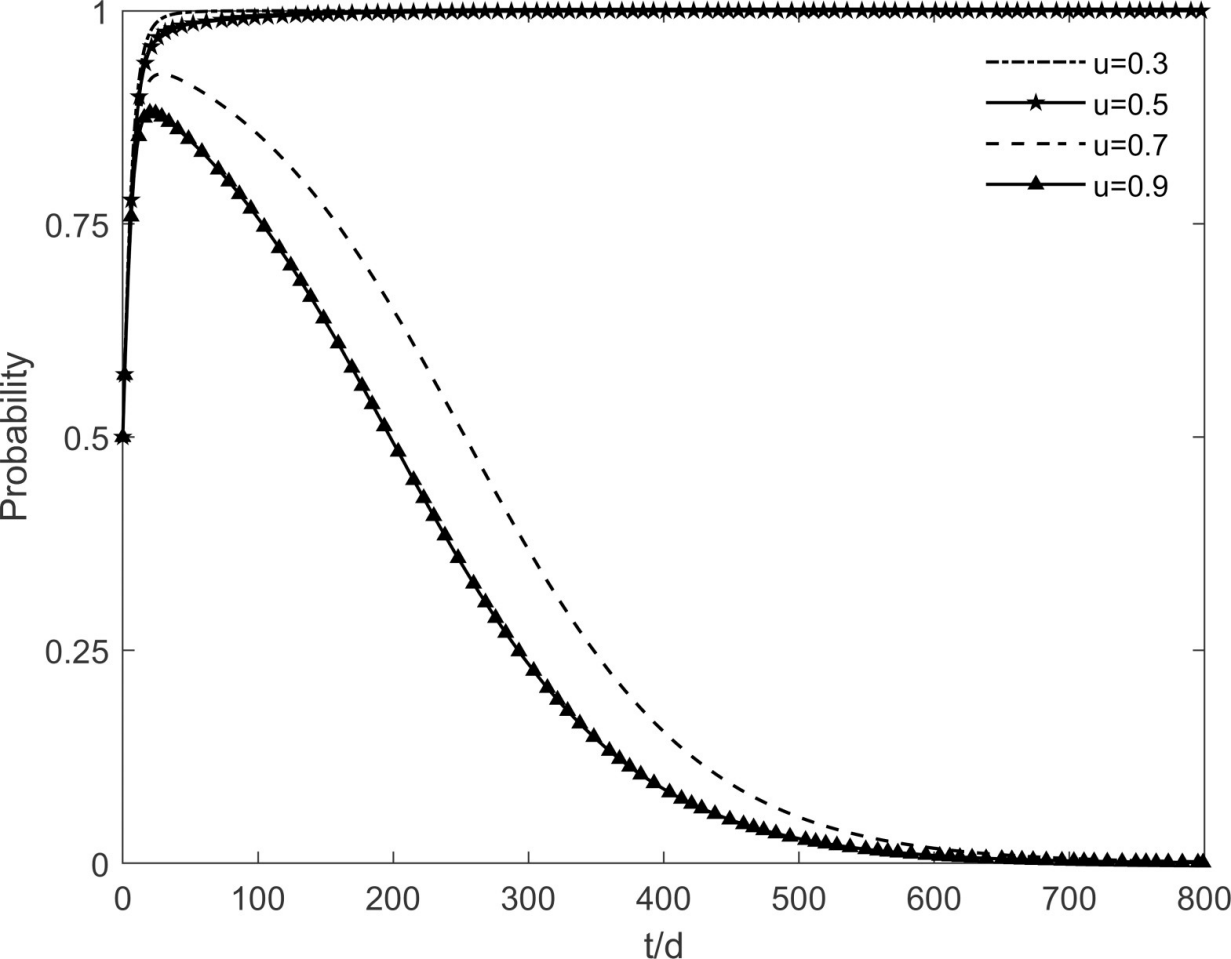
The impact of *u* on the government’s strategic choices.

**Fig 7:**
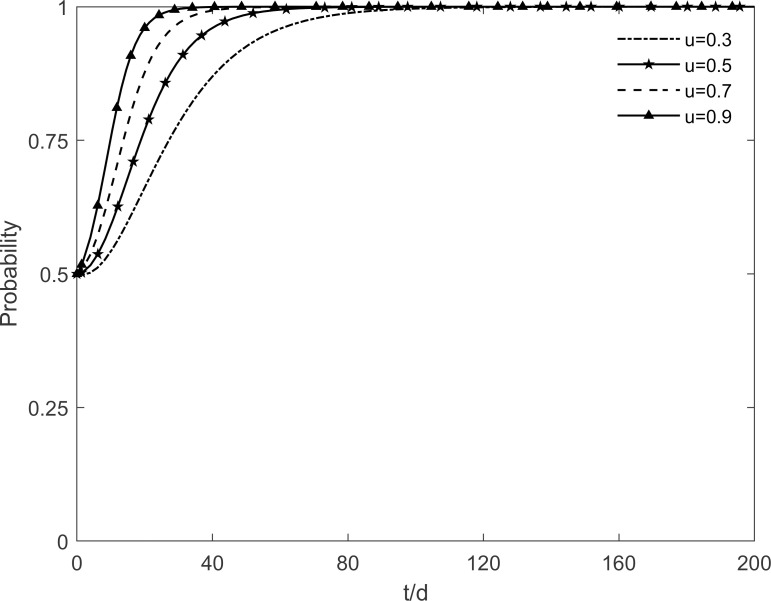
The impact of *u* on the investment retrofitting enterprises’ strategic choices.

**Fig 8:**
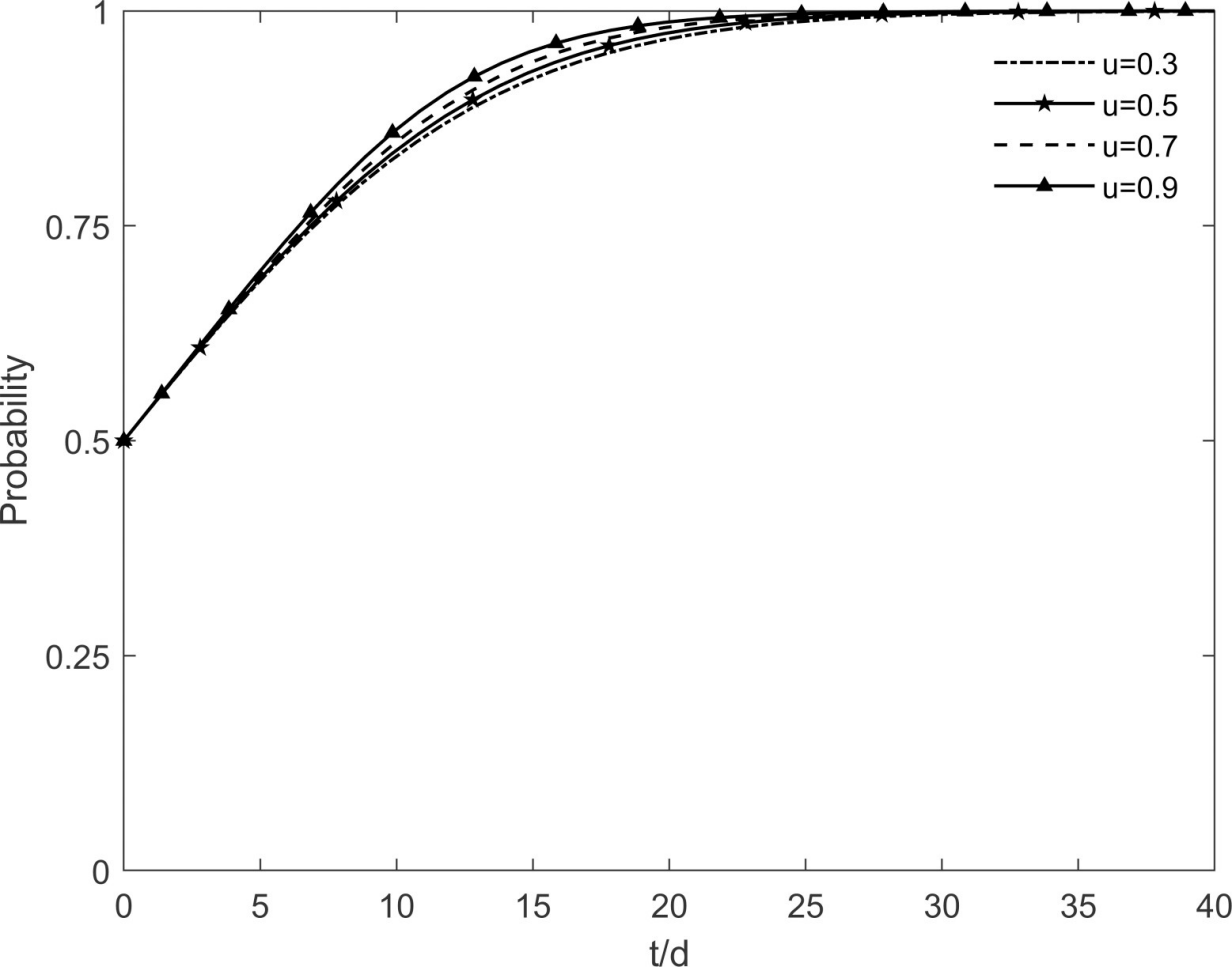
The impact of *u* on residents’ strategic choices.

The strength of government regulation *u* is set to 0.3, 0.5, 0.7 and 0.9 respectively, and the evolutionary trajectory of the tripartite is obtained as shown in Figs 6–8. It can be seen that the strength of government regulation has different effects on the choice of evolutionary strategies of the tripartite. The lack of supervision technology and resources on green retrofitting at the early stage, coupled with the government’s negligent regulation, hinder residents’ enthusiasm to participate in green retrofitting. With the gradual strengthening of government regulation, which reduces residents’ worries and anxiety, the conversion rate of participating residents to immune residents slows down, and enterprises and residents are driven by benefits to accelerate the tendency to actively participate in the strategy. However, when *u*≥0.7, the government chooses the no incentive strategy as the benefits gained cannot offset expenses. This shows that the government is more susceptible to changes in the strength of regulation because it is directly related to his profits.

### 5.3 The impact of the level of government publicity and education on tripartite strategic choice

**Fig 9:**
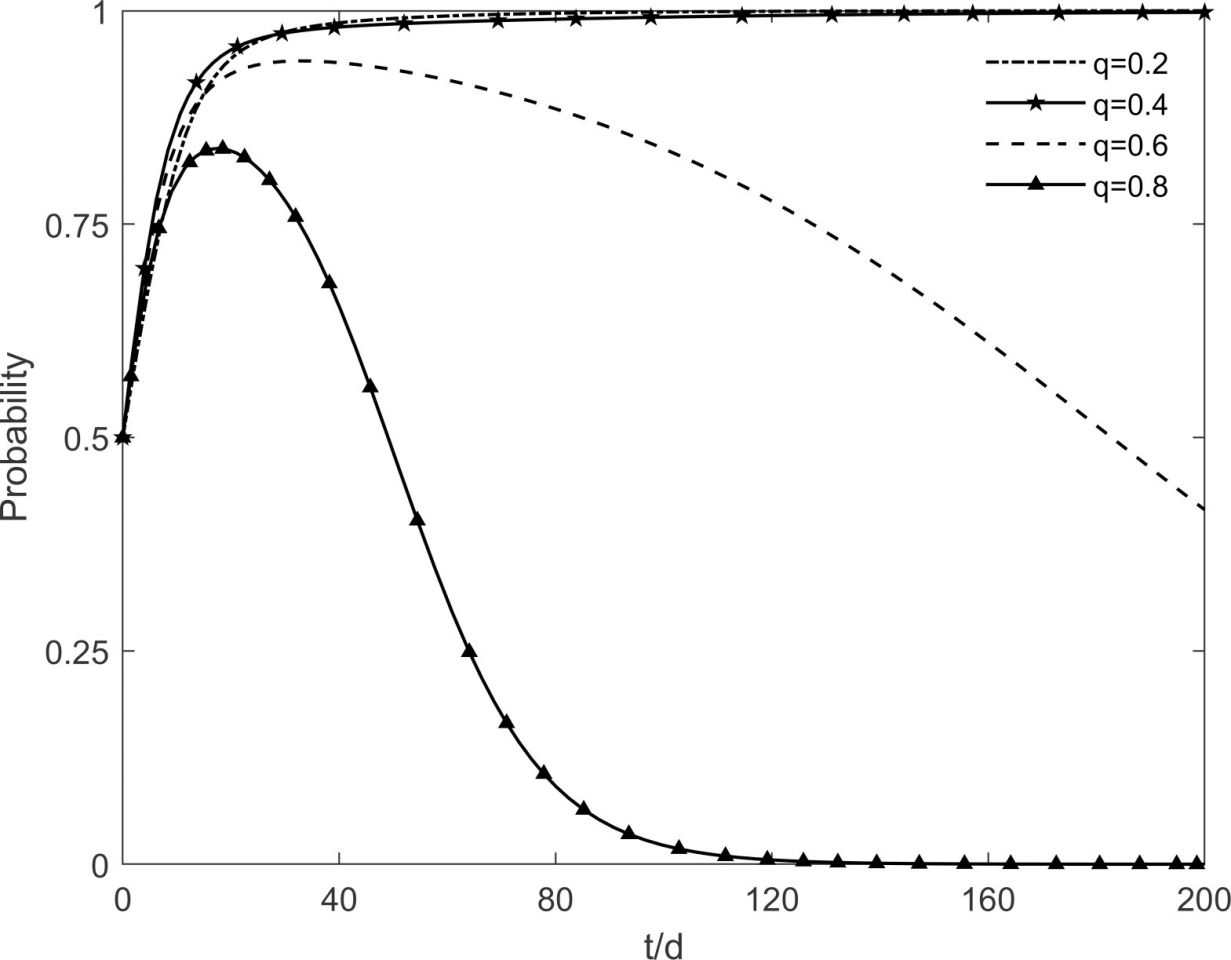
The impact of *q* on the government’s strategic choices.

**Fig 10:**
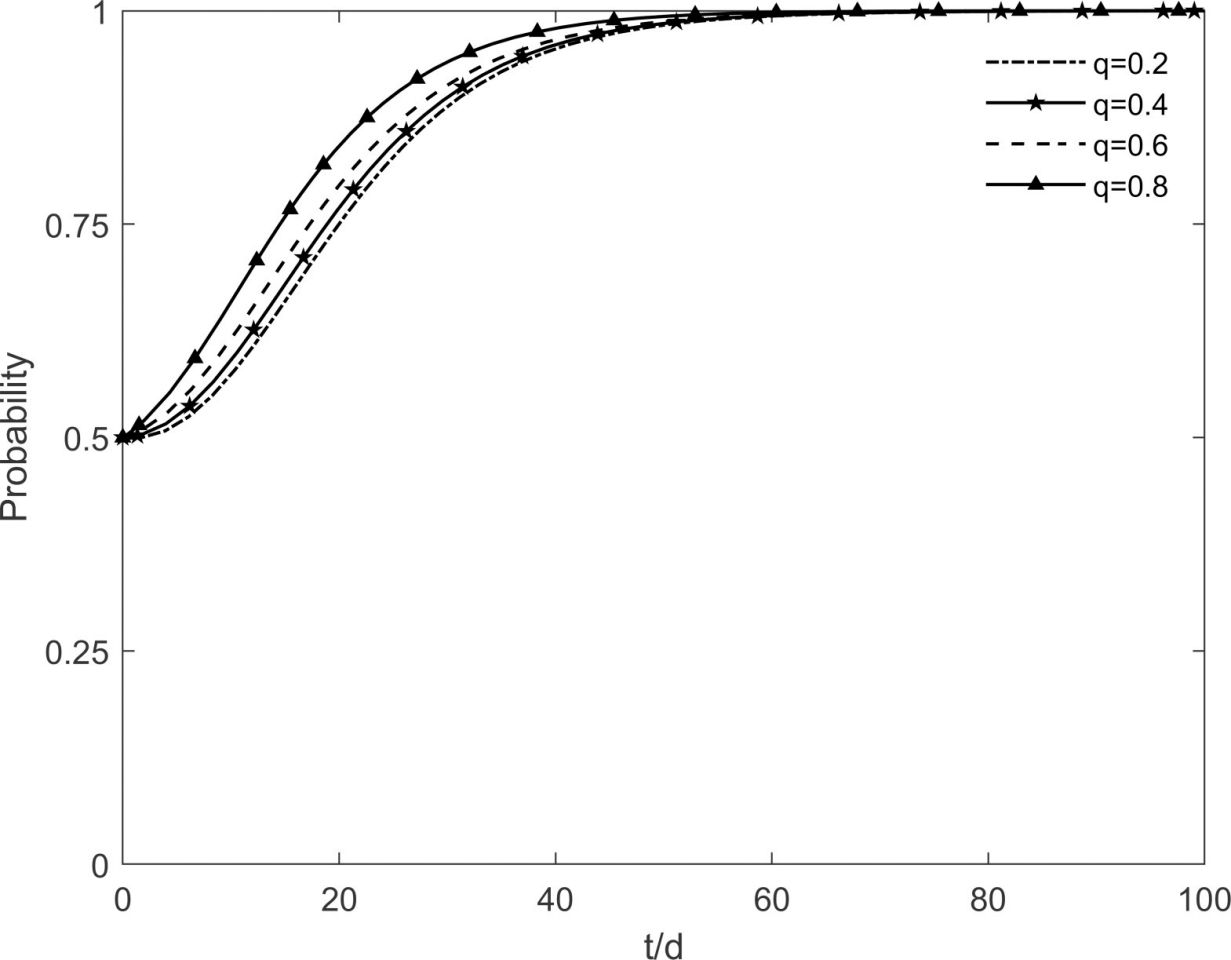
The impact of *q* on the investment retrofitting enterprises’ strategic choices.

**Fig 11:**
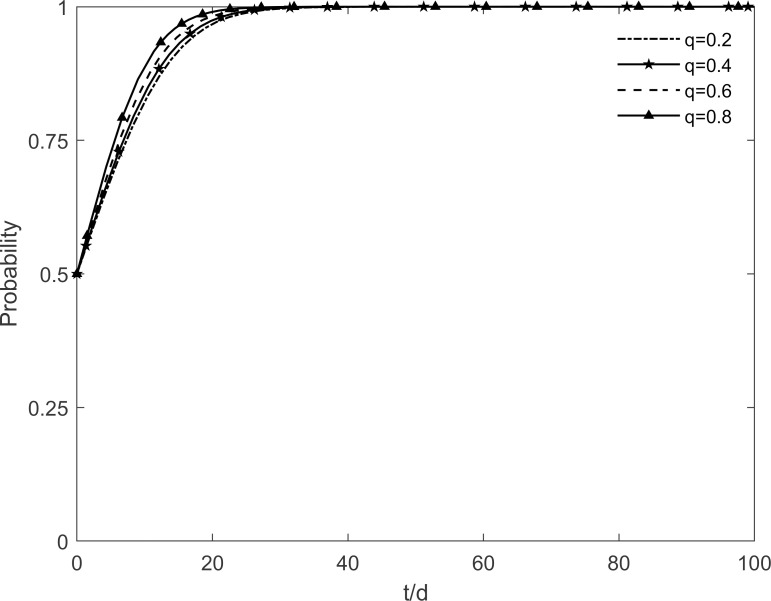
The impact of *q* on residents’ strategic choices.

The process of the change of the stability strategy of each participant is shown in Figs 9–11 when the level of government publicity and education *q* is set to 0.2, 0.4, 0.6 and 0.8 respectively. As can be seen from the figure, the government, enterprises and residents finally reach the stable state of "no incentive, investment, participation" when *q*≥0.6. It is because the lack of knowledge of green retrofitting among residents at the initial moment, which reduces their motivation to participate in green retrofitting. However, with the improvement of the government’s publicity and education level, the green retrofitting is deeply rooted in residents’ minds, which increases the conversion rate from resistant residents to participating residents and reduces the conversion rate from participating residents to immune residents, so that the residents tend to participate in the green retrofitting faster. The more residents participate, the more the enterprises are motivated to accelerate the evolution in the direction of investment. At this time, in order to avoid additional costs, the government’s intention to incentivize is weakened and gradually evolves in the direction of no incentives. It can be seen that the level of government publicity and education plays an important role in fostering cooperation between residents and enterprises.

### 5.4 The impact of the intensity of government subsidies to residents on tripartite strategic choice

**Fig 12:**
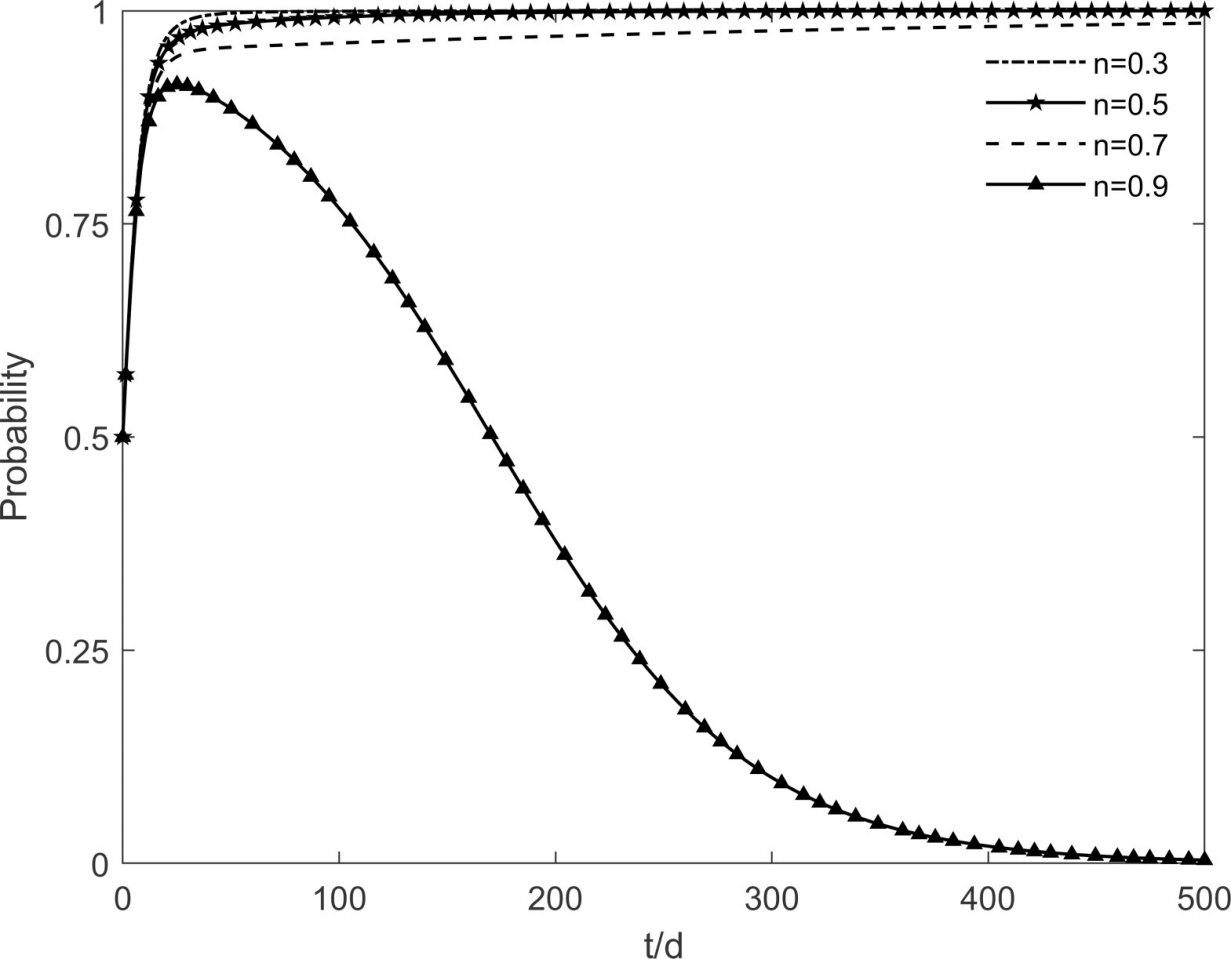
The impact of *n* on the government’s strategic choices.

**Fig 13:**
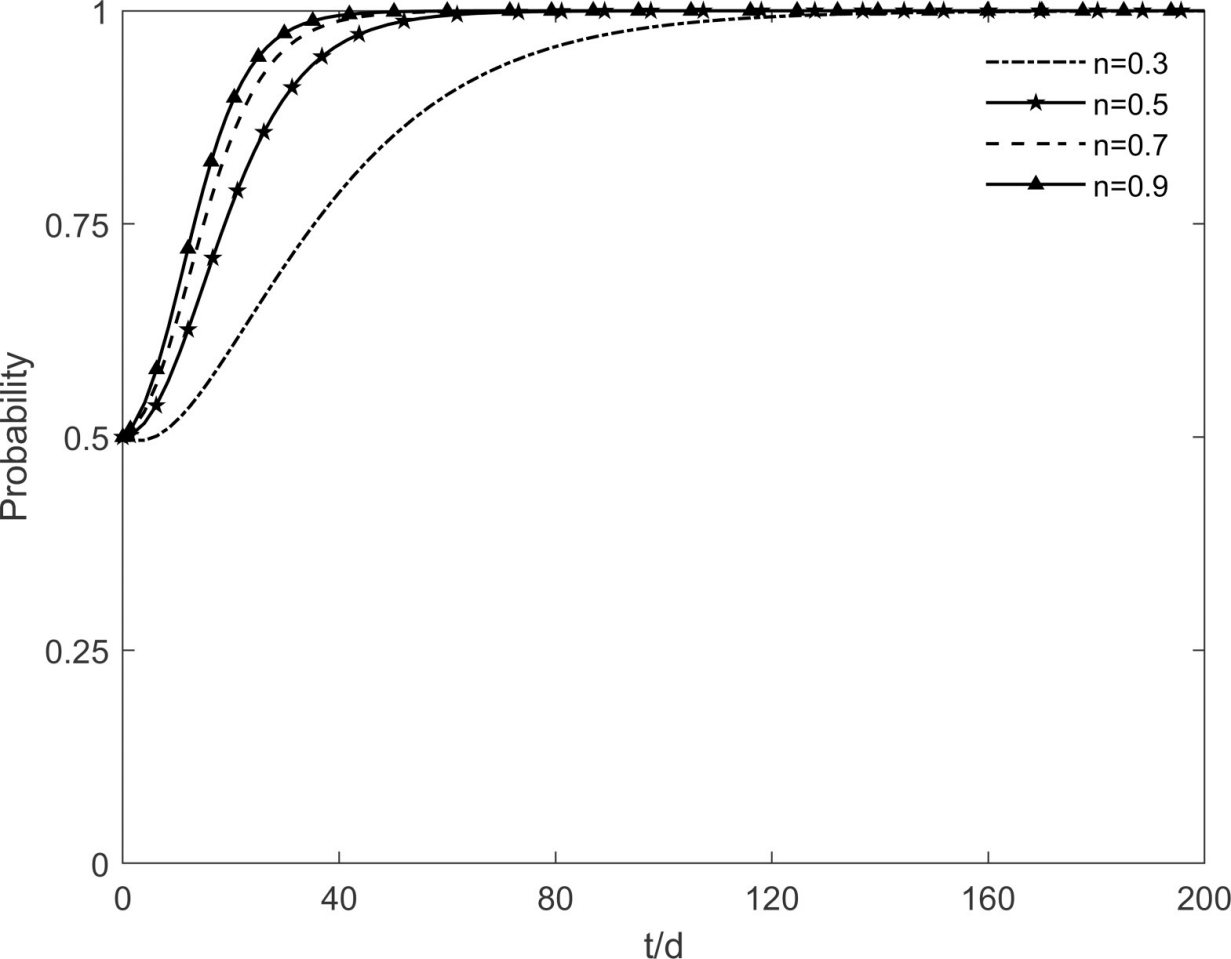
The impact of *n* on the investment retrofitting enterprises’ strategic choices.

**Fig 14:**
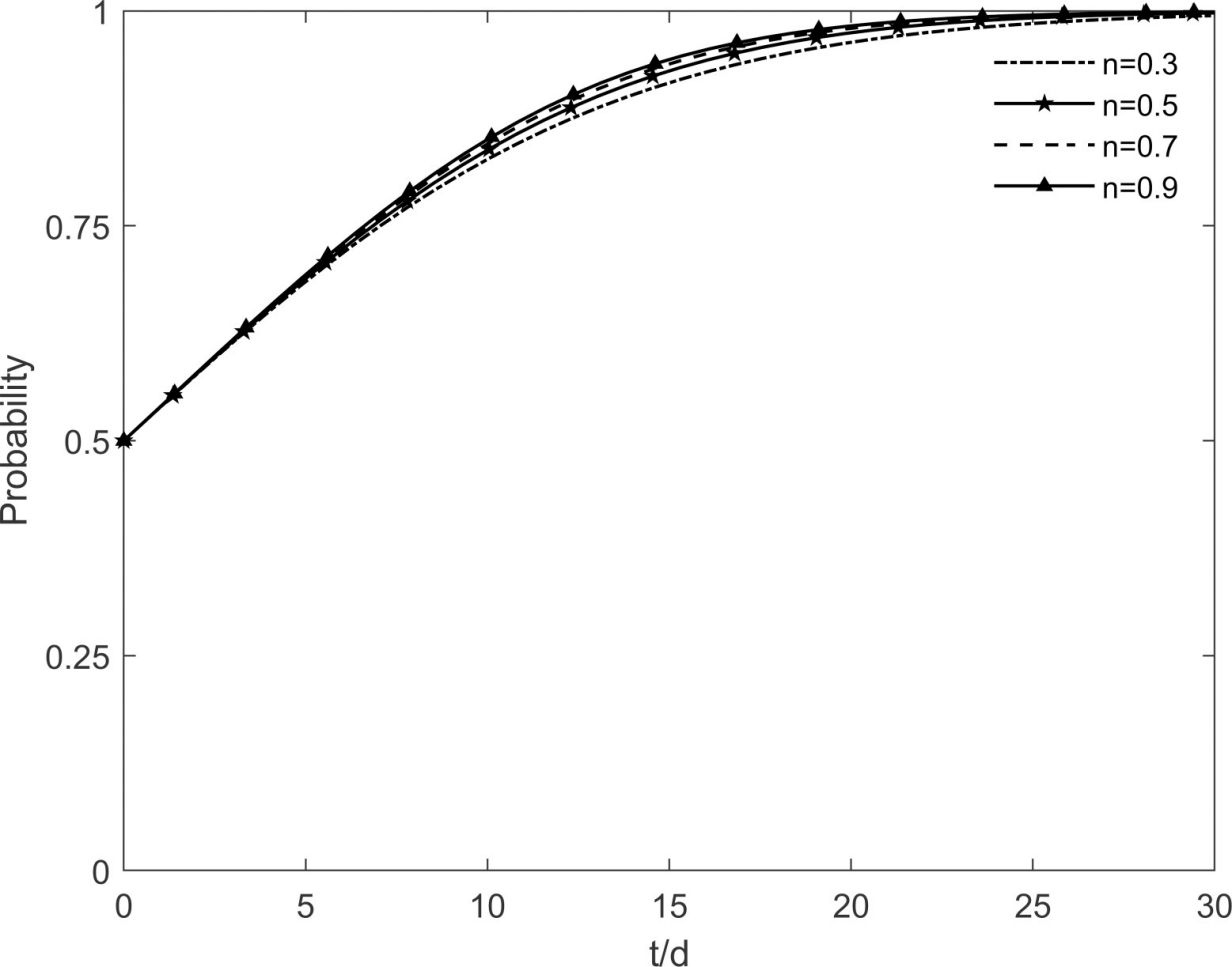
The impact of *n* on residents’ strategic choices.

The evolutionary trajectory of each participant is shown in Figs 12–14 when *n* =0.3, 0.5, 0.7 and 0.9. When *n*<0.9, the government, enterprises and residents reach a stable state of "incentive, investment and participation", which is due to the fact that the rate of conversion of immune residents to participating residents accelerates with the increase of *n*. At this time, the benefits of government’s choice of incentive are greater than the benefits of no incentive, the benefits of enterprises’ investment are greater than the benefits of no investment, and the benefits of residents’ participation are greater than the benefits of no participation. However, when *n* = 0.9, the government’s intention to choose the incentive strategy changes in the face of real financial pressure, and it eventually chooses the no incentive strategy. It can be seen that subsidies of different intensities lead to different evolutionary trajectories of the system. Excessive subsidies not only increase the financial pressure on the government but also cause market disruptions. The government should be concerned about the impact of the subsidy mechanism on itself. This suggests that excessive subsidies are undesirable to promote green retrofit projects of traditional residential buildings in the long run.

### 5.5 The impact of technology level of the enterprises on tripartite strategic choice

**Fig 15:**
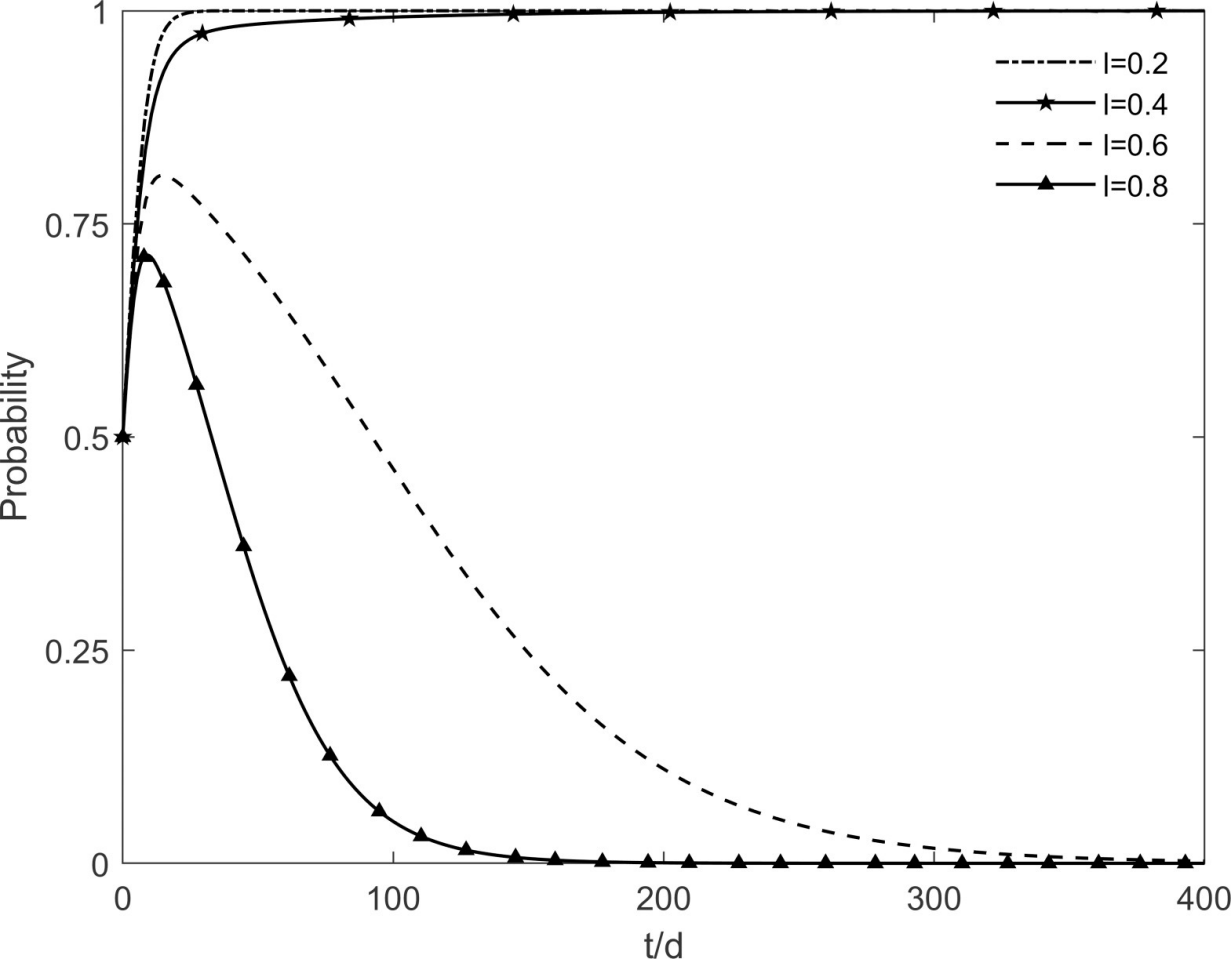
The impact of *l* on the government’s strategic choices.

**Fig 16:**
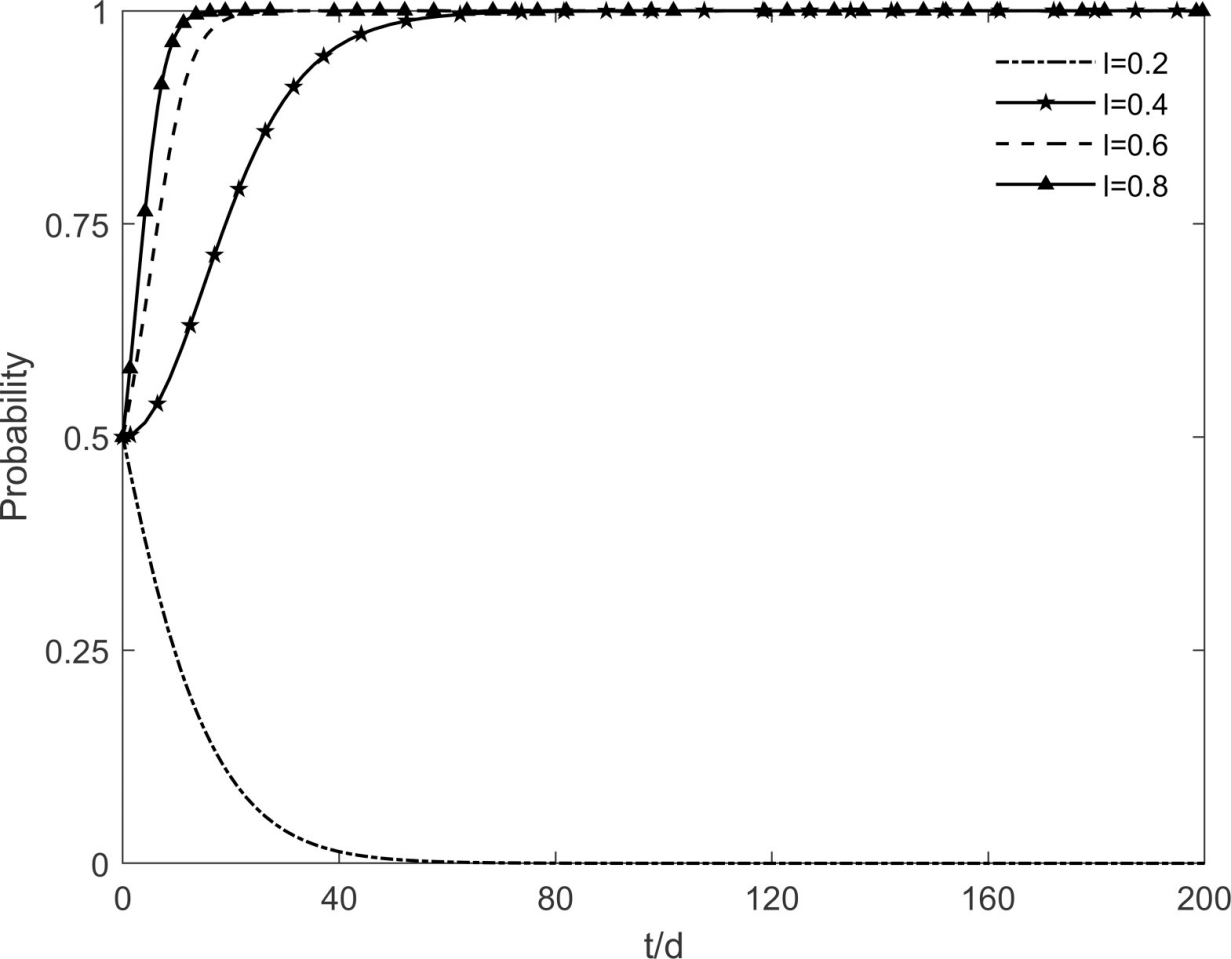
The impact of *l* on the investment retrofitting enterprises’ strategic choices.

**Fig 17:**
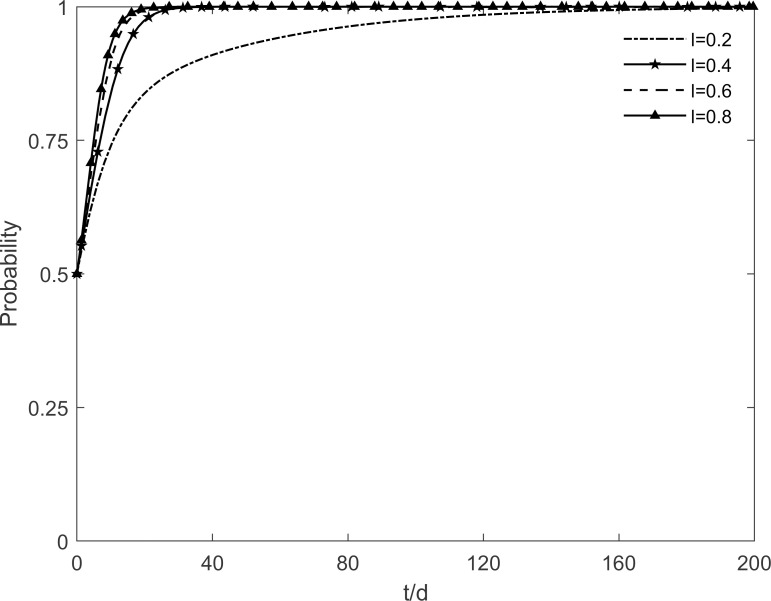
The impact of *l* on residents’ strategic choices.

When the technology level of the enterprise *l* takes the values of 0.2, 0.4, 0.6 and 0.8 respectively, the evolutionary stability strategy change of each participant is obtained as shown in Figs 15–17. It can be found that the technology level of the enterprises has a greater influence on the choice of tripartite strategy. Enhancing the technology level of enterprises, the government evolves from incentive strategy to no incentive strategy, enterprises evolve from no investment strategy to investment strategy, and residents tend to participate in the strategy at a faster rate. It can be seen that with the continuous promotion of green retrofitting, enterprises strive to enhance their technological level which decreases the redundant costs of their investment projects and increase significantly the benefits obtained, and enterprises actively evolve towards the investment state. At the same time, only by improving the technology level of the enterprises will the residents’ participation in the green retrofitting project be fundamentally guaranteed, so that more residents will be converted to participating residents. And as time goes on, enterprises and residents will no longer depend on government incentives, and the government will slowly move towards the no incentive strategy, which will achieve the steady state that is desired in the long term. This finding suggests that the improvement of the technology level of enterprises is necessary to maintain the stability and longevity of the green retrofit market. In other words, the technology level of enterprises directly stimulates market demand. By improving the technology level of enterprises, they can not only obtain high benefits but also attract residents to actively participate in retrofitting.

### 5.6 The impact of residents’ risk perception level on tripartite strategic choice

**Fig 18:**
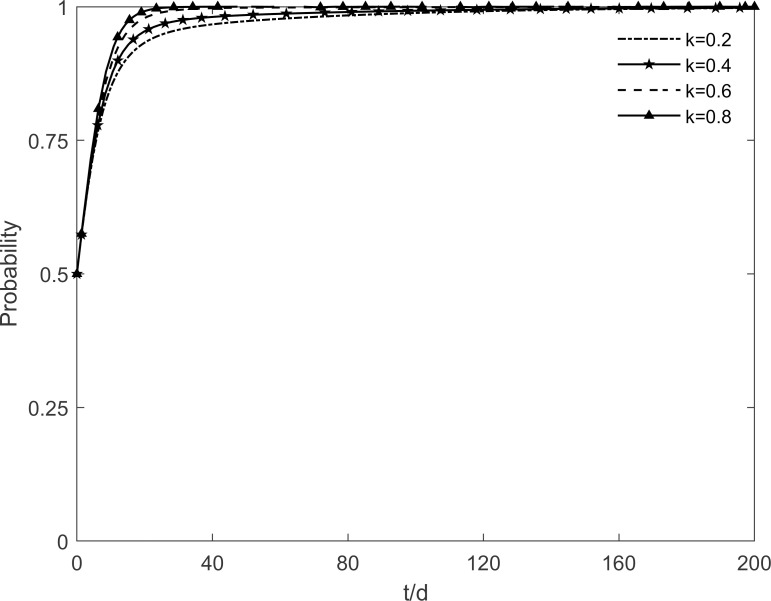
The impact of *k* on the government’s strategic choices.

**Fig 19:**
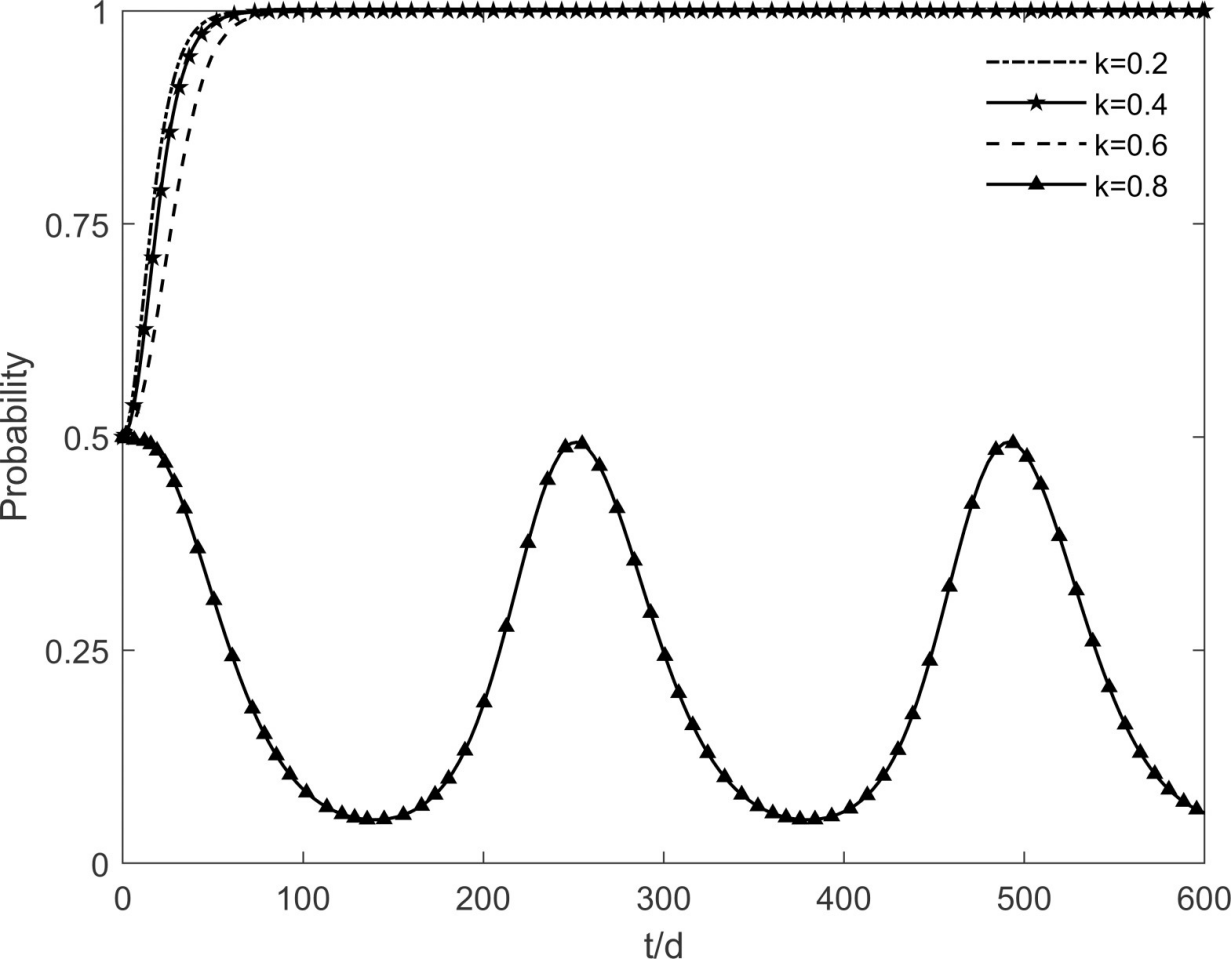
The impact of *k* on the investment retrofitting enterprises’ strategic choices.

**Fig 20:**
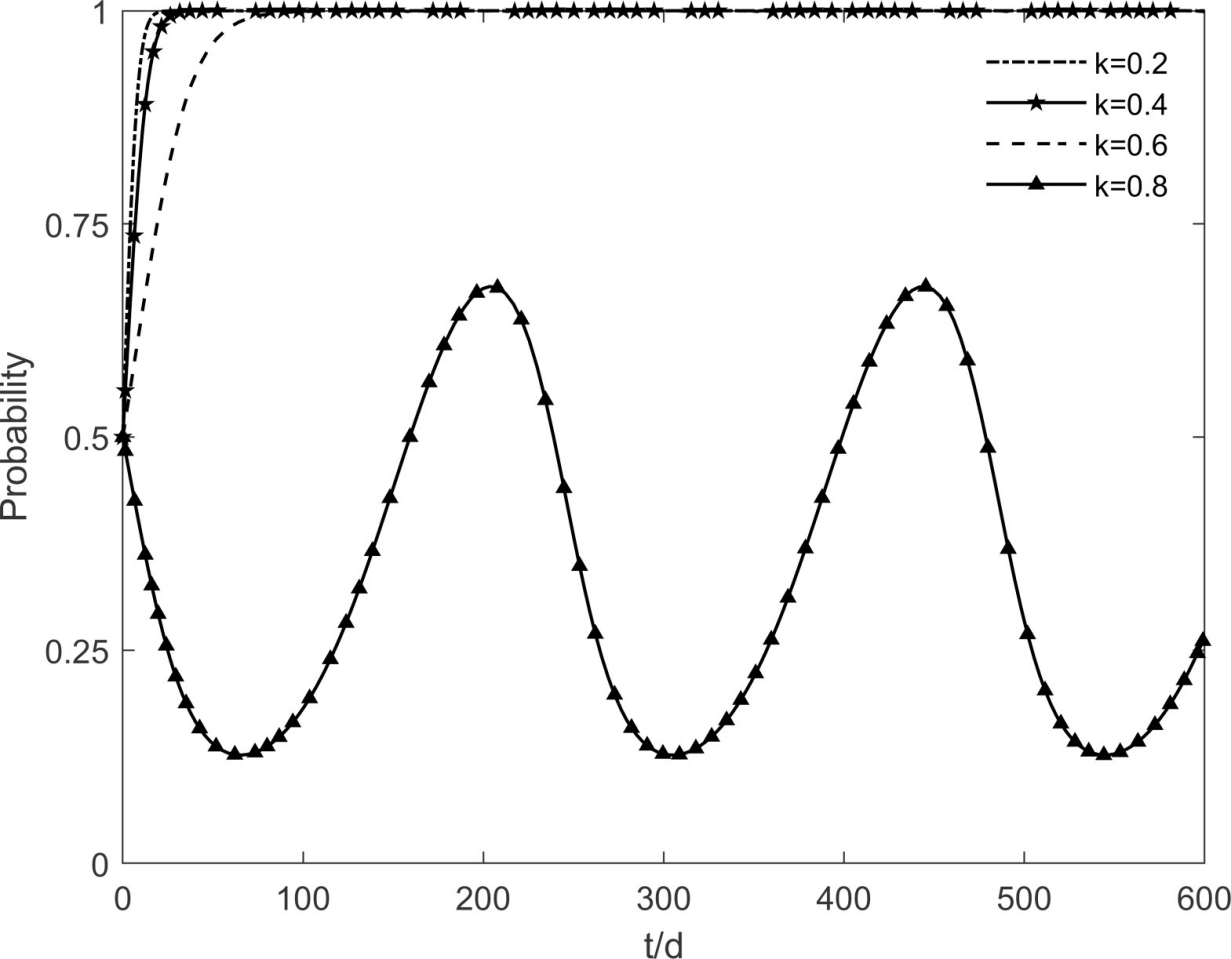
The impact of *k* on residents’ strategic choices.

The residents’ risk perception level *k* is taken as values 0.2, 0.4, 0.6 and 0.8 respectively, and the evolutionary trajectory of each participant is observed as shown in Figs 18–20. As shown in the figure, changes in the residents’ risk perception level have less impact on the government’s strategic choice, but the evolutionary paths of the enterprises and residents show cyclical oscillations with increasing risk perception levels. It is because participating and immune residents are susceptible to misperceptions of green retrofitting by resistant residents in their social interactions. The rate of conversion of participating and immune residents to resistant residents accelerates as the residents’ risk perceptions increase, resulting in a fundamental failure to guarantee the benefits of both enterprises and residents, which prevents enterprises and residents from reaching a stable equilibrium. It shows that the increased risk perception of residents leads to a decrease in the number of participating residents because of the outbreak of negative emotions among the population. At the same time, the lack of market demand is deterring the enthusiasm of enterprises because their benefit goals cannot be achieved. Thus, it is necessary to minimize the residents’ risk perception and improve their judgment and rational perception.

### 5.7 The impact of residents’ cognitive benefits on tripartite strategic choice

**Fig 21:**
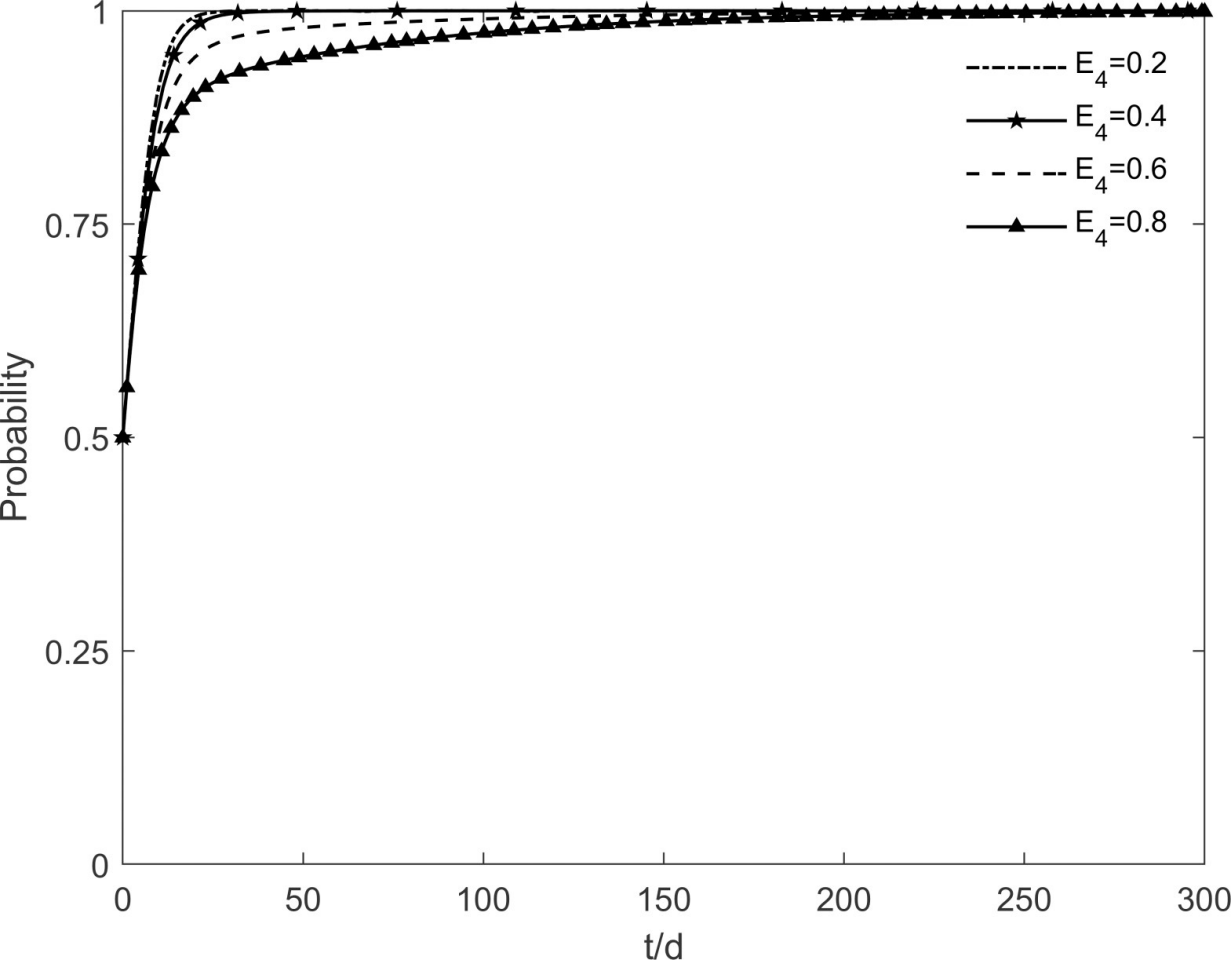
The impact of *E*_4_ on the government’s strategic choices.

**Fig 22:**
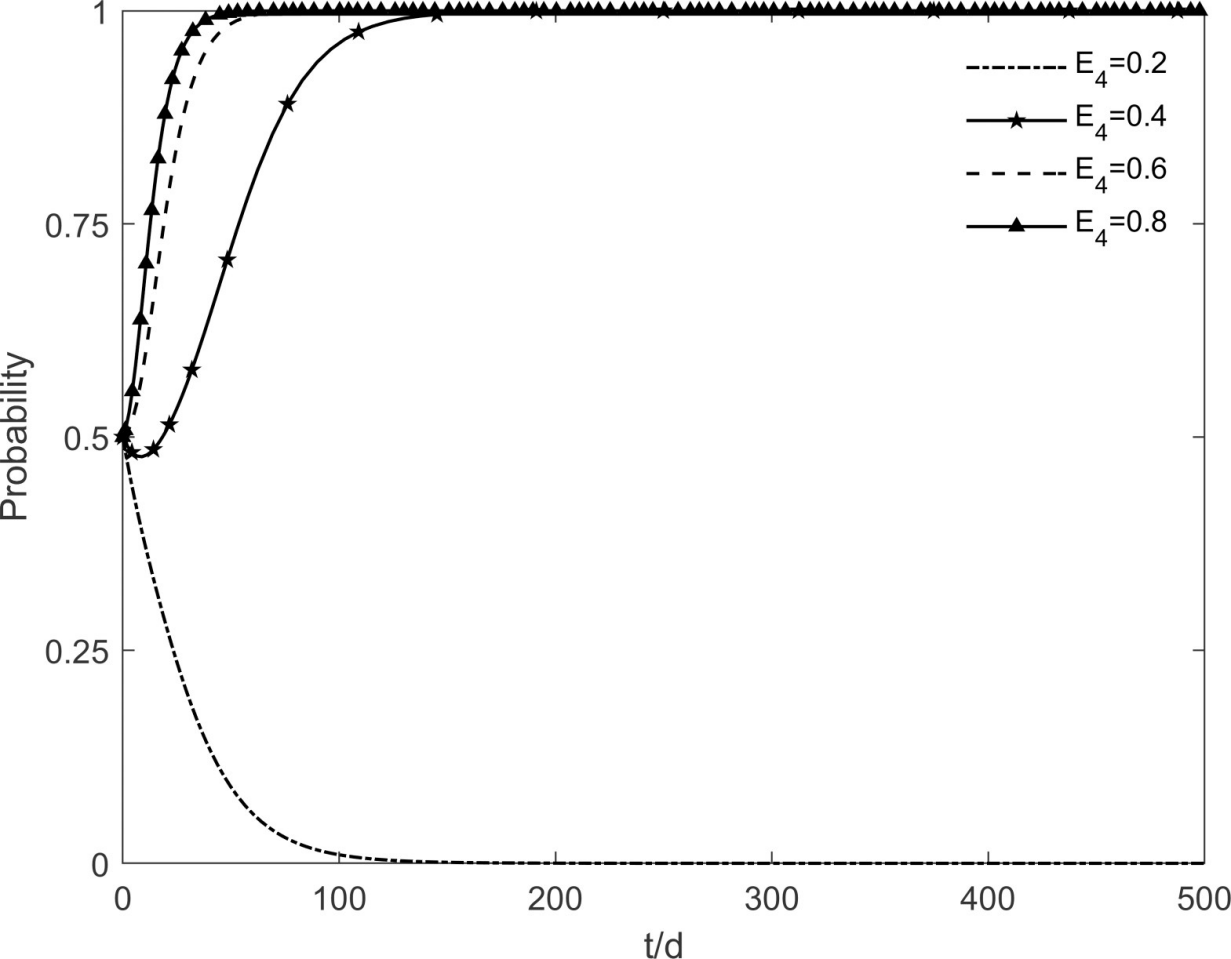
The impact of *E*_4_ on the investment retrofitting enterprises’ strategic choices.

**Fig 23:**
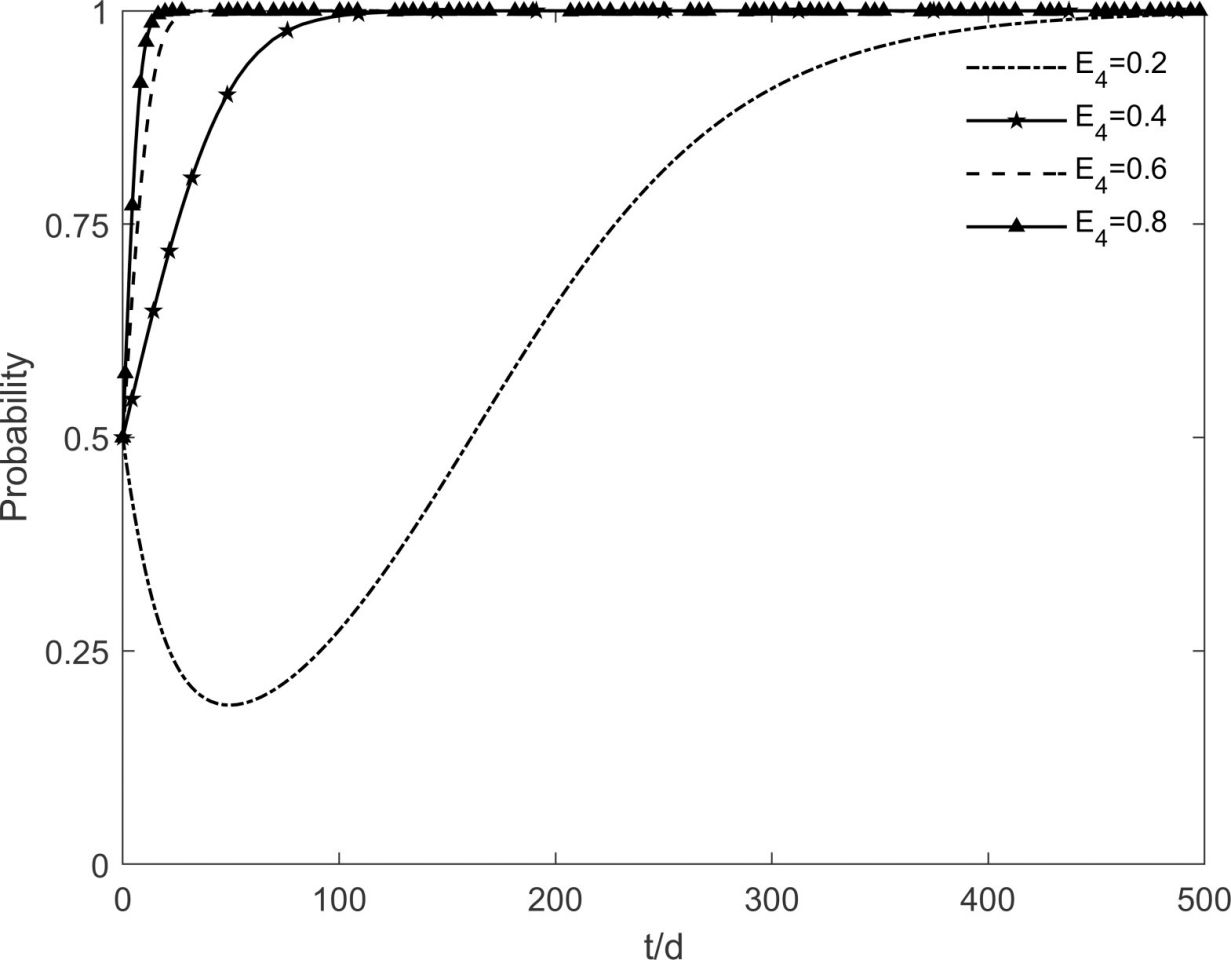
The impact of *E*_4_ on residents’ strategic choices.

The change in the evolutionary stability strategy for each participant is obtained by taking values of 0.2, 0.4, 0.6 and 0.8 for the residents’ cognitive benefits *E*_4_, respectively, as shown in Figs 21–23. The decrease in residents’ cognitive benefits significantly reduces their motivation to participate in retrofitting. Enterprises evolve towards no investment in consideration of the lower benefits of residents’ participation in retrofitting. Conversely, the dissemination of information and knowledge to resistant and immune residents increases the rate at which residents choose to participate, by reducing their perception of uncertainty and increasing cognitive benefits. The rise in the number of participating residents also increases the motivation of enterprises to choose investment and slows down the government’s choice of incentive strategy, ultimately maintaining stability in the "incentive, investment, participation" state. This result shows that the decrease in the residents’ cognitive benefits first has a significant impact on the evolutionary trend of the residents’ intention to green retrofit. Enterprises turn to non-investment due to the decrease in the number of participating residents. It also shows that enterprises are very sensitive to changes in market demand. When the number of participating residents decreases, enterprises are the first to withdraw from the green retrofit market. Thus, it can be believed that increasing the residents’ cognitive benefits is the basis for cooperation between the two parties.

## 6 Conclusions and recommendations

Green retrofitting of traditional residential buildings is a key area of carbon emission reduction in the construction sector. This paper firstly constructs a dissemination model of the green retrofitting intention of residents group, and couples it with the evolutionary game model of the government, enterprises and residents to comprehensively analyze the dissemination of residents’ intention to green retrofitting and the dynamic interaction process of each game party in the green retrofitting process. The impact of relevant parameters on the dissemination and the choice of strategy of the tripartite game are also analyzed through simulation calculations. The results show that:

1. The level of social climate influences the speed which steady state is reached by the government, enterprises and residents. However, its role is ignored in the existing studies [54, 56, 57, 62]. According to the results, when the level of social climate is too low, the government needs to actively incentivize the participation of enterprises and residents. When the level of social climate rises, more residents are converted to participating residents, and the benefits of enterprises and residents increase. Therefore, to promote the sustainable development of the green retrofit market for traditional residential buildings, the government should form a favorable social climate.
2. The strength of government regulation and the intensity of subsidies given to residents can change the government’s strategic choices. However, excessive regulation and subsidies can reduce the benefits of the government and make the government turn to the "no incentive" strategy, which is consistent with the findings of Chen et al. [56] and Wu et al. [57]. Differently, this paper also reveals that the conversion rate of participating residents to immune residents is slowed down and the conversion rate of immune residents to participating residents is accelerated under increased regulation and subsidies, which results in a faster rate of enterprises choosing to invest and residents choosing to participate. Therefore, to ensure the effectiveness of the incentive policy, the government should choose the intensity of regulation and subsidies in a reasonable proportion.
3. The improvement in the level of government publicity and education and the technology level of enterprises has a significant role to play in achieving the desired stable state of no government incentives. Improving the level of publicity and education accelerates the conversion of resistant residents to immune residents and slows down the conversion of participating residents to immune residents; improving the technology level of enterprises significantly increases the size of the group of participating residents, and the ideal stabilization strategy of "no incentive, investment and participation" is finally achieved by the government, enterprises and residents. These results complement the findings of Wu et al. who found that when enterprises and residents behave positively in the market, the government does not regulate the market in order to reduce the financial burden [57].
4. The cognitive benefits and risk perceptions of residents influence enterprises’ strategic choices. Although existing study points out that market size increases the incremental benefits of enterprises, it does not provide an in-depth explanation of the driving mechanisms of market size on the enterprises’ behavior [62]. This paper confirms that the lower the cognitive benefits and the stronger the risk perception of residents, the slower the conversion rate of resistant and immune residents to participating residents and the faster the conversion rate of participating and immune residents to resistant residents, causing enterprises to have more difficulty reaching the investment stability point. It indicates that residents play an important role in the green retrofitting of traditional residential buildings, and that the larger the size of the resident participation group in the retrofitting, the more enthusiastic the enterprises’ investment will be. It also means that the green retrofitting demand of residents on the consumer side is the direct pulling force for the development of the green retrofitting market of traditional residential buildings.

From the above analysis, it is clear that the collective efforts of the government, enterprises and residents are the direct role factors in changing the current development level of the green retrofitting market of traditional residential buildings. In the short term, the government can play a leading role by setting reasonable regulatory efforts and special subsidy funds, correcting residents’ cognitive biases in a timely manner, creating a favorable social climate and establishing positive channels for the dissemination of green retrofitting intentions. It could promote the participation of residents in green retrofitting and increase the benefits of enterprises’ investment. However, the evolutionary path of "incentive, investment, participation", in which enterprises and residents rely heavily on the stimulation of the government’s economic interests, will not only increase the government’s financial pressure but also will lead to serious situations such as governance failure, which is difficult to sustain in the long term. To achieve the ideal long-term state of "no incentive, investment, participation", the government should strive to improve the level of publicity and education, increase the transparency of information, and actively disseminate green retrofitting knowledge to the community; investment retrofitting enterprises should improve their professional skills in green retrofitting, reduce the cost of retrofitting, build their development advantages, and stimulate the interest of residents to participate in retrofitting. In turn, this will stimulate the investment enthusiasm of the enterprises and achieve mutual benefits.

This paper provides a more comprehensive analysis of the dynamics of residents’ intra-group retrofitting intentions and its impact on the equilibrium steady state of the tripartite strategy. However, there are certain limitations: (1) The dissemination of green retrofitting intentions among residents of traditional residential buildings is influenced by multiple factors, and each influencing factor will interact with each other, but the interaction between the influencing factors is not considered in this paper. (2) This study does not consider the differences in the technical characteristics and green retrofitting decision process between traditional residential buildings, which include both traditional urban community residential buildings and traditional rural single-family residential buildings. (3) The model in this paper is focused on analyzing the regularity of the mechanism. In the future, realistic information and data can be obtained through questionnaire and field research to conduct empirical study on the model.

## Supporting information

S1 Fig (ZIP)
