# Supplementary figures and images for: Evolutionary analysis of stakeholder behavior in green retrofitting of traditional residential buildings based on dissemination and game models
Source: PLoS One. 2023 Mar 16;18(3):e0282314. doi: 10.1371/journal.pone.0282314 (PMC10019687; doi:10.1371/journal.pone.0282314)

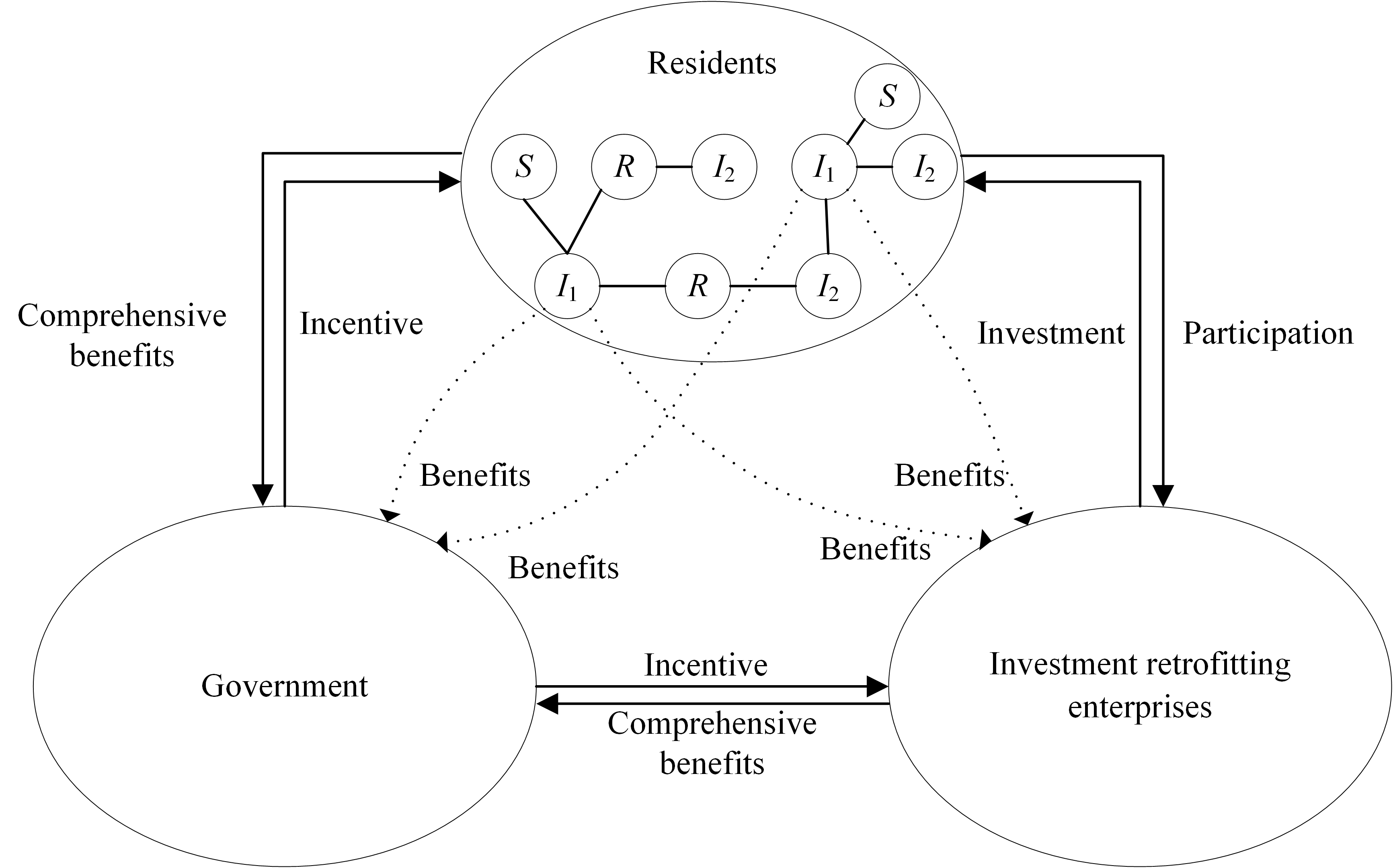

Supplement: S1 Fig — (ZIP) [file pone.0282314.s001.zip › S1_Figs/S1_Figs/Figs/Fig 1.tif]

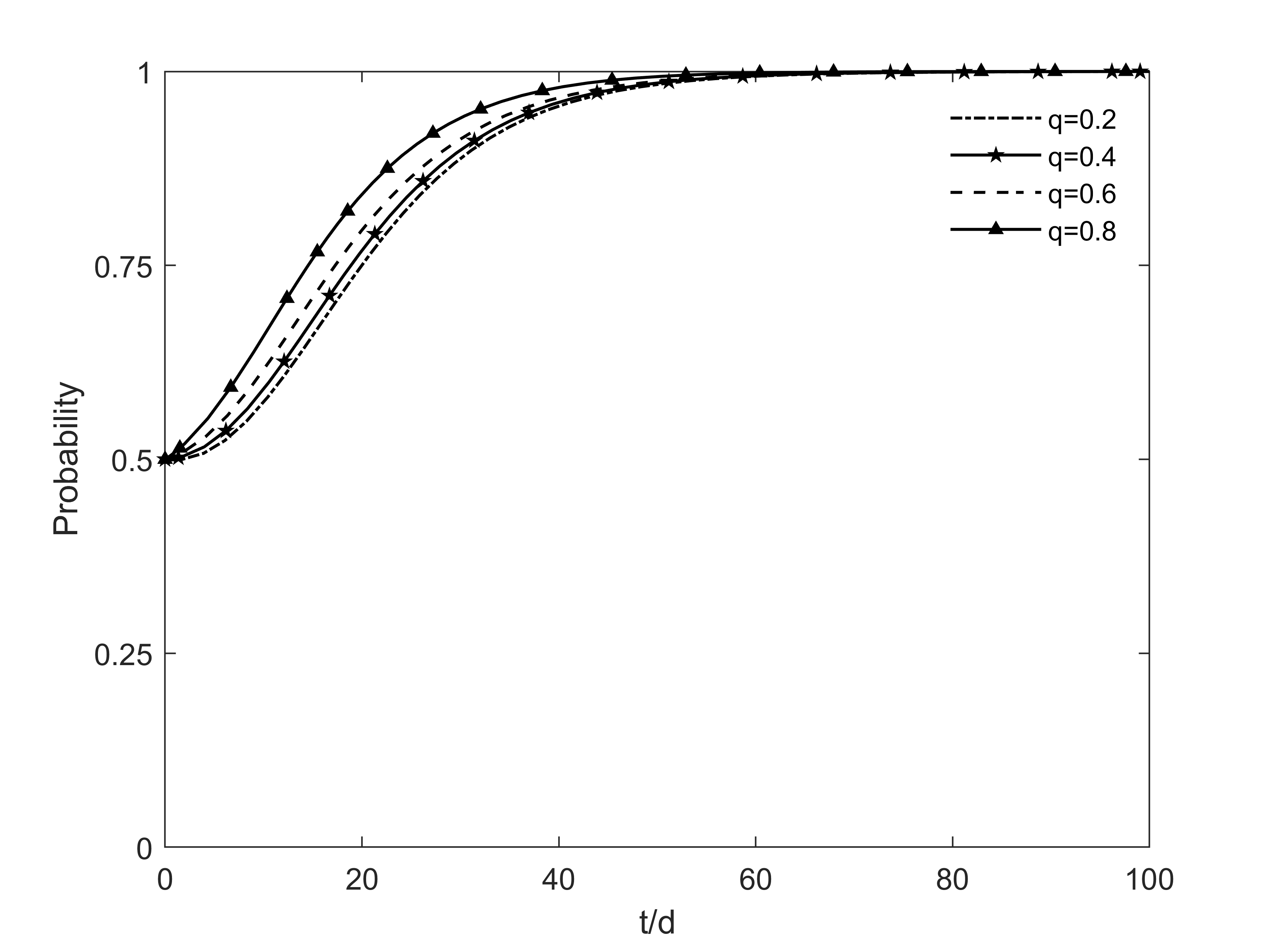

Supplement: S1 Fig — (ZIP) [file pone.0282314.s001.zip › S1_Figs/S1_Figs/Figs/Fig 10.tif]

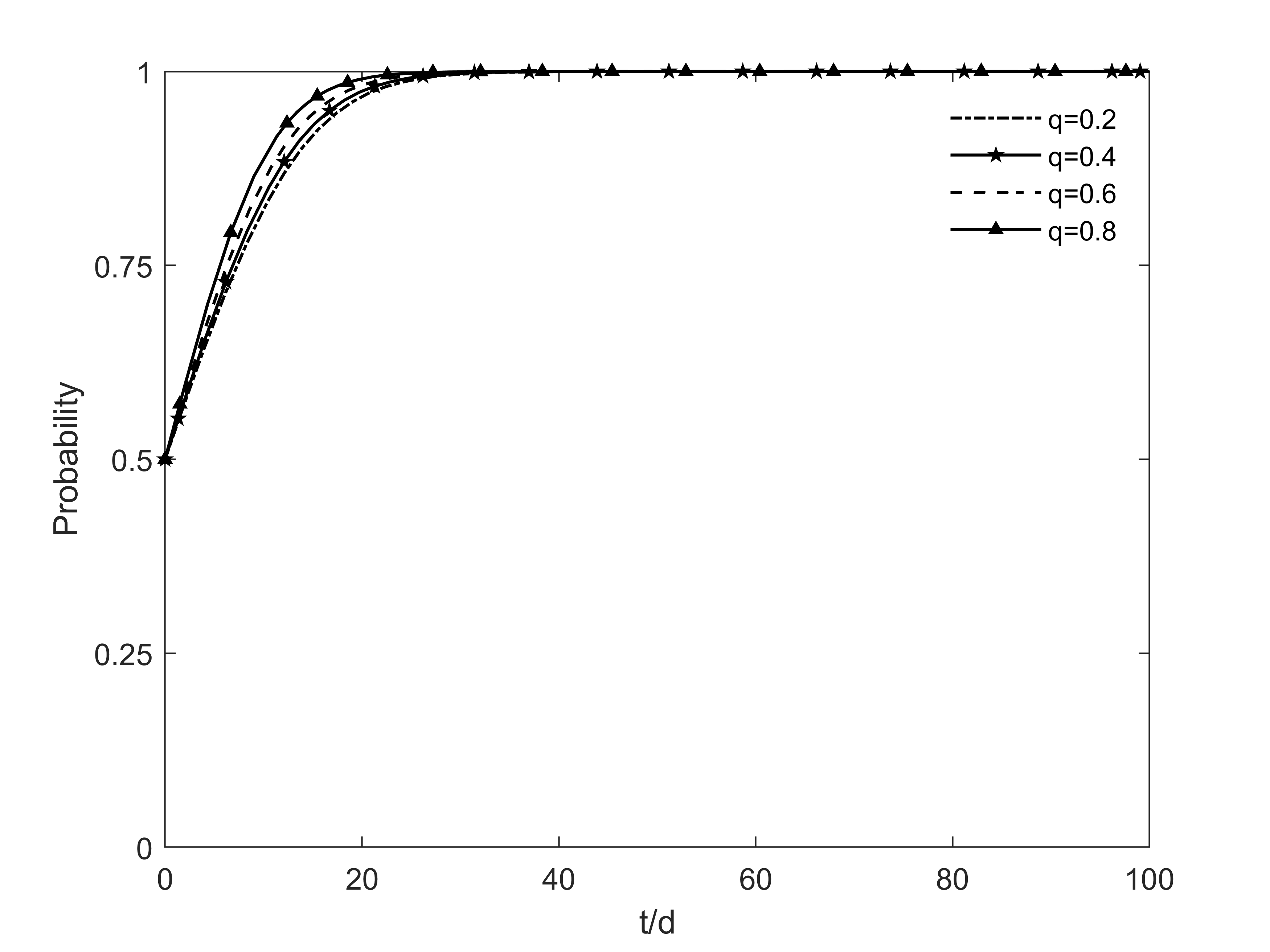

Supplement: S1 Fig — (ZIP) [file pone.0282314.s001.zip › S1_Figs/S1_Figs/Figs/Fig 11.tif]

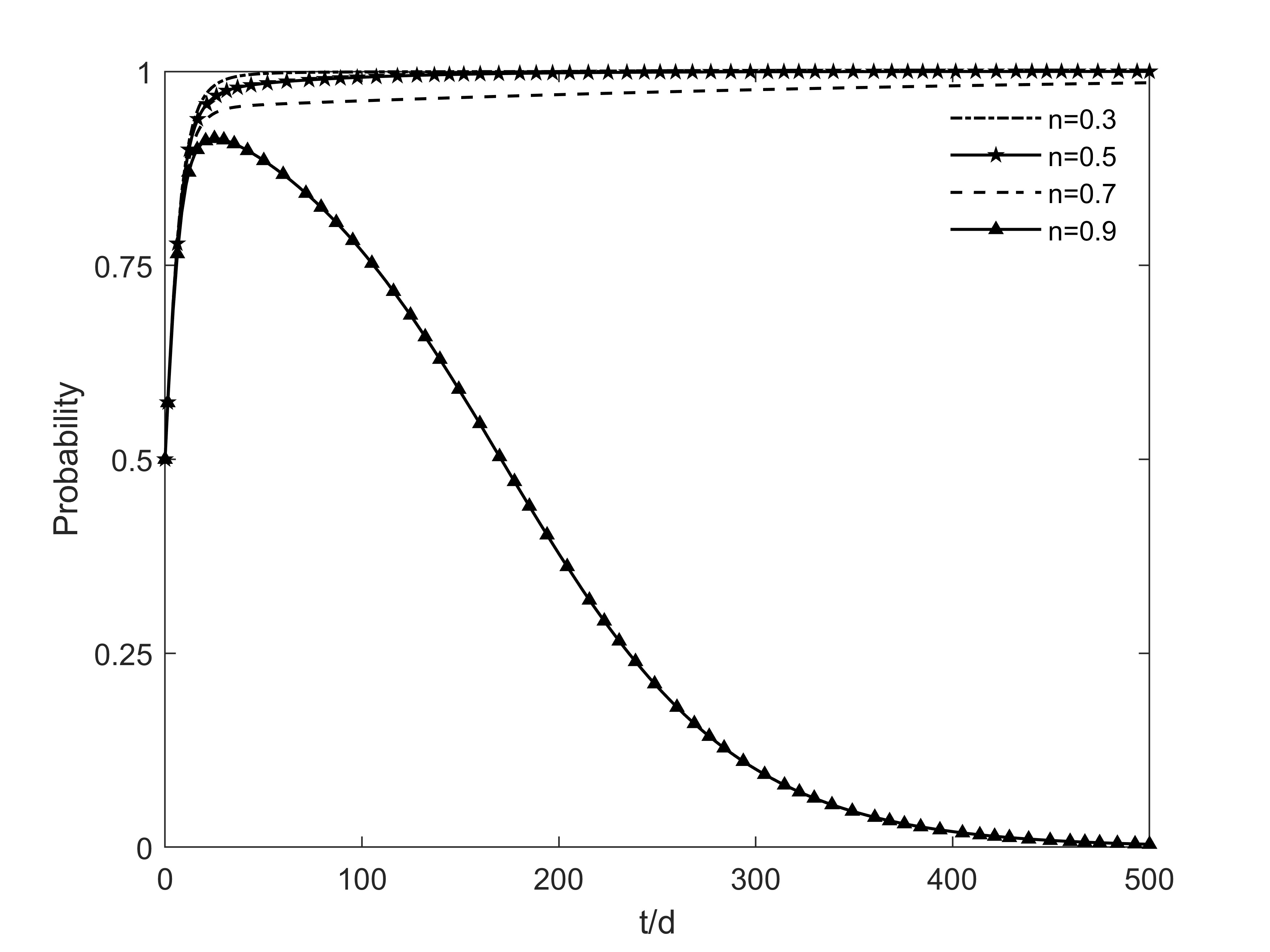

Supplement: S1 Fig — (ZIP) [file pone.0282314.s001.zip › S1_Figs/S1_Figs/Figs/Fig 12.tif]

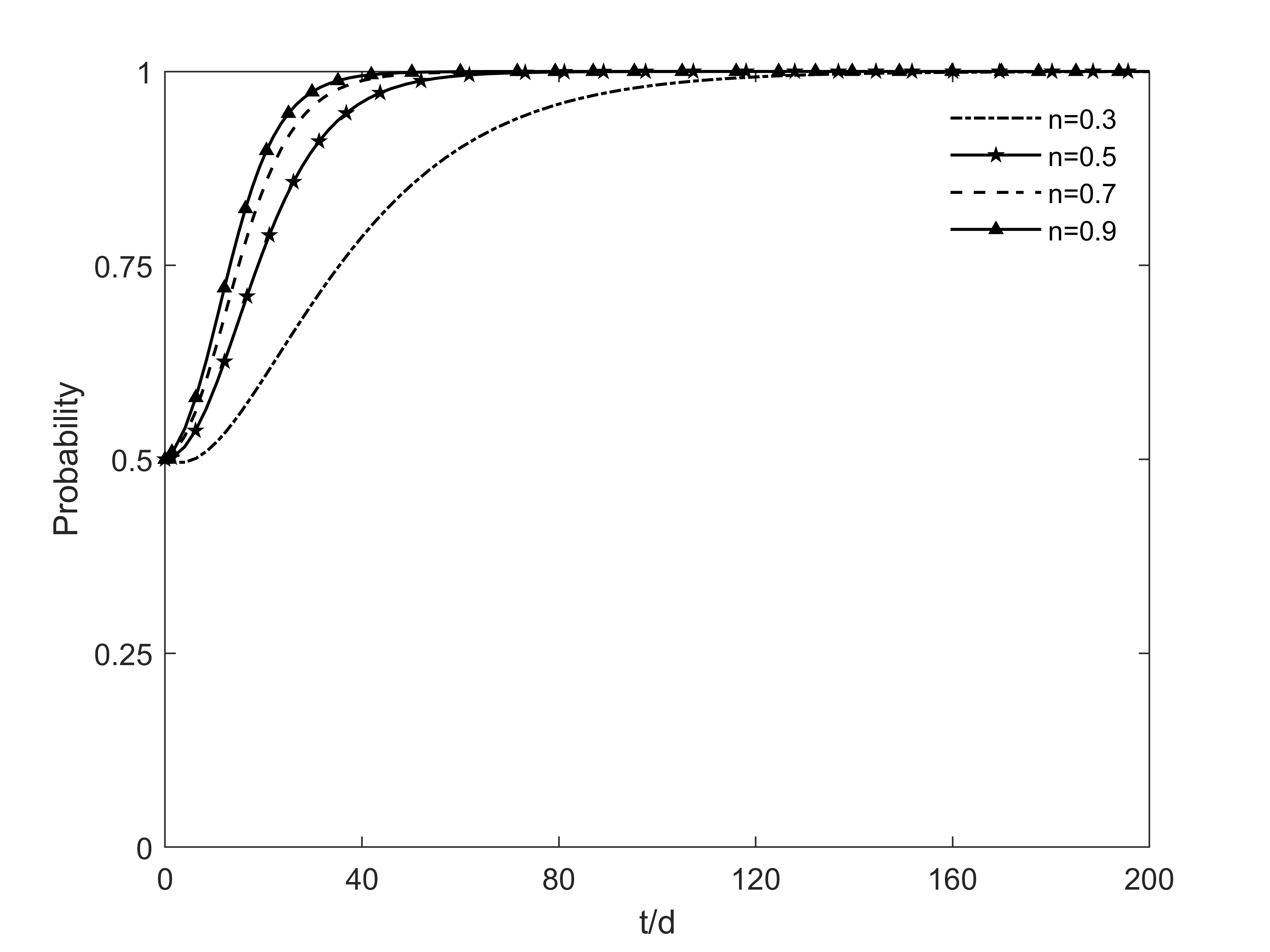

Supplement: S1 Fig — (ZIP) [file pone.0282314.s001.zip › S1_Figs/S1_Figs/Figs/Fig 13.tif]

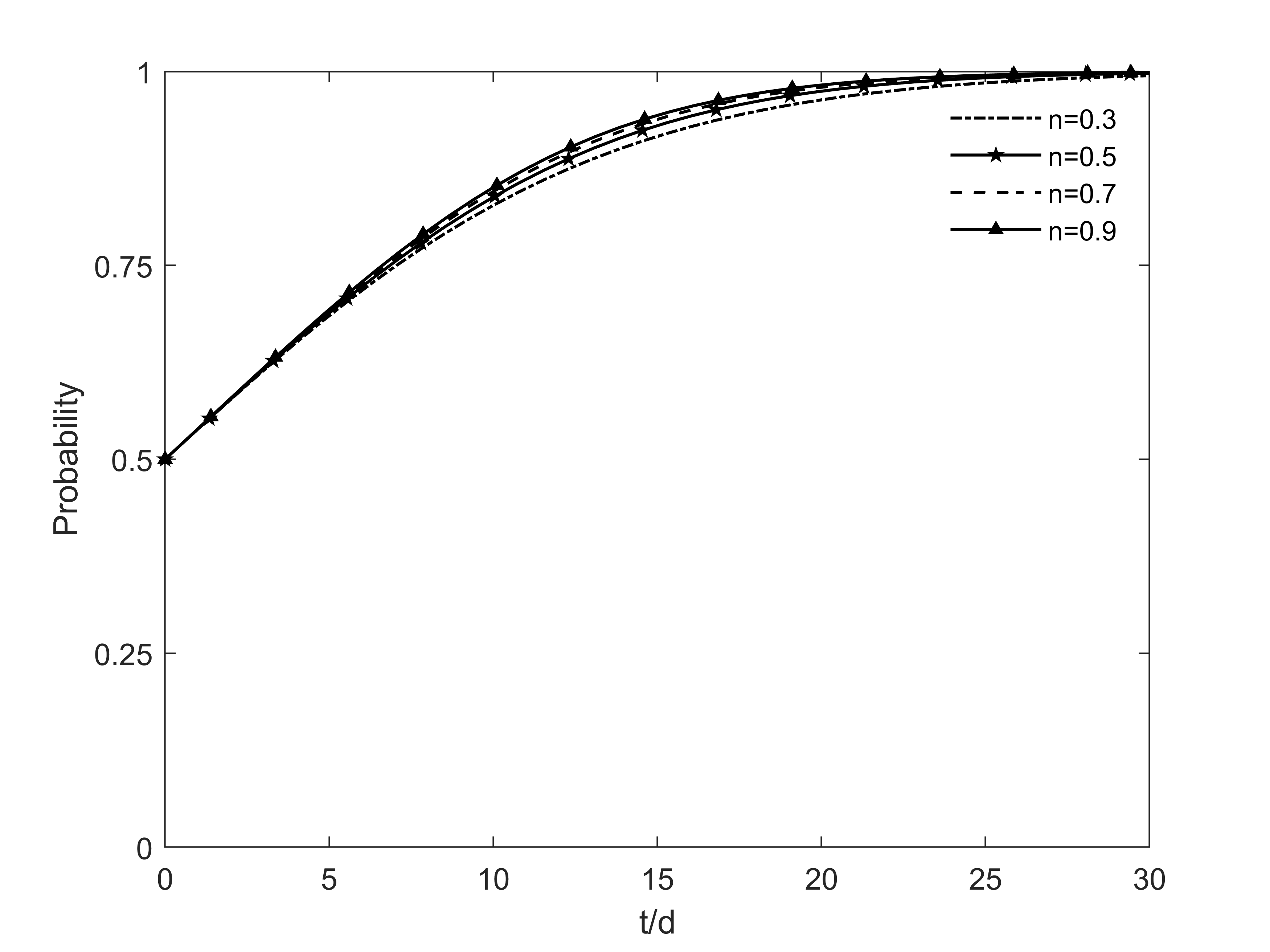

Supplement: S1 Fig — (ZIP) [file pone.0282314.s001.zip › S1_Figs/S1_Figs/Figs/Fig 14.tif]

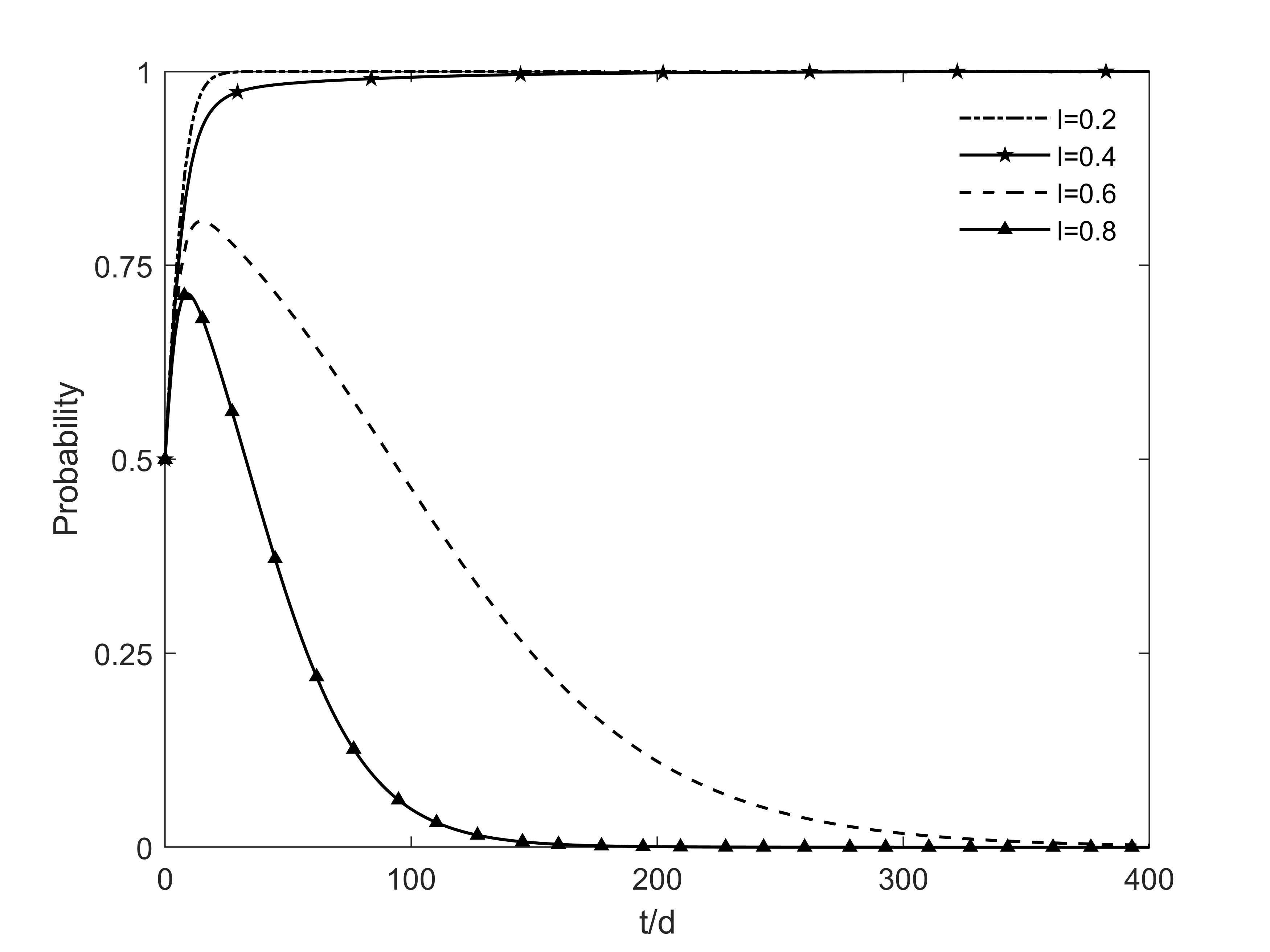

Supplement: S1 Fig — (ZIP) [file pone.0282314.s001.zip › S1_Figs/S1_Figs/Figs/Fig 15.tif]

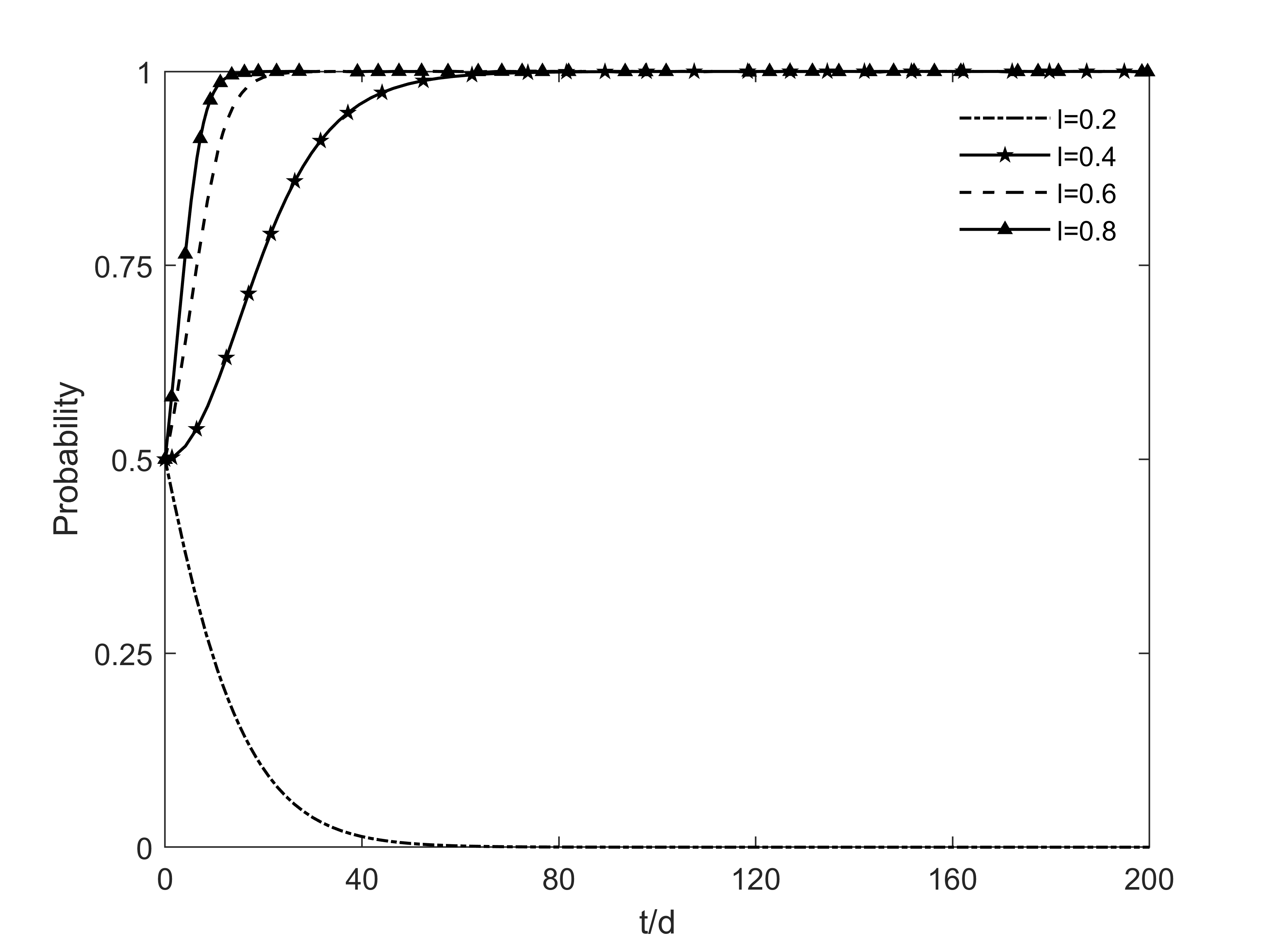

Supplement: S1 Fig — (ZIP) [file pone.0282314.s001.zip › S1_Figs/S1_Figs/Figs/Fig 16.tif]

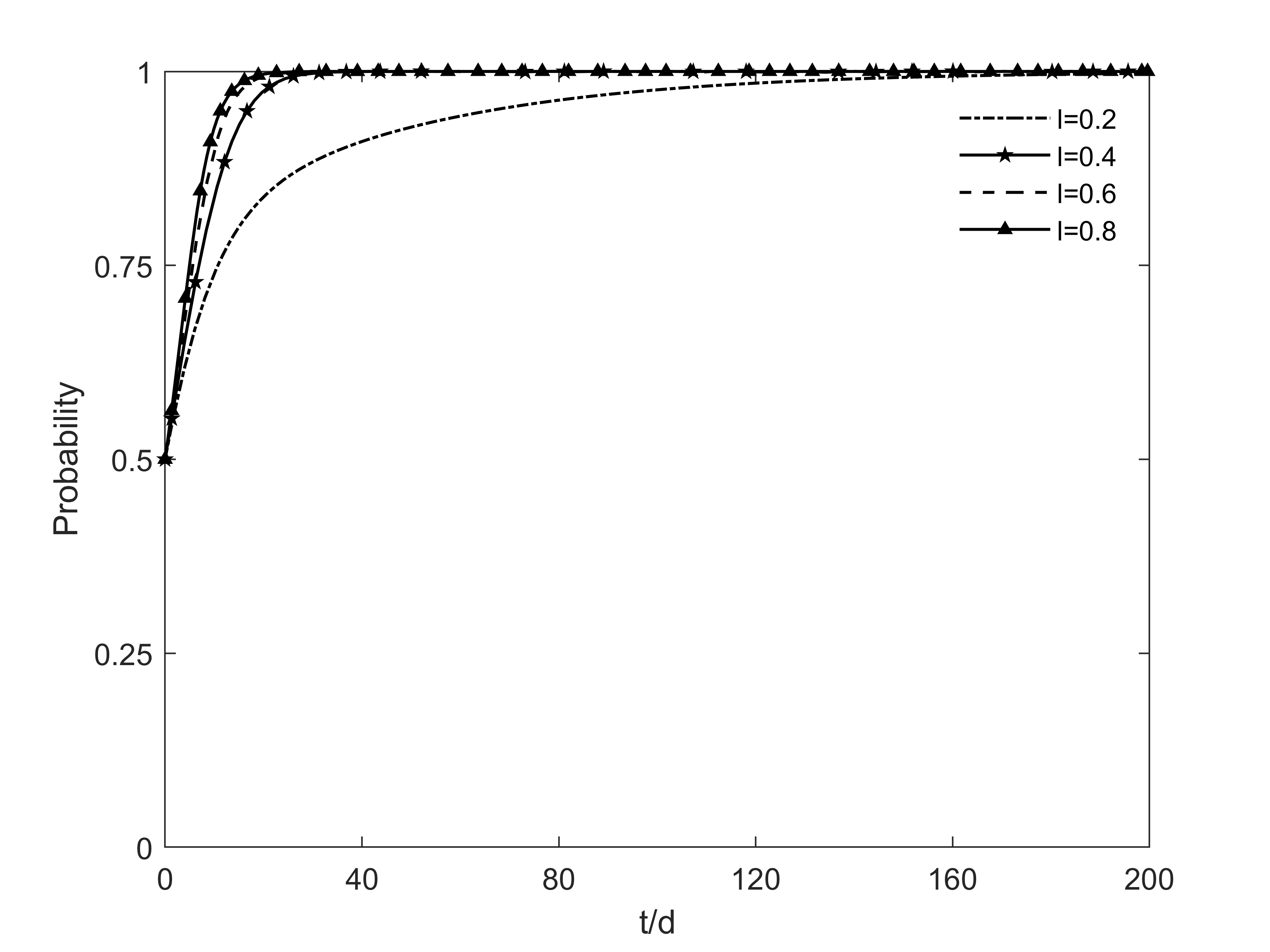

Supplement: S1 Fig — (ZIP) [file pone.0282314.s001.zip › S1_Figs/S1_Figs/Figs/Fig 17.tif]

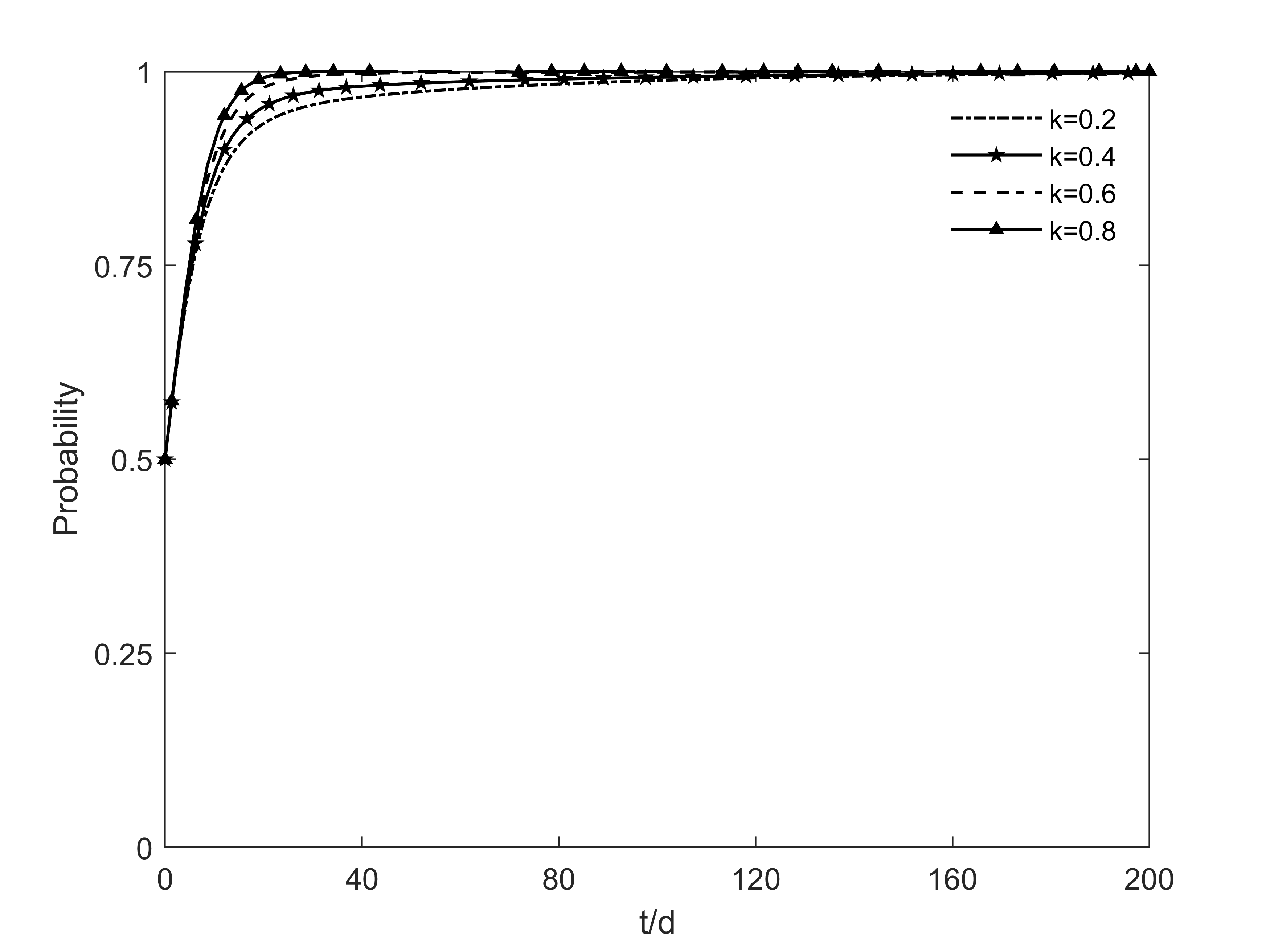

Supplement: S1 Fig — (ZIP) [file pone.0282314.s001.zip › S1_Figs/S1_Figs/Figs/Fig 18.tif]

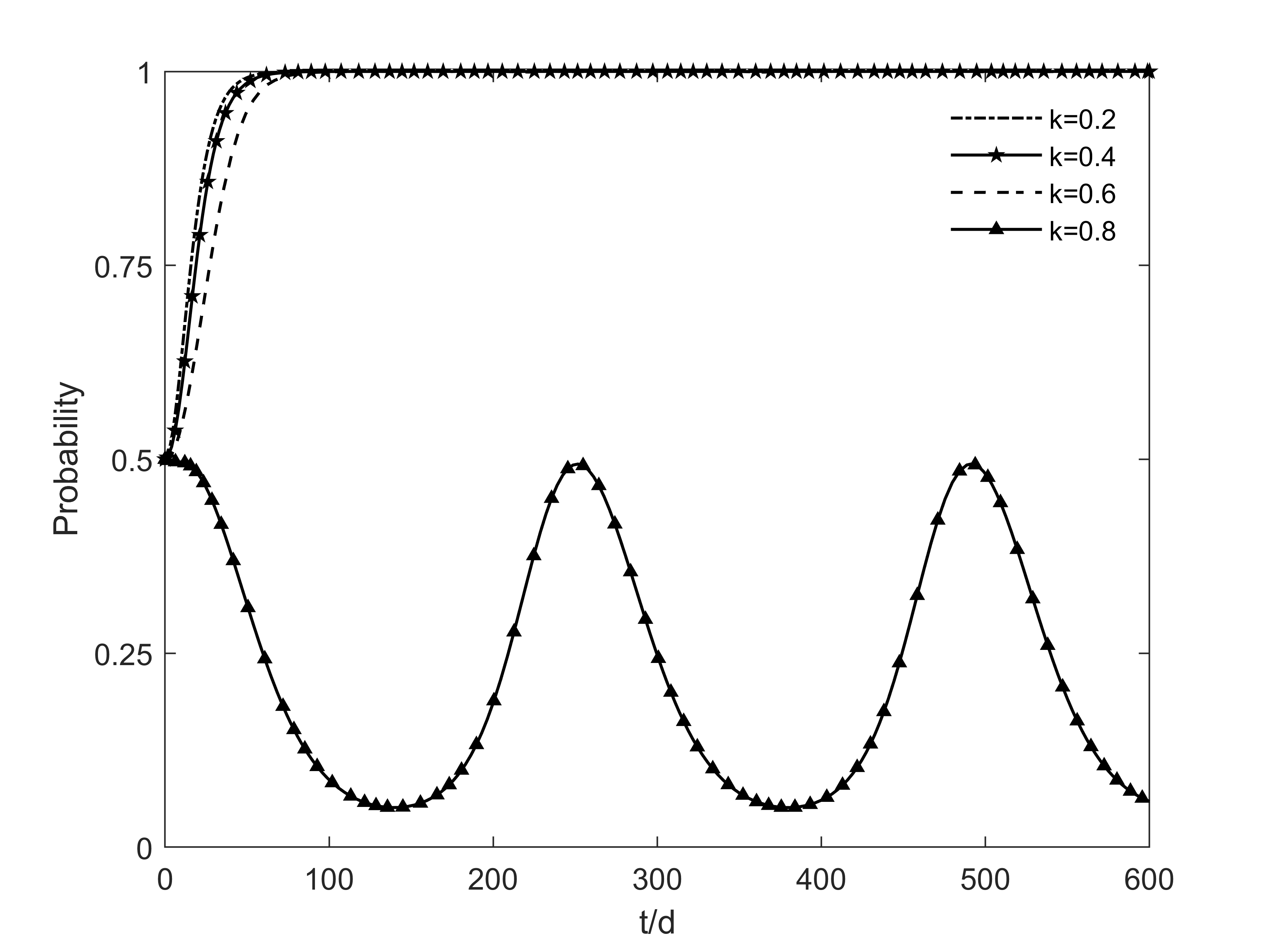

Supplement: S1 Fig — (ZIP) [file pone.0282314.s001.zip › S1_Figs/S1_Figs/Figs/Fig 19.tif]

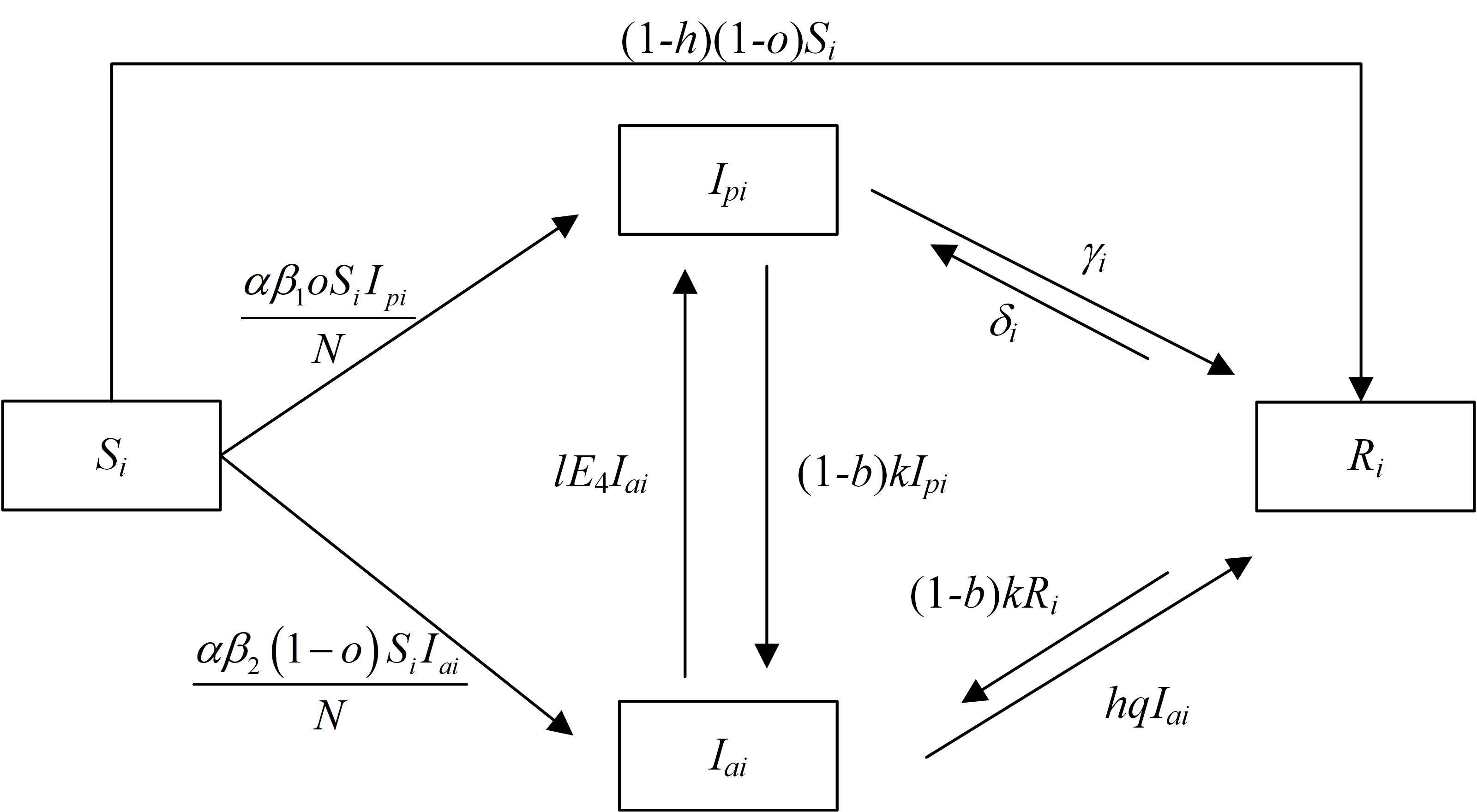

Supplement: S1 Fig — (ZIP) [file pone.0282314.s001.zip › S1_Figs/S1_Figs/Figs/Fig 2.tif]

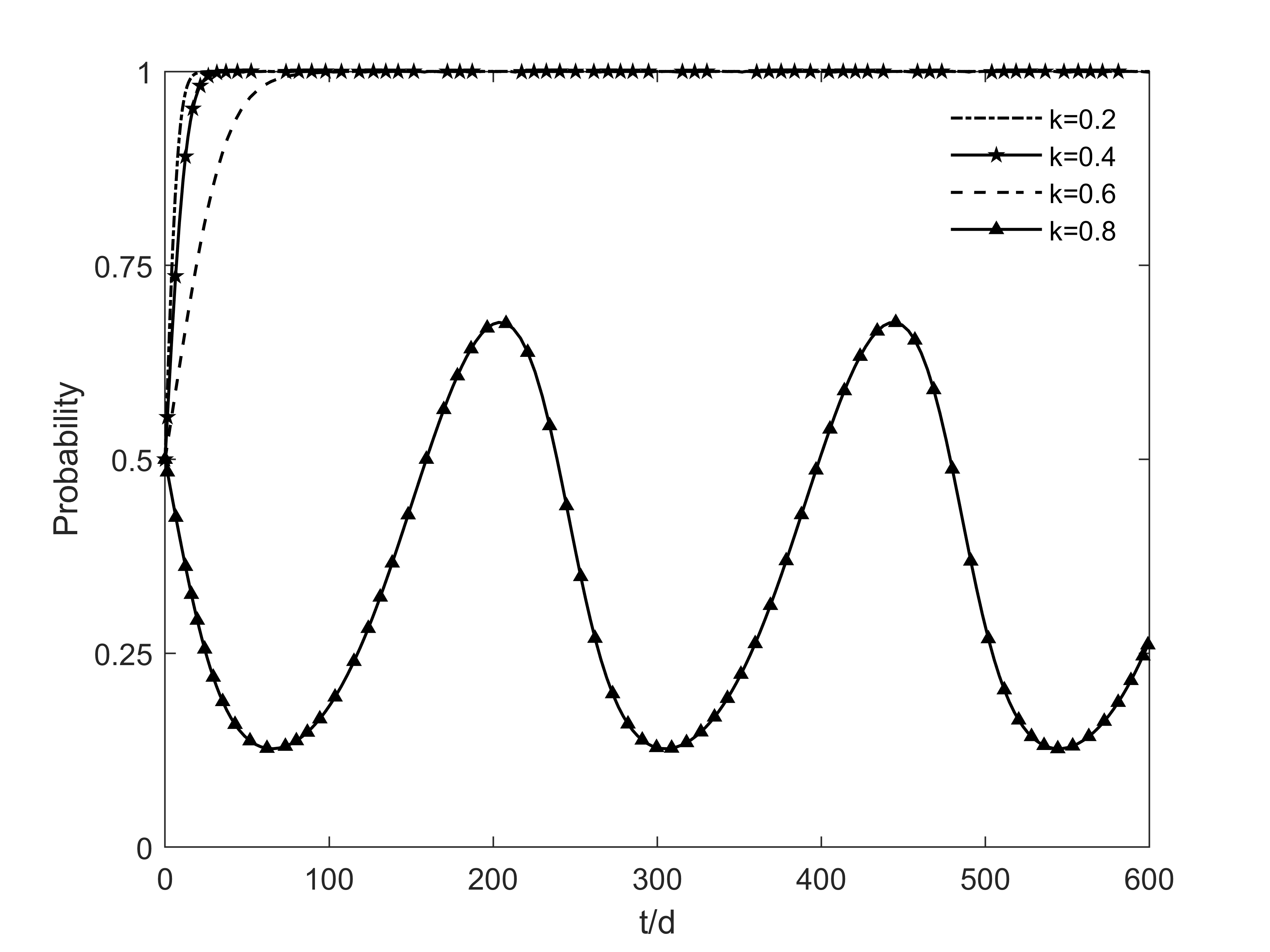

Supplement: S1 Fig — (ZIP) [file pone.0282314.s001.zip › S1_Figs/S1_Figs/Figs/Fig 20.tif]

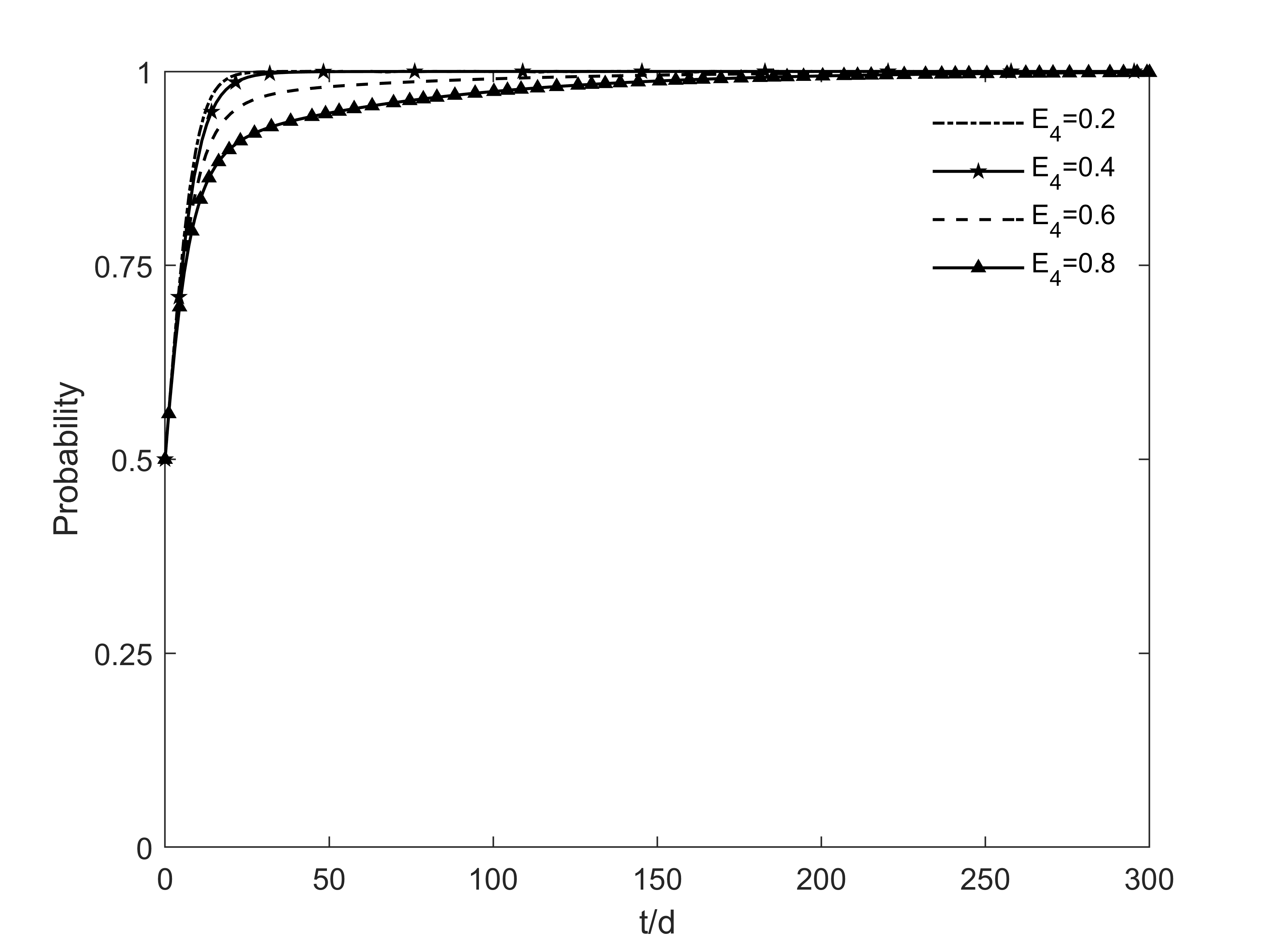

Supplement: S1 Fig — (ZIP) [file pone.0282314.s001.zip › S1_Figs/S1_Figs/Figs/Fig 21.tif]

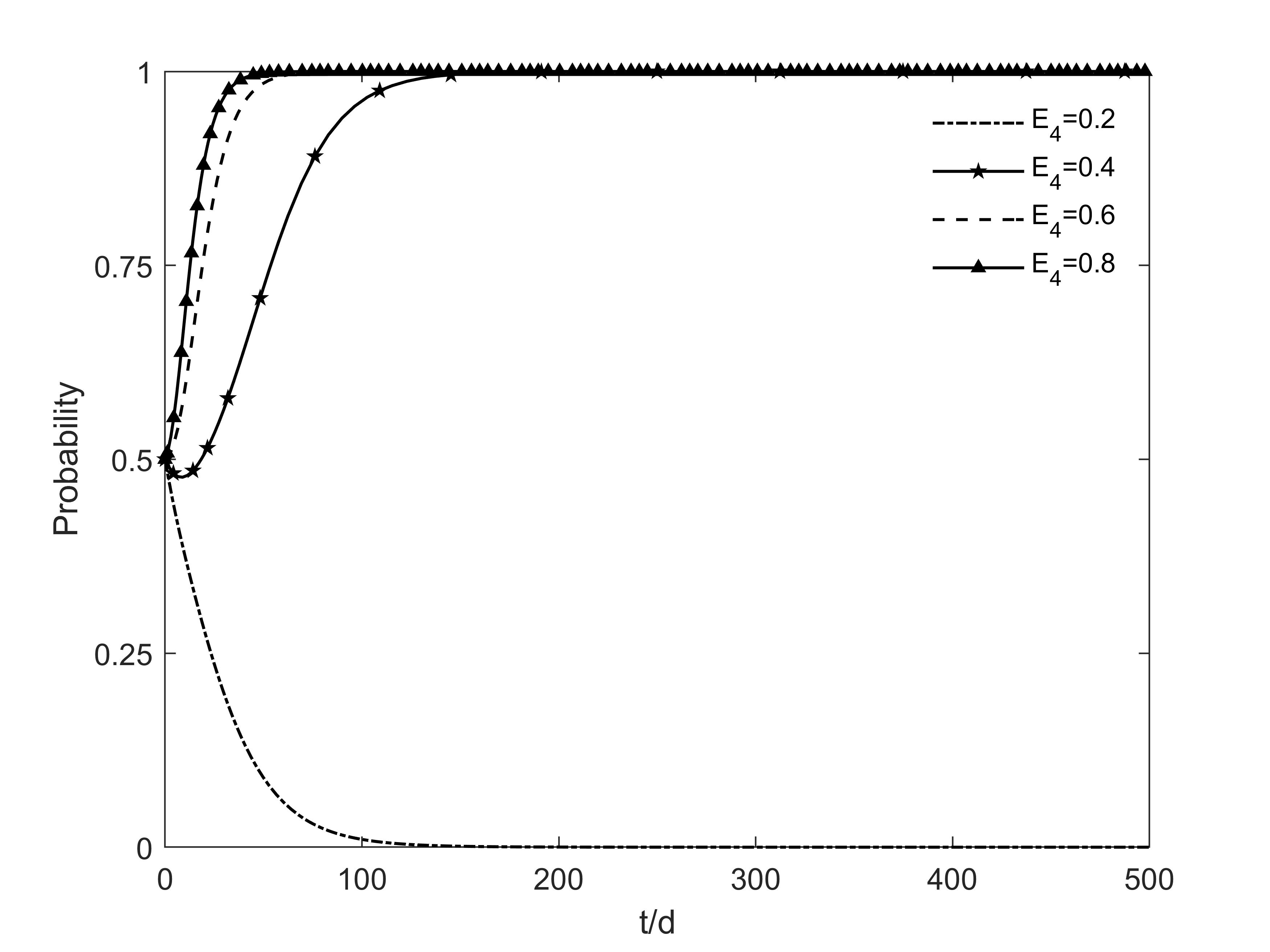

Supplement: S1 Fig — (ZIP) [file pone.0282314.s001.zip › S1_Figs/S1_Figs/Figs/Fig 22.tif]

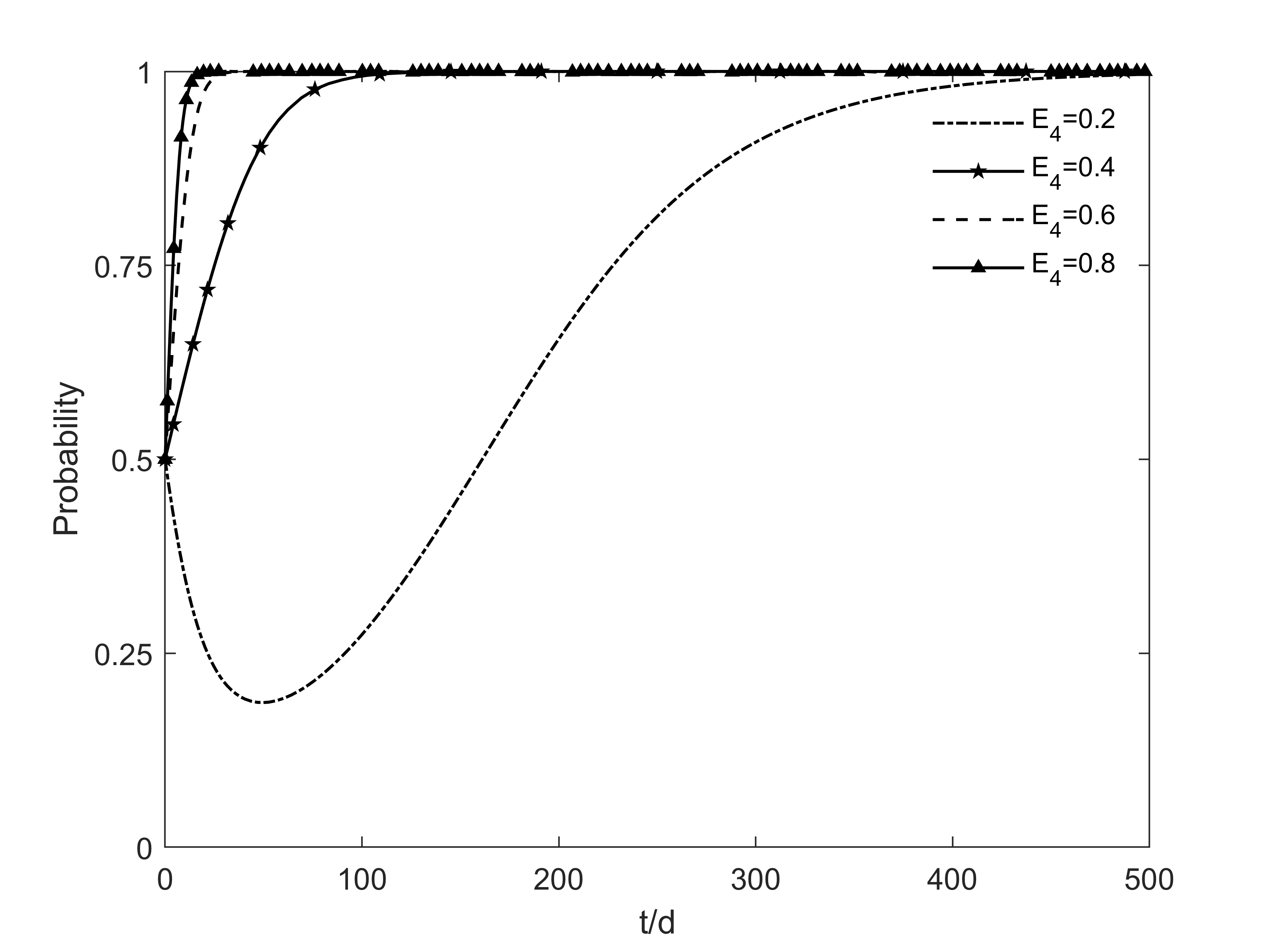

Supplement: S1 Fig — (ZIP) [file pone.0282314.s001.zip › S1_Figs/S1_Figs/Figs/Fig 23.tif]

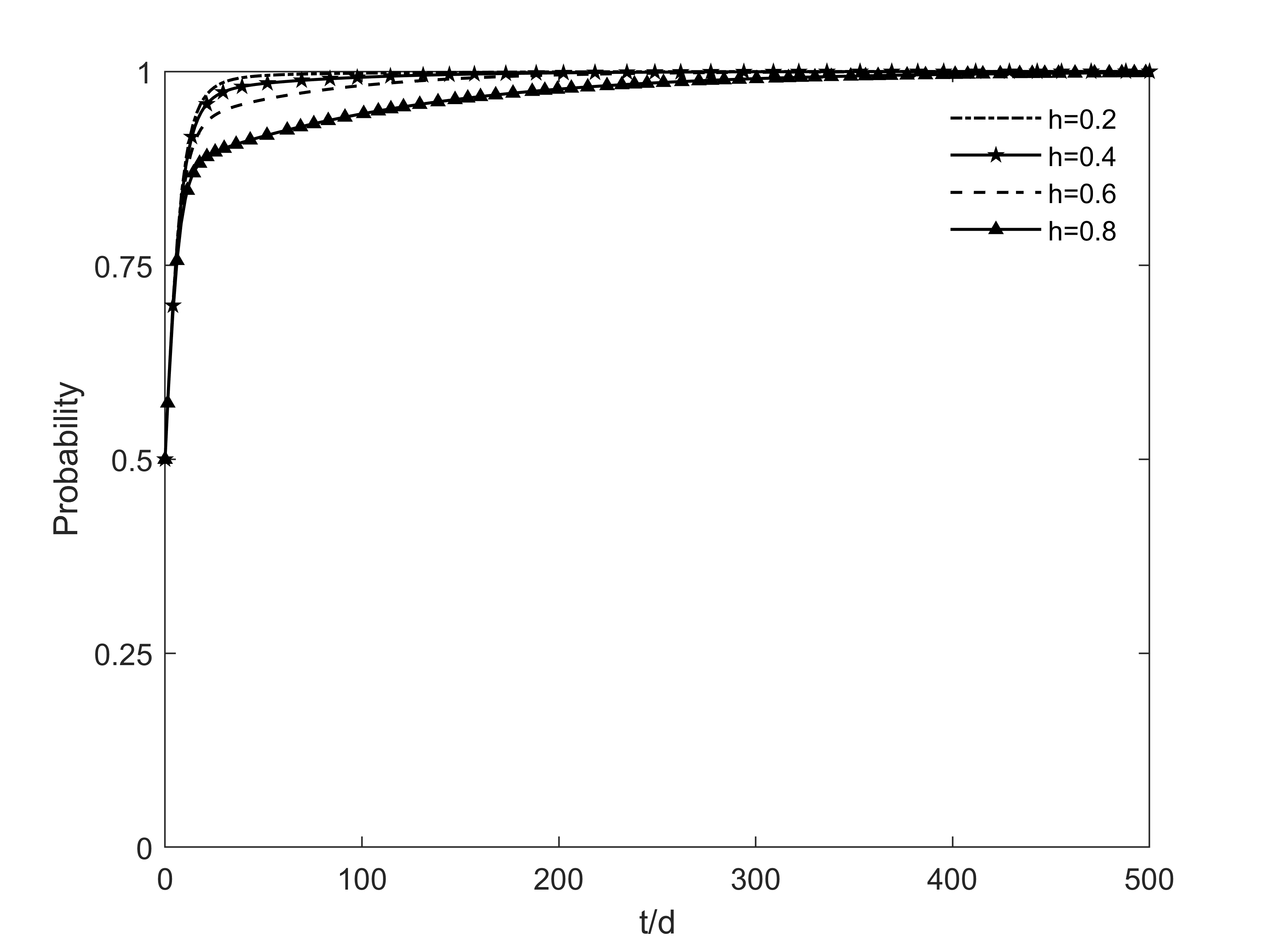

Supplement: S1 Fig — (ZIP) [file pone.0282314.s001.zip › S1_Figs/S1_Figs/Figs/Fig 3.tif]

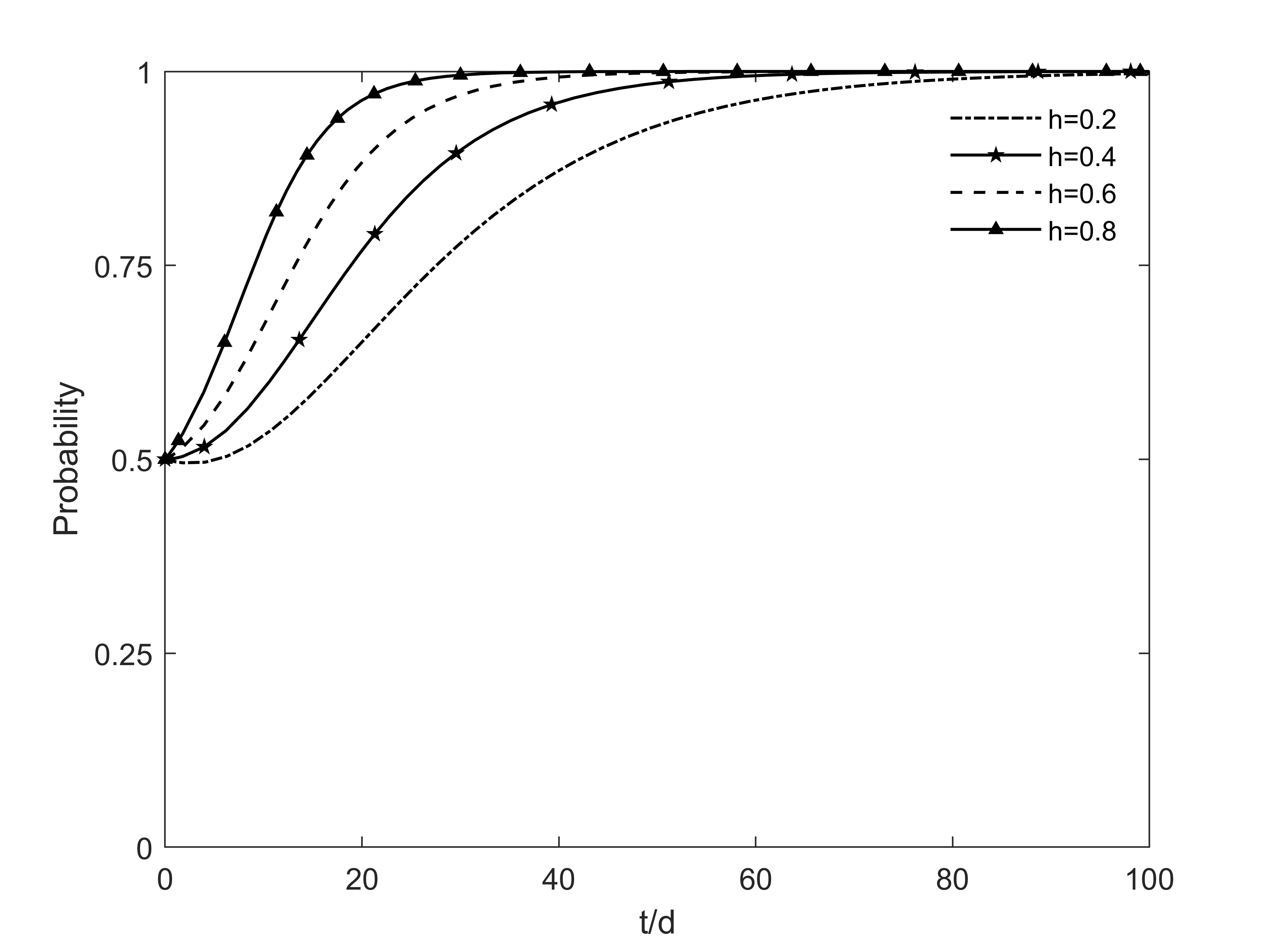

Supplement: S1 Fig — (ZIP) [file pone.0282314.s001.zip › S1_Figs/S1_Figs/Figs/Fig 4.tif]

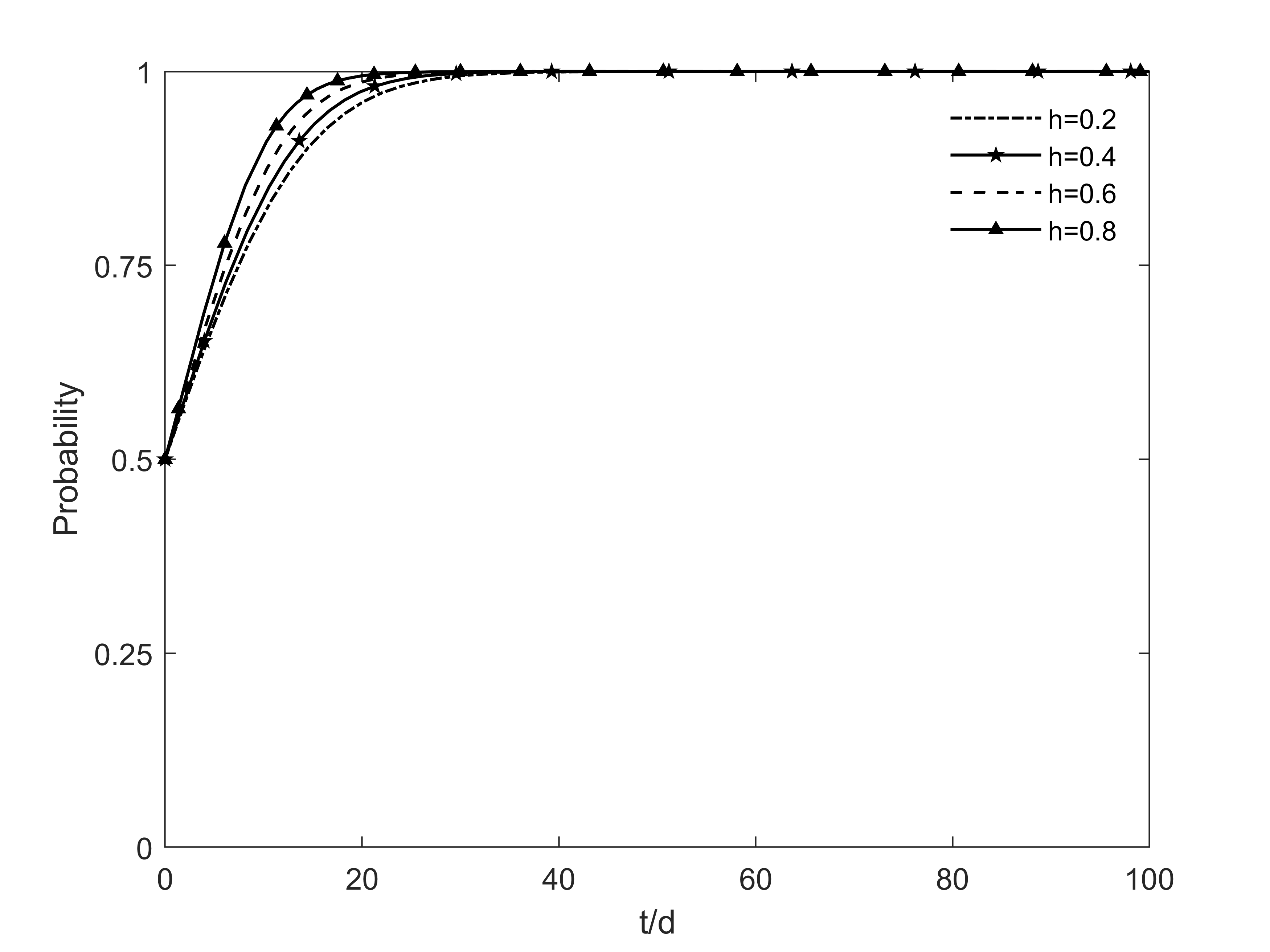

Supplement: S1 Fig — (ZIP) [file pone.0282314.s001.zip › S1_Figs/S1_Figs/Figs/Fig 5.tif]

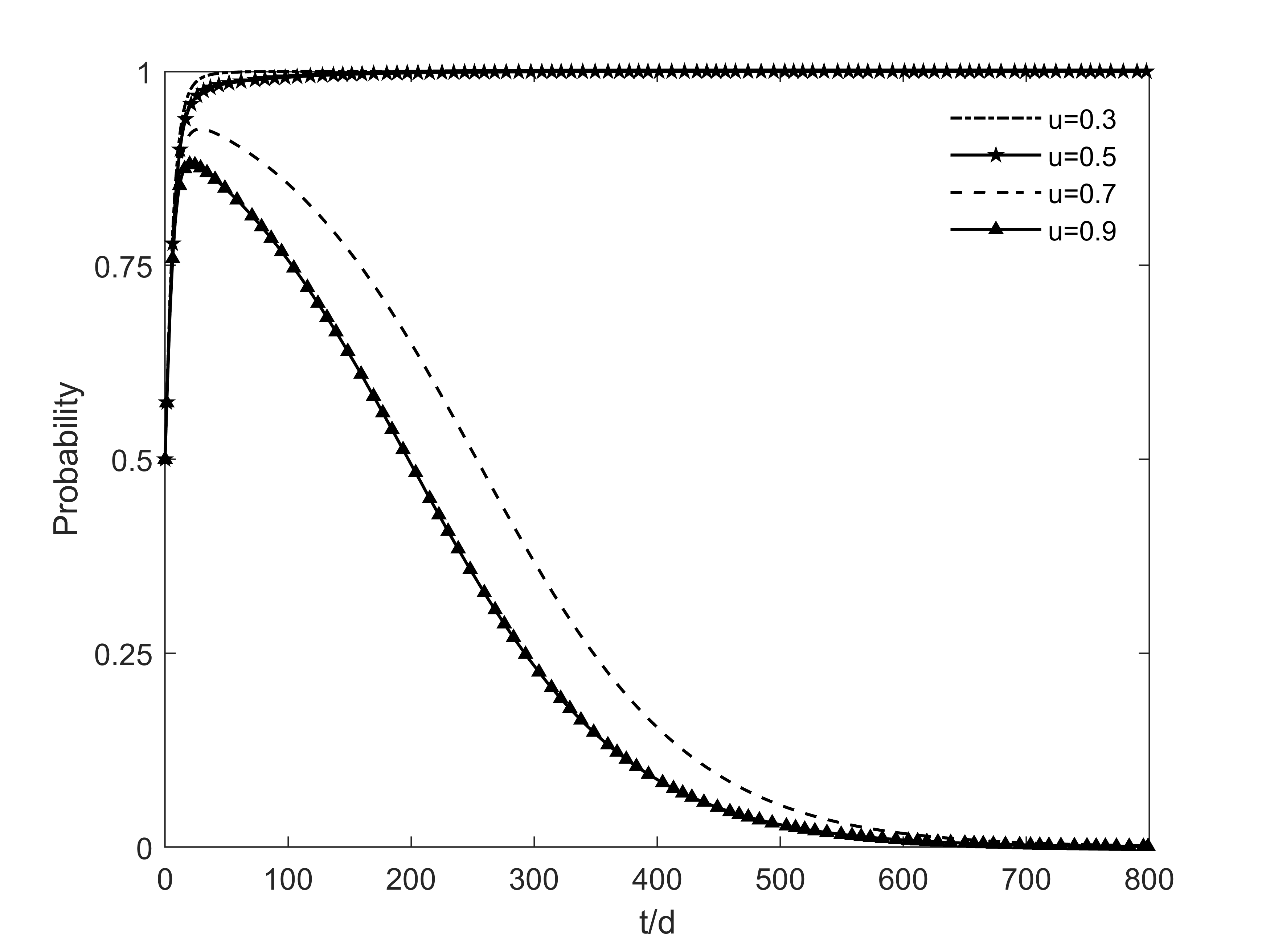

Supplement: S1 Fig — (ZIP) [file pone.0282314.s001.zip › S1_Figs/S1_Figs/Figs/Fig 6.tif]

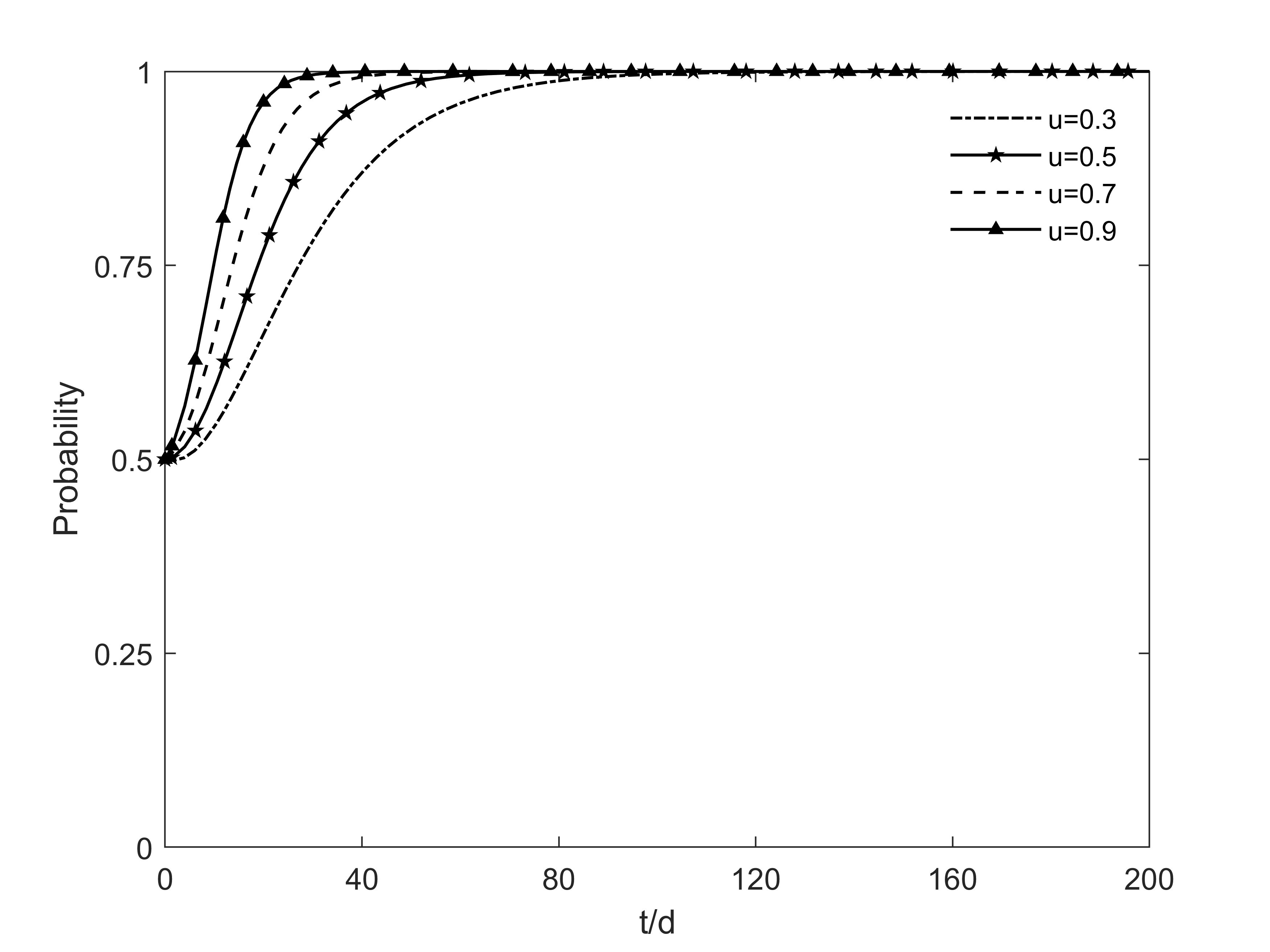

Supplement: S1 Fig — (ZIP) [file pone.0282314.s001.zip › S1_Figs/S1_Figs/Figs/Fig 7.tif]

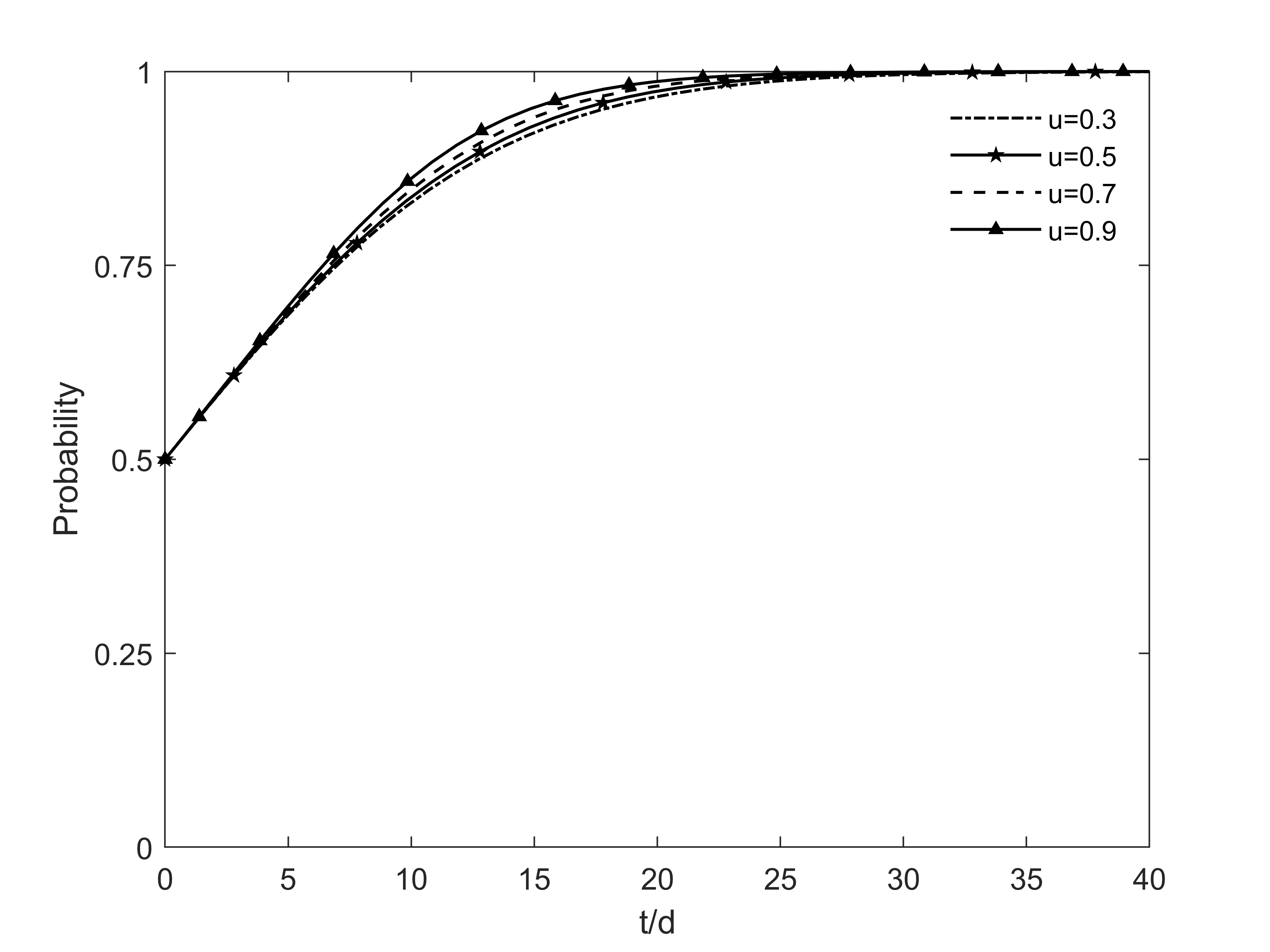

Supplement: S1 Fig — (ZIP) [file pone.0282314.s001.zip › S1_Figs/S1_Figs/Figs/Fig 8.tif]

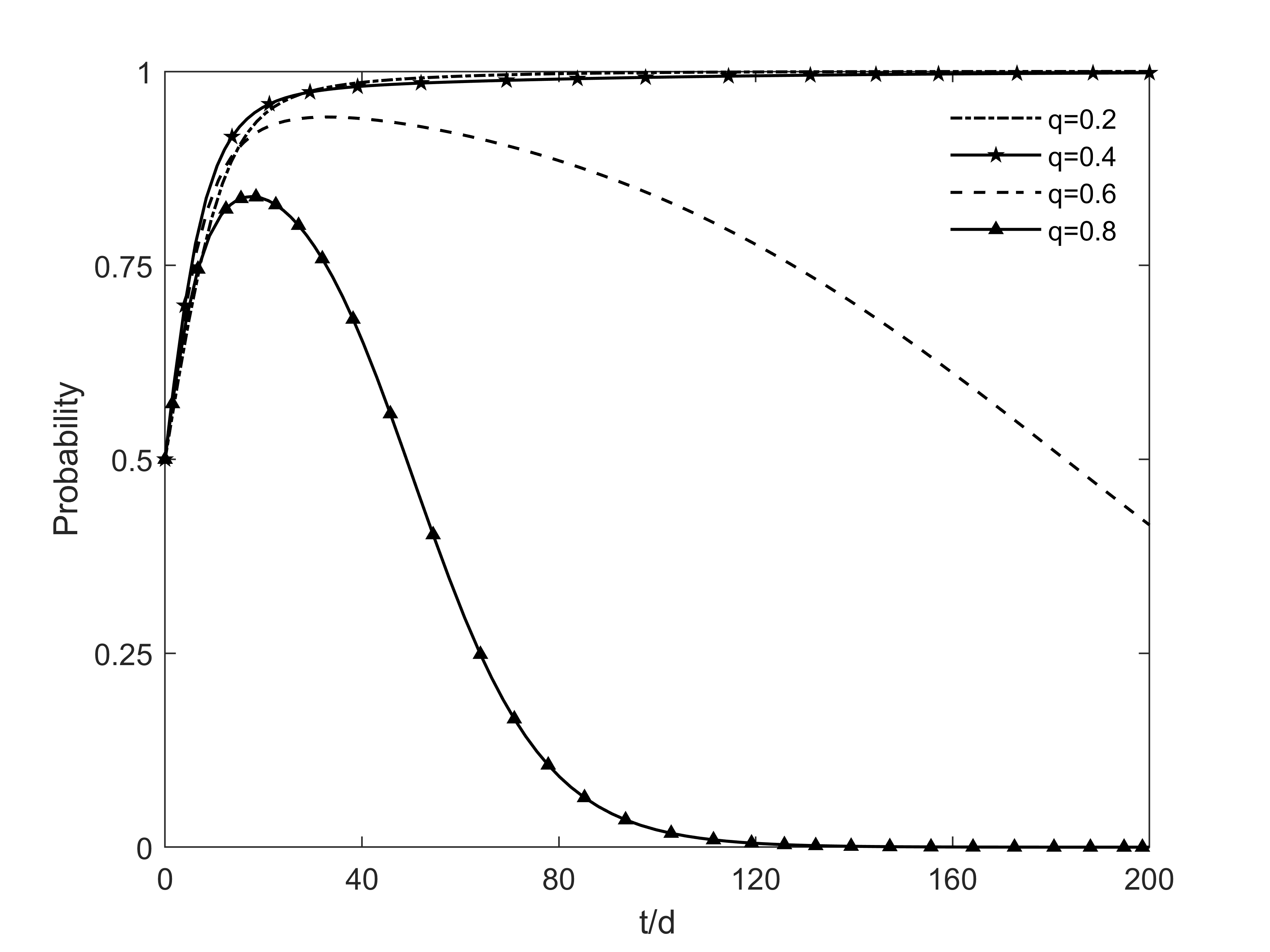

Supplement: S1 Fig — (ZIP) [file pone.0282314.s001.zip › S1_Figs/S1_Figs/Figs/Fig 9.tif]
